# *In silico* evolutionary analysis of *Helicobacter pylori* outer membrane phospholipase A (OMPLA)

**DOI:** 10.1186/1471-2180-12-206

**Published:** 2012-09-13

**Authors:** Hilde S Vollan, Tone Tannæs, Yoshio Yamaoka, Geir Bukholm

**Affiliations:** 1 Department of Clinical Molecular Biology and Laboratory Sciences (EpiGen), Division of Medicine, Akershus University Hospital, University of Oslo, Oslo, Norway; 2 Department of Medicine-Gastroenterology, Michael E. DeBakey Veterans Affairs Medical Center and Baylor College of Medicine, Houston, TX, USA; 3 Institute of Health and Society, University of Oslo, Oslo, Norway; 4 Centre for Laboratory Medicine, Østfold Hospital Trust, Fredrikstad, Norway

**Keywords:** OMPLA, *pldA*, Phospholipase A, Outer membrane, *Helicobacter pylori*, Biogeography, Adaptation

## Abstract

**Background:**

In the past decade, researchers have proposed that the *pldA* gene for outer membrane phospholipase A (OMPLA) is important for bacterial colonization of the human gastric ventricle. Several conserved *Helicobacter pylori* genes have distinct genotypes in different parts of the world, biogeographic patterns that can be analyzed through phylogenetic trees. The current study will shed light on the importance of the *pldA* gene in *H. pylori. In silico* sequence analysis will be used to investigate whether the bacteria are in the process of preserving, optimizing, or rejecting the *pldA* gene. The *pldA* gene will be phylogenetically compared to other housekeeping (HK) genes, and a possible origin via horizontal gene transfer (HGT) will be evaluated through both intra- and inter-species evolutionary analyses.

**Results:**

In this study, *pldA* gene sequences were phylogenetically analyzed and compared with a large reference set of concatenated HK gene sequences. A total of 246 *pldA* nucleotide sequences were used; 207 were from Norwegian isolates, 20 were from Korean isolates, and 19 were from the NCBI database. Best-fit evolutionary models were determined with MEGA5 ModelTest for the *pldA* (K80 + I + G) and HK (GTR + I + G) sequences, and maximum likelihood trees were constructed. Both HK and *pldA* genes showed biogeographic clustering*.* Horizontal gene transfer was inferred based on significantly different GC contents, the codon adaptation index, and a phylogenetic conflict between a tree of OMPLA protein sequences representing 171 species and a tree of the AtpA HK protein for 169 species. Although a vast majority of the residues in OMPLA were predicted to be under purifying selection, sites undergoing positive selection were also found.

**Conclusions:**

Our findings indicate that the *pldA* gene could have been more recently acquired than seven of the HK genes found in *H. pylori*. However, the common biogeographic patterns of both the HK and *pldA* sequences indicated that the transfer occurred long ago. Our results indicate that the bacterium is preserving the function of OMPLA, although some sites are still being evolutionarily optimized.

## Background

More than half of the world’s population is colonized with *Helicobacter pylori*[1]. Colonization usually occurs in early childhood and results in disease in about 10% of cases [2]. This disease will in most cases be diagnosed as gastric or duodenal ulcers, while some cases will be diagnosed as gastric cancer [3].

The human gastric ventricle is the only known natural habitat for *H. pylori*, and one bacterial strain usually establishes a chronic, lifelong, persistent colonization in one individual [4]. *Helicobacter pylori* has a high level of sequence variation and has therefore been referred to as a quasi-species [5-7]. Natural transformation by exogenous DNA [8,9], mutations, and recombinations are probably important mechanisms for *H. pylori* adaption and survival; for example, a variable genome could give advantages in evading the host’s immune system. In spite of the high sequence variation observed in *H. pylori*, 1237 core genes have been described that are common to the analyzed *H. pylori* genomes. The amino acid identities range between 65-100%. Among these core genes are housekeeping (HK) genes that are essential for *H. pylori* survival, and the genetic variability in these genes remains very low [10,11]. This conservation is reflected in phylogenetic analysis, where HK genes have been used to trace human migration, indicating co-evolution between *H. pylori* and its host. Linz *et al*. traced *H. pylori* infection in humans to before their migration from Africa through sequence analysis [11,12].

Analyses of conserved *H. pylori* genes indicate the evolution of distinct genotypes in different parts of the world. The virulence factor cytotoxin-associated gene A protein, CagA, shows biogeographic variation. Yamaoka *et al.* postulated that the geographical differences that are observed in the incidence of gastric cancer could be explained by different *H. pylori* strains (with regard to the distribution of *cagA* and *vacA* genotype) [13]. CagA is injected in the host cell through the Type IV secretion system (T4SS) which is coded by Cag Pathogenicity Island (*cagPAI*) genes. These genes are also involved in horizontal gene transfer (HGT). Genes integrated into the *H. pylori* genome via HGT may have originated from either other bacteria or eukaryotic cells [14]. Olbermann *et al.*[15] analyzed the selection pressure for *cagPAI* genes and found that one-third of the genes were under positive selection. Most of the genes under positive selection, including the *cagA* gene, code for surface-exposed proteins. In positive selection, mutations increase fitness and, thus, new alleles increase in frequency in the population. In neutral (or nearly neutral) selection, mutations have no drastic effect on fitness and increase or decrease in frequency by chance. When fitness decreases due to deleterious mutations, new alleles are removed through purifying selection (i.e. *virD4* and *virB11* found in T4SS) [15].

Several authors have proposed that the *pldA* gene (coding for outer membrane phospholipase A, OMPLA) is important for the ability of the bacterium to colonize the human gastric ventricle [16,17]. Tannæs *et al.*[18] characterized a classical phase-variation in this gene due to DNA slippage in a homopolymeric tract that results in either a complete (*pldA*ON) or truncated protein (*pldA*OFF). The homopolymeric tract was found in all of the clinical isolates of *H. pylori* sequenced by Tannæs *et al.*[18]. The conservation of the homopolymeric tract in this gene through phylogenesis underlines the importance of the gene product and maintenance of the phase variation for this bacterium. This study investigated the evolution of the *pldA* gene in *H. pylori. In silico* sequence analysis was used to determine whether the bacteria were in the process of preserving, optimizing, or perhaps even rejecting the *pldA* gene. Sequences of *pldA* were compared by both identity and phylogenetic analysis to a reference set of HK genes from a large number of isolates sequenced by Falush *et al.*[11]. Horizontal gene transfer prediction was carried out via both intra- and inter-species phylogenetic analysis using related taxa and the estimation of both codon bias and GC content in *H. pylori* isolates.

## Results

### *CagA* EPIYA genotyping

All of the 20 Korean sequences had an East Asian *cagA* ABD genotype. Nearly all of the 50 isolates analyzed from Norway had Western *cagA* genotypes, with the following distribution: 66% ABC, 12% ABCC, 12% AB, 4% ABCCC, and 2% AC. The two isolates collected from patients with East Asian origins displayed a *cagA* ABD genotype (4%).

### Amplification of *vacA*

One Norwegian isolate from a patient of North-African origin was *VacA* genotyped. The sample contained an s1b allele and the m1 mid-region type.

### Bioinformatic analyses of *H. pylori pldA* and seven core housekeeping genes

Gene evolution was assessed by comparing *H. pylori pldA* gene sequences to concatenated core HK genes. The average pairwise sequence identity was 97.26% ± 0.01 for the *pldA* sequences and 95.60% ± 0.01 for the HK genes. The average genetic distance of the *pldA* genes was 0.03, while it was 0.05 for the concatenated HK genes.

**Figure 1:**
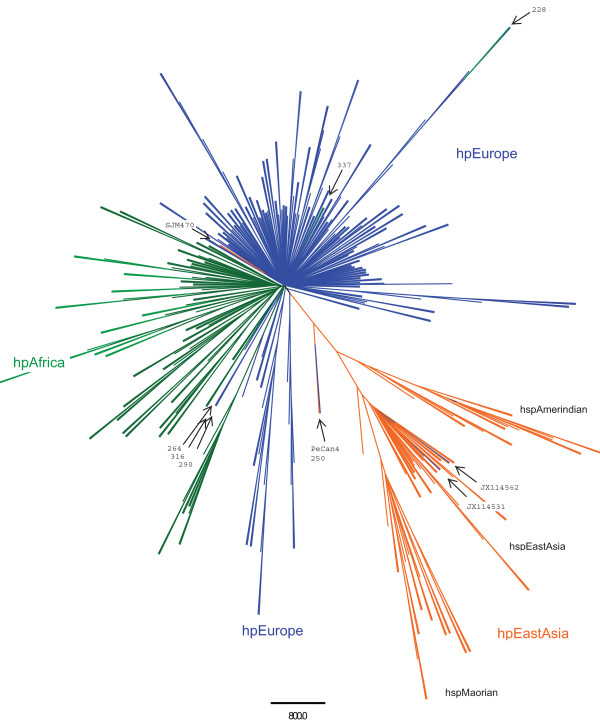
**Phylogenetic tree of* Helicobacter pylori *housekeeping sequences.** The seven concatenated HK genes were biogeographically classified: blue represents European strains (hpEurope), orange indicates the East Asian (hpEastAsia which includes the subpopulations hspAmerindian, hspEastAsian and hspMaorian) isolates, and green denotes African (hpAfrica) strains. The outliers are identified by black arrows (see Discussion for more information). Additional file 3: Table S1 contain label with corresponding MLST/GenBank ID. See Additional file 7: Figure S1 for complete labeling. This radial tree of 393 sequences is the majority rule consensus of 1000 maximum likelihood bootstrap replicates analyzed in PhyML with the GTR + I + G model and visualized in FigTree (see Methods for more details).

The phylogenetic reference tree of concatenated HK genes is shown in Figure 1. With a few exceptions, the sequences clustered as expected according to geographic region. In this phylogenetic tree, the majority of sequences were from European isolates. They were separated into two clades by the African and East Asian isolates. The East Asian cluster could be further subdivided into Maorian, East Asian, and Amerindian sequences. Two isolates collected in Norway grouped in the East Asian subcluster; these patients were of East Asian origin. As expected, the remaining two samples originating from Norway were found in the European cluster in the reference tree*.* Pecan4 was isolated from a Peruvian patient and thus initially classified as an Amerindian strain, however, it does not cluster with the other Amerindians in the East Asian cluster as was observed by Kawi *et al*. [19]. Two isolates in our tree were described by Falush as hpAfrica but clustered with European sequences, and both patients were Cape Colored or Mezito, with European ancestors. Four outliers were not found in the European cluster [20]. The remaining outliers consisted of two South African samples and one Piaroa isolate. The Maorian and Amerindian sequences formed a subcluster with the highest branch support when increasing the stringency to a 75% bootstrap-value (M1 consensus analysis; see Methods). 

**Figure 2:**
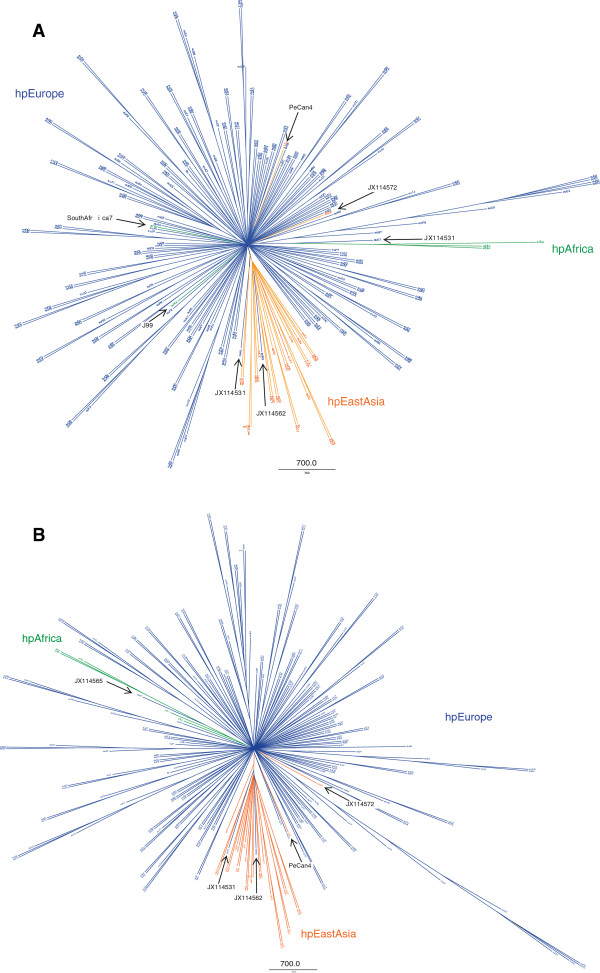
**Phylogenetic tree of* Helicobacter pylori pldA *sequences.** The * pldA * sequences were biogeographically classified: blue represents European strains, orange indicates hpEastAsian isolates, and green denotes African strains (hpAfrica). The outliers are identified by black arrows (see Discussion for more information). Additional file 1: Table S2 contain label with corresponding GenBank Accession ID. Shown are radial consensus trees of 246 * pldA * sequences based on 1000 maximum likelihood bootstrap replicates analyzed in PhyML and visualized in FigTree (see Methods for details). Trees were constructed using either the K80 + G + I model chosen by ModelTest (**A**) or the GTR + I + G model (**B**) as used to construct the reference tree (Figure 1).

The phylogenetic tree based upon the *pldA* gene sequences is depicted in Figure 2 (see Additional file 1: Table S2 for annotations). The majority of the Korean sequences clustered in the same clade. This cluster contained two isolates sampled in Norway that had an East Asian *cagA* EPIYA-ABD genotype and came from patients of East Asian origin. The four Amerindian strains and five East Asian sequences from the 19 genome sequences were also found in this cluster. One of the samples isolated in Norway was from a patient of African origin and clustered with the four African sequences. The *vacA* genotype of this sample was s1b, the genotype that is most common among the African, Spanish, and South American populations [21]. This *pldA* tree was unrooted and consisted of two main clusters, the East Asian cluster and the smaller African groups, nested within the vast majority of European sequences. The two African *pldA* sequences from the J99 and SouthAfrica7 genomes were found among the European sequences, as observed in the reference tree. Only three of the African strains formed a clade with 75% bootstrap analysis (in M1 consensus tree; data not shown). 

The two *pldA* trees constructed using different models were compared in TOPD/FMTS using split distances. The average split distance was 0.58, which indicated that the two trees were neither identical (split difference = 0) nor completely different (1). A random split distance was calculated to analyze whether the split distances were significantly different. Because the random split distance resulted in a value close to 1 (0.999885, to be exact), our observations were probably not due to chance.

### Horizontal gene transfer analysis of *pldA* and OMPLA sequences

The average GC content of the 19 *pldA* gene sequences was 40.18 ± 0.35%, while the average GC content of the corresponding 19 whole-genome sequences was 38.98 ± 0.21%, a significant difference (*P* ≈ 10^-12^). The *pldA* mean GC content was greater than 1.5 standard deviations from the GC genomic mean, suggesting horizontal transfer. We further assessed whether the codon bias found in the *pldA* gene sequences could be due to biological or random effects. The codon adaptation index (CAI) was estimated by CAIcal [22] to be 0.77, while the eCAI estimate was 0.75 (with p <0.01; 99% probability for 99% of the population). This yields a CAI/eCAI ratio of 1.03; a CAI value higher than the expected eCAI value indicates codon bias.

**Figure 3:**
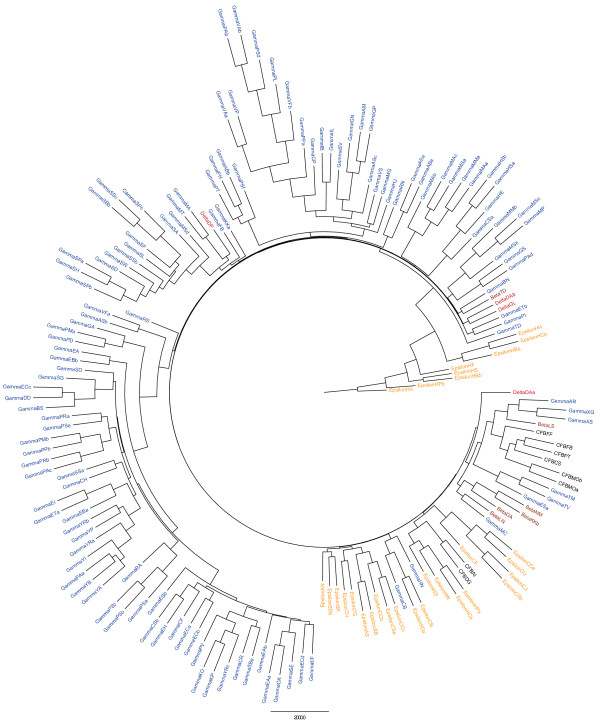
**Phylogenetic tree of Proteobacteria OMPLA sequences.** Majority-rule consensus tree of OMPLA sequences representing 171 species of gamma proteobacteria (blue), beta proteobacteria (brown), epsilon proteobacteria (orange), delta proteobacteria (red), and Bacteroidetes/Chlorobi (CFB; black). See Additional file 2: Table S3 for species labels used.

**Figure 4:**
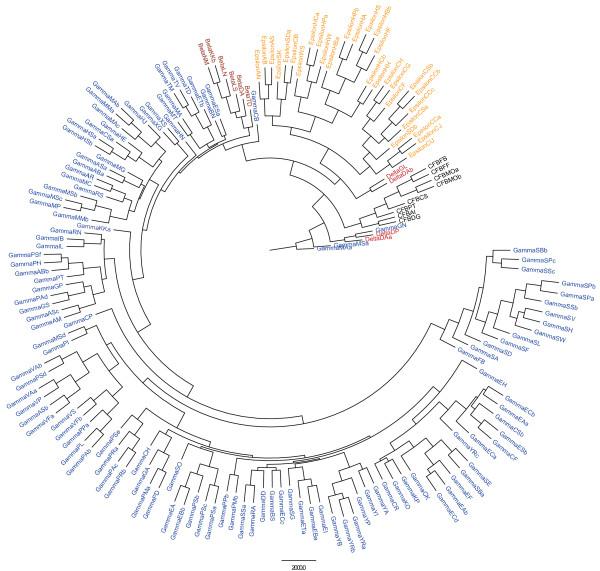
**Phylogenetic tree of Proteobacteria AtpA sequences.** Maximum likelihood majority-rule consensus tree of AtpA sequences derived from 169 species of gamma proteobacteria (blue), beta proteobacteria (brown), epsilon proteobacteria (orange), delta proteobacteria (red), and Bacteroidetes/Chlorobi (CFB; black). See Additional file 2: Table S3 for species labels used.

We collected 958 OMPLA sequences (listed in the Additional file 2: Table S3), of which 170 different species had pairwise sequence identities to *H. pylori* between 15% and 90%. The vast majority of the protein sequences used in this study were from proteobacteria, with gamma proteobacteria accounting for nearly 72%. In addition to proteobacteria, eight Bacteroidetes/Chlorobi (CFB) species were present. The average length of the OMPLA protein sequences was 320 amino acids (range 247–393), resulting in 79 residues in the final alignment. The phylogenetic tree of OMPLA is shown in Figure 3. The AtpA reference sequences had an average of 511 residues (range 499–548), and the final alignment contained 445 residues. The phylogenetic tree of AtpA is shown in Figure 4. Two Enterobacteriaceae species, *Proteus vulgaris* and *Pantoea agglomerans* (GammaPV and GammaPAa in Figure 3), see Additional file 3: Table S1 for the annotations used) were only found in the OMPLA dataset. The reference tree displays three distinct clusters of CFB, gamma, epsilon, and beta proteobacteria. However, the four delta sequences occurred in two separate clusters in both the reference and OMPLA trees. Two of them were sister to the epsilon sequences, as expected because they belong to the Epsilon/Delta subdivision within Proteobacteria. The main difference between the AtpA and OMPLA trees was that in the OMPLAtree the epsilon proteobacteria cluster was separated by multiple gamma clades. *Helicobacter acinonychis* and *H. pylori* were the two most distant sequences among all of the species in the OMPLA tree with a very strong bootstrap value (see Additional file 4). Sister to these two species were the remaining six *Helicobacter* spp., divided into two subclusters. The division of the epsilon group was also found using a 75% bootstrap support in the M1 consensus analysis) (see Additional file 5: Figure S2 and Additional file 6: Figure S3), indicating a strong branch that separates the *Helicobacter* sequences from the rest of the epsilon group. The largest cluster in the OMPLA phylogenetic tree consisted of about 50 gamma species. The remaining gamma sequences were found in closely-related subclusters. Some gamma proteobacteria were also related to either the epsilon, beta, or CFB subclusters.

### Adaptive molecular evolution in *pldA* sequences

The SWAAP analysis resulted in an average K_a_/K_s_ ratio of 0.076 ± 0.035, indicating a protein under strong purifying selection. The codon-based *Z*-test bootstrap analysis confirmed that a vast majority (98.86%) of the nucleotide sequences had a high probability (*p <* 0.01) of being under purifying selection.

Table 1 depicts the results of the test for positive selection in PAML. The two models that allowed positive selection, M2 and M8, fit our data better than the models, M1 and M7, that did not. The LRT showed that the M8 model best fit these data. This model estimated that fourteen sites (4.63%) were under positive selection (Table 2), with ω = 1.55 and 85.83% were under purifying selection, with ω < 0.2. The M2 model estimated that 92.12% of the sites were under purifying selection, while 1.46% was positively selected. PAML estimated κ ≈ 4 for M2 and M8.

**Table 1:** Likelihood ratio test for model selection

**Model**	**lnL**	**LRT**	**χ**^**2**^** distribution**
M1	−12515.96	47.04	>9 with 2 d.o.f. P < 0.01
M2	−12492.44		
M7	−12521.64	83.94	>9 with 2 d.o.f. P < 0.01
M8	−12479.67		

Nested models with and without positive selection (M1 vs. M2 and M7 vs. M8) were compared in PAML. The χ^2^ distribution column shows the minimum likelihood ratio (=2ΔlnL) necessary for the more complex of two models to be significantly better (p < 0.01).

**Table 2:** **Positively-selected sites in*pldA*of*Helicobacter pylori***

**Site**	**Residue**	**Probability ω >1**	**Posterior probability**
5	W	0.955*	1.48 ± 0.20
6	L	0.996**	1.52 ± 0.15
21	S	0.830	1.37 ± 0.33
27	I	1.000**	1.52 ± 0.14
34	R	0.576	1.15 ± 0.42
40	I	0.999**	1.52 ± 0.14
50	A	0.989*	1.51 ± 0.15
59	P	0.858	1.39 ± 0.29
137	D	1.000**	1.52 ± 0.14
144	D	0.760	1.32 ± 0.33
153	M	1.000**	1.52 ± 0.14
209	P	0.851	1.39 ± 0.31
211	G	0.836	1.38 ± 0.30
278	V	0.962*	1.48 ± 0.15

PAML predicted that 14 sites were under positive selection (ω >1) using Bayes empirical Bayes analysis for the M8 model. One asterisk (*) signifies a probability >95% that ω >1, while two asterisks (**) signify a probability greater than 99%. The best ancestral reconstruction is indicated by the highest value in the final posterior probability column.

## Discussion

Brok *et al.* compared OMPLA protein orthologs from eleven different species and concluded that OMPLA contained 30 highly-conserved residues. The fact that OMPLA is present in a wide range of species, including *H. pylori*, and that the sequence is conserved across those species, strongly indicates that its physiological role is significant [23]. This study aimed to better understand the significance of *pldA,* the gene coding for OMPLA, in *H. pylori*; an important gut bacterium in humans.

The *H. pylori pldA* gene had a low degree of variability and, thus, a conserved OMPLA protein sequence alignment. Housekeeping genes are essential for bacterial survival, and are thus highly conserved. The seven HK genes, *atpA*, *efp*, *ppa*, *tphC*, *ureI*, *trpC,* and *mutY,* and the *pldA* gene are among the core genes that are found in all *H. pylori* genomes sequenced to date [10]. The average sequence identity was higher in *pldA* and the molecular distance was lower than in the HK genes. The present study focused on analyzing *pldA* gene sequences that code for functional OMPLA proteins. In previous studies, we showed that most clinical isolates contain these coding *pldA*ON sequences [13]. In this study, we included 155 isolates from a Norwegian population used in the Sørreisa study [24]. Most (97.5%) of these isolates showed an ON phase variant, indicating that the gene encodes a functional OMPLA protein in most individuals. The homopolymeric tract induces a shift between a functional and a truncated protein by enabling a frameshift mutation. Wernegreen *et al.* postulated that selection will purge nucleotide changes that could interrupt the slippery tract, to maintain otherwise volatile sequences [25].

Why the *pldA* gene in *H. pylori* contains a homopolymeric tract is an enigma, and we explored whether its existence could be part of a gene deletion process or perhaps a mechanism needed to prevent activation in certain environments. The homopolymeric tract corresponded to residues 226–228 in the translated OMPLA protein. Residue 278 was the most downstream site that was predicted to be under positive selection in this protein. The remaining twenty percent of the protein (after residue number 279) is under purifying selection, indicating functional constraints and implying that the protein is important to bacterial survival.

Genes under purifying selection are often involved in host-pathogen interactions. For example, purifying selection in orthopoxvirus is probably caused by host defense mechanisms [26]. However, pathogens must also evolve novel residues to evade the host immune system, resulting in positive selection on some residues [27]. Such positive selection has been shown in the flagellum-coding gene *flA*, which is involved in adhesion in *Aeromonas*; nearly the entire protein was under purifying selection, while 17 residues were subject to positive selection [28]. Our analyses demonstrated purifying selection in most of the *pldA* sequence, while the remaining residues were predicted to be under positive selection. The positively-selected sites were scattered throughout the OMPLA protein. Petersen *et al*. concluded that positively-selected sites are exclusively located in the loops of outer membrane proteins [27]. In Rickettsiaceae, positively-selected sites were important for host-parasite interactions and were located at the exterior of the proteins [29]. The *E. coli* OMPLA structure had a beta-barrel transmembrane conformation [30]. Thus, one might reasonably assume that its positively-selected sites are also within surface-exposed regions. The N-terminal end of the protein contained four positively-selected sites (two with *p* ≥ 99), but they are most likely a signal sequence and not part of the mature protein.

Bacterial survival and persistence in the gastric mucosa requires adapting to an environment with constant fluctuating pH. *Helicobacter pylori* adjusts to pH fluctuations in the stomach by regulating the expressions of niche-adapted genes such as urease and ferric uptake regulator (Fur), which protect against acidity by producing ammonium and modulating the expression of many genes under acidic conditions, respectively. Both genes are required for survival under acidic conditions. Fur mutants do not colonize well and are probably killed by environmental conditions in regions other than the final colonization sites, like in the mucus layer. The exact mechanism still remains unclear [31]. Because the *pldA* gene is required for growth at low pH [32] and active OMPLA protein is important for survival in acidic environments [33], the gene may be part of the acidic environment niche-adapted mechanism described. *Helicobacter pylori* OMPLA is an outer-membrane protein that is exposed to the continuously changing environment of the host, so its interactions should be optimized. This could cause some of the residues to be under positive selection pressure while the rest of the protein is conserved and is typically observed in proteins that are in the process of adapting to environmental changes [34].

*Helicobacter pylori* has demonstrated geographical clustering of its HK, virulence, and outer membrane protein genes in phylogenetic studies [11,12,35-38]. Because many genes with biogeographic patterns are highly conserved, we were interested in determining whether *pldA* gene sequences showed such partitioning. As a point of reference, we constructed a phylogenetic tree with the same sequences used by Falush *et al*. [11]. We found biogeographic patterns in both the reference HK and *pldA* gene trees; however, bootstrap values in both trees, indicates relatively weak support for the biogeographic clades perhaps due to the high sequence identity found in both alignments. The strongest clade found in the *pldA* tree (with >75% bootstrap in the M1 consensus analysis; see Method section) contained three out of the four African H. *pylori*. However, one of the African isolates in the original analysis was not found in this clade. Thus, the African cluster could be due to the fact that the data were taken from same patient over many years [39].The HK reference tree contained sequences from around the world (using the Falush dataset and *H. pylori* genomes). The majority of the Amerindian samples clustered in the East Asian cluster, as reported for other genes [11,12,37]. However, although SJM180 is from a native American Peruvian isolate, it clustered with the European isolates, as described by Manjulata *et al.*[40]. The two samples in the East Asian subcluster were of East Asian origin and had an East Asian CagA genotype. The majority (86%) of the East Asian *pldA* sequences contained two mutations (residues K168E and E176K). In future work, we would like to assess whether and how these two mutations influence OMPLA structure and function.

The phylogenetic trees were constructed to analyze the biogeography of the *pldA* sequences. In the process, we discovered that the best-fit substitution model for the *pldA* sequences was different from that of the HK genes. This indicates that the genes have not evolved under the same conditions and could be explained by HGT of the *pldA* gene. The K80 algorithm adjusts for transition to transversion (ts/tv) bias which was also confirmed with a high ts/tv rate ratio (κ ~ 4) in the *pldA* dataset. We constructed two phylogenetic *pldA* trees, using the two models selected for reference and *pldA*, to determine how the model would affect the geographical clustering. This would give insight into how *pldA* sequence evolution compares to that of the housekeeping genes. The HK reference genes represents the core genome diversity within *H. pylori* as they are scattered around the genome, flanked by conserved genes not expected to be under any immune selection [11].The two trees were found to be quite different, with a split distance ratio of 0.58. Our findings were most likely due to biological effects rather than random bias. Interestingly, the only biogeographical difference observed between the two models was in the placement of the American J99 isolate, which had African characteristics [11]. This sequence was found in the European cluster in the *pldA* K80 tree, while it clustered with the other African sequences in both the HK and *pldA* GTR trees. These analyses could indicate that the genes have co-evolved along different phylogenetic lines for a long time and that a possible HGT event involving *pldA* may have occurred relatively early in the evolution.

Our hypothesis of HGT was confirmed through both intra- and inter-species evolutionary analyses. Multiple analyses can infer HGT, including phylogenetic analysis of orthologs and estimates of codon bias and GC content. Our results indicated an ancient transfer, because the *pldA* tree had a similar biogeographical pattern to that of the reference tree. The OMPLA protein is mainly found in gamma proteobacteria. Horizontal gene transfer has been observed in, *e.g.*, the CagPAI region, which has a lower GC content than the rest of the *H. pylori* genome [41]. The current study demonstrated a possible HGT event through the analysis of phylogeny, GC content, and codon bias. The GC content of the *pldA* sequences was slightly but significantly elevated compared to the rest of the *H. pylori* genome, and the difference was well above the accepted mean deviation threshold [42,43]. Although the *H. pylori* genomes as a whole lacked codon bias [44], further analysis was needed to ensure that the *pldA* gene was an exception to this conclusion. The CAI confirmed that the observed codon bias was most likely due to biological effects rather than artifact. Thus, the codon bias in the *pldA* gene suggested horizontal transfer [22]. Further confirmation for HGT was found in the phylogenetic analysis comparing OMPLA and AtpA sequences in which *Helicobacter* differed from the other epsilon proteobacteria. In particular, *H. pylori* and *H. acinonychis* were the two most divergent species in the OMPLA phylogenetic tree. These results were validated using an approximate likelihood ratio test in PhyML [45]. The phylogenetic tree of OMPLA conflicts with that of AtpA, indicating multiple HGT events. The species found outside of their expected clusters might have adapted quickly to environmental changes as a result of HGT events, which accelerate the rate of adaption [46]. This is illustrated in the epsilon cluster; three of the four non-epsilon bacteria in that clade colonize humans either as pathogenic bacteria or as part of the intestinal microbiota (see Figure 3 and Additional file 2: Table S3 for details).

## Conclusions

The *pldA* gene in *Helicobacter pylori* has high nucleotide sequence identity due to purifying selection at the vast majority of residues. The result is a conserved *H. pylori* protein that likely has an evolutionarily stable function, although some probable interaction sites are subject to positive selection. Although HGT was detected by codon bias, GC content, and phylogenetic analysis, the biogeography of the *pldA* sequences indicated that the transfer was ancient. The protein structure of *H. pylori* OMPLA will yield a better understanding of the positively selected sites, which may be surface-exposed regions. Our analyses indicated that *pldA* may be a niche-adapted protein; it was horizontally acquired, is highly conserved, but positive selection occurs at sites needed for possible pathogenic interactions.

## Methods

### *Helicobacter pylori* sample collection and *pldA* sequencing

The *pldA* gene of 227 *H. pylori* isolates was sequenced. The samples included 207 Norwegian and 20 Korean isolates. The Norwegian samples consisted of a total of 155 isolates from the Sørreisa study [24] and 52 isolates collected from four hospitals in the Oslo region. Among these isolates, 40 had been previously described [33]. The Oslo isolates included samples with known foreign origins; four isolates with Indo-European origins, two with Asian origins, and one with an African origin.

DNA was isolated using BioRobot M48 and MagAttract DNA Mini M48 Kit (Qiagen Inc., Valencia, CA, USA). The *pldA* gene, including short parts of the up- and downstream genes, was amplified by polymerase chain reaction (PCR) with forward primer HP498/499-F (5’- ttatcgcgcctgtagtga -3’) and reverse primer HP499/500-R (5’- tatgatcgctggcatgga -3’) at an annealing temperature of 57°C. The 1068 base pair (bp) *pldA*-gene was sequenced using the ABI BigDye Terminators v 1.1 Cycle Sequencing Kit (Applied Biosystems, Foster City, CA, USA) with the PCR primers and the internal sequencing primers HP498/499-R (5’-ggttgatattggggtggta-3’), PLA-F (5’-tgtccaattcttggtatctc-3’), PLA-R (5’-atgcgataggtatagcctaag-3’) and HP499/500-F (5’-tatgatcgctggcatgga-3’). The sequencing products were analyzed with an ABI PRISM 3130 Genetic Analyzer (Applied Biosystems) and the sequences were aligned using Sequencher software (Gene Codes Corporation, Ann Arbor, MI, USA). Finally, the DNA sequences were translated to complete protein sequences using RevTrans [47].

In addition to the 207 sequences collected in Norway that were included in this study, three additional isolates were sequenced and excluded because they coded for truncated proteins.

### *CagA* EPIYA genotyping

To discriminate the East Asian from the European isolates, the CagA genotype was determined in the 20 Korean samples and 50 of the Norwegian ones. Amplification and sequencing of the 3’ region of the *cagA* gene was performed as described by Yamaoka *et al.*[48].

### Amplification of *vacA*

To confirm the African origin of one of the Norwegian samples, PCR amplification of the *vacA* signal sequence and mid-region was performed as described by Atherton *et al*. [49].

### Biogeographic analysis

#### Reference phylogenetic tree

A reference phylogenetic tree was constructed using concatenated HK genes (*atpA*, *efp*, *ppa*, *tphC*, *ureI*, *trpC,* and *mutY*) collected from the *H. pylori* Multi Locus Sequence Typing (MLST) database http://pubmlst.org/helicobacter/ as described by Falush *et al.*[11]. In addition, 19 of the 29 currently-sequenced *H. pylori* genomes (See Appendix 1 for further annotation) collected from the National Center for Biotechnology Information (NCBI) database http://www.ncbi.nlm.nih.gov and four Norwegian isolates, sequenced according to the *H. pylori* MLST protocol, were used in the reference tree construction. In total, 393 sequences were aligned using ClustalW [50], and regions with gaps were removed using BioEdit [51]. Model selection in MEGA5 [52] was used to determine the best fit model for maximum likelihood (ML) analysis. PhyML v3.0 [53] was used to generate 1000 ML bootstrap trees using the generalized time-reversible (GTR) model in which both the discrete gamma distribution (+G) with five rate categories and invariable sites (+I) were set to 0.61, as this was the model with the lowest Bayesian Information Criterion score.

A consensus tree was constructed with Phylip’s Consense package [54] and imported into FigTree v1.3.1 http://tree.bio.ed.ac.uk/software/figtree/ for further visualization. These resolved trees contain monophyletic groups not contradicting more frequent groups with a 50% default threshold (majority-rule). As a supplement, a strict analysis with a higher threshold was included where only groups occurring more than 75% are included.

#### *PldA* phylogenetic tree

The phylogenetic tree for *pldA* gene sequences was constructed using the same method as described for the reference tree. The *pldA* sequences were obtained through a Blast search of jhp_0451, limiting the search to *H. pylori* genome sequences. Only *pldA*ON sequences coding for the entire OMPLA protein were included in this study. In addition, 19 of the 29 currently-sequenced *H. pylori* genomes collected from the NCBI database were aligned with the *pldA* gene sequences from the 227 isolates described in the current study. Genomes containing *pldA* genes that coded for truncated proteins were excluded from analyses. Reversed sequences from the genomes were converted to a 5’–3’ reading frame using a reverse complement calculator http://www.bioinformatics.org/sms/rev_comp.html**]**. The *pldA* alignment was stripped of gaps in BioEdit [51] and imported into MEGA5 [52] for model selection as described above. The alignments were analyzed in PhyML [53] using 1000 bootstraps and the Kimura two-parameter (K80) model with the gamma distribution (five rate categories) and invariant sites set to 0.34 and 0.53, respectively; this model was found to be the best by MEGA5. A consensus tree was made in Phylip’s Consense package [54] and represented as an unrooted radial tree in FigTree. The *pldA* dataset was also analyzed using the same model (GTR + G + I) used for the reference tree. The two *pldA* trees generated using the GTR + G + I and K80 + G + I models were compared with the TOPD/FMTS software [55]. A random average split distance of 100 trees was also created to check if the differences observed were more likely to have been generated by chance.

### Comparison of *pldA* sequences with seven core housekeeping genes

The average pairwise nucleotide identity for *pldA* and concatenated HK sequences was calculated in BioEdit [51]. The average genetic distance was calculated with the default K80 algorithm in MEGA5 [53,56].

### Horizontal gene transfer analysis of *pldA* and OMPLA sequences

The DNA stability was determined by calculating the GC content of the *pldA* sequences using SWAAP 1.0.3 [57]. The GC content of the *pldA* sequences was compared to the overall GC content of the *H. pylori* genomes, and significant differences between these two groups were calculated using a two-tailed *t*-test (Excel 2003, Microsoft, Redmond, WA, USA). The Codon Adaptation Index (CAI) detects codon bias in a DNA sequence and indicates the possibility of HGT. CAIcal [22] was used to calculate the degree of codon bias and compare it to an estimated value from a reference set (eCAI).

The OMPLA protein sequences from 171 species were used for an intra-species phylogenetic analysis. Sequences were collected both from the KEGG database [58], using KEGG orthologs belonging to EC13.3.13, and, NCBI’s similar sequence option. Both NCBI Batch Entrez http://www.ncbi.nlm.nih.gov/sites/batchentrez and the Protein Information Resource (PIR) [59] were used to retrieve the protein sequences. Pairwise sequence identities were calculated for ClustalW aligned sequences in BioEdit [51]. Sequences with pairwise identities between 15-90% were kept, and the sequences (Appendix 1 lists all of the Protein IDs used) were re-aligned using the MAFFT web server http://www.genome.jp/tools/mafft/, where the auto-option chose the FFT-NS-i model (an iterative method) [60]. Jalview [61] displayed the minimum, maximum, and average number of residues in the alignment. Poorly-aligned and divergent regions were removed using Gblocks [62]. PhyML [53] was used to construct a phylogenetic tree using the default variables (including LG ( Le and Gascuel) method estimates of the gamma and invariable sites). The same procedure was performed on the ATP synthase subunit alpha (AtpA) reference sequences that were collected for the species in the OMPLA protein list by searching the protein NCBI database (See Appendix 1 for the Protein IDs used). The consensus tree of AtpA and OMPLA sequences were generated from the 1000 PhyML bootstrap trees using Phylip’s Consense package [54]. Results were visualized as circular trees using FigTree http://tree.bio.ed.ac.uk/software/figtree/.

### Detection of adaptive molecular evolution of *pldA* sequences

To study evolutionary divergence among the *pldA* sequences, the mean numbers of synonymous (K_s_) and nonsynonymous (K_a_) substitutions per site were estimated using the Nei and Gojobori method [63] in SWAAP [57]. The K_s_ value is the mean number of synonymous (silent) substitutions per site, while K_a_ represents the mean number of nonsynonymous substitutions per site (a change of amino acid is observed). The MEGA5 [52] codon-based *Z*-test for purifying selection was used to estimate the probability of rejecting strict neutrality (null hypothesis where K_a_ equals K_s_) in favor of the alternate hypothesis K_a_ < K_s_.

The PAML program [64] estimates the nonsynonymous/synonymous ratio, omega (ω), using maximum likelihood codon substitution models. In this study, four different models (M1, M2, M7, and M8) were used to estimate ω as described by Yang *et al.*[65]. These models are nested pairs in which one (M1 and M7) does not allow for positive selection, while the other (M2 and M8) includes an additional parameter to detect positively selected sites. The neutral model M1 assumes two classes of proteins, highly conserved codons (ω = 0) and neutral codons (ω = 1), and is nested within the M2 model, which has a third category for positive selection (ω > 1). The two most advanced models, M7 and M8, use a discrete ß distribution; M8 has an extra class of codons that allows positive detection (ω > 1) and simplifies to M7. The two pairs of nested models (M1 vs. M2 and M7 vs. M8) were compared using the likelihood ratio test (LRT) statistic, where 2ΔlnL equals 2*(lnL_1_ – lnL_0)_. The lnL_1_-value is the log-likelihood for the more advanced model and lnL_0_ is the log-likelihood for the simpler model. The 2ΔlnL value follows a χ^2^ distribution, where the degree of freedom is the difference in the number of parameters used in the two models. The identification of positive selected sites implemented in PAML uses Bayes empirical Bayes where the posterior probabilities of each codon was calculated from the site class of the M2 and M8 models; sampling errors have been accounted for through Bayesian prior [66,67]. A *pldA* tree generated in PhyML using the K80 model (the best fit as determined in MEGA5) was used in the PAML analysis. PAML also calculated possible transition (ts) to transversion (tv) bias (κ = ts/tv).

### Nucleotide sequence accession numbers

The nucleotide sequences analyzed in this study were deposited in the NCBI GenBank database under accession numbers JX114520 to JX114746.

## Appendix 1: Protein and gene annotation IDs

The 19 genomes used, and their *pldA* EMBL IDs, along with their expected *Helicobacter pylori* biogeographic traits are listed below:

· European traits: HPAG1, Lithuania75, P12, 52, 26695, SJM180, India7 [NCBI NC_008086.1, CP002334.1, NC_011498.1, CP001680.1, NC_000915.1, NC_014560.1, CP002331.1];

· African traits: J99, 2017, 2018, 908 and SouthAfrica7 [NCBI NC_000921.1, CP002571.1, CP002572.1, CP002184.1, CP002336.1, CP002337.1, ];East Asian traits: F16, F30, 35A, PeCan4, Shi470, 83 and Sat464 [NCBI AP011940.1, AP011941.1, CP002096.1, CP002074.1, NC_010698.2, CP002605.1, CP002071.1].

Genes that coded for truncated proteins (*pldA* OFF) were not included in this study.

The 169 AtpA sequences used in the HGT analysis

AtpA [NCBI: EHB93466.1, EEB65020.1, EGK01617.1, EAZ96951.1, EIA10014.1, EHO10730.1, EHQ42656.1, EAS72787.1, AAZ48838.1, ACV28038.1, EGK08739.1, EEG10159.1, EDM84731.1, EGC64000.1, AAZ98752.1, ACN14443.1, EAT15601.1, ADW17434.1, ACD96878.1, EFU68802.1, ADG93995.1, BAK73949.1, EDZ61621.1, EIB16597.1, EAT97454.1, EAU01020.1, ABK81906.1, EEV18591.1, ABS52242.1, ADN90332.1, EET80348.1, EHL90702.1, EFU71262.1, CAJ99396.1, EEO22948.1, CCF80240.1, EFR48376.1, EFR47618.1, CBY83548.1, AAP77024.1, EEQ62944.1, AAD08176.1, EFX42435.1, EEO26643.1, ABB44682.1, ACZ11550.1, ADR33423.1, CAE09651.1, CAL18176.1, EAW26695.1, AEB00215.1, EEY85631.1, EDX91133.1, CAQ80745.1, AEF05917.1, EAR22945.1, EHD23759.1, AAO91433.1, EHL85304.1, ACQ68874.1, YP_001451687.1, AAZ26667.1, CBG90709.1, ABE60630.1, ABU79194.1, ADN00765.1, CBJ48151.1, AEN67142.1, EDS93360.1, EFV38590.1, CAX62120.1, EFC54899.1, AEW75952.1, CAG77409.1, CAP78192.1, CAQ91467.1, GAB51972.1, ACR71021.1, EHQ52780.1, ABP62783.1, EFE21167.1, EGW54096.1, ADN77981.1, AEC17221.1, AEP31454.1, GAB56517.1, AEE25184.1, CBV44330.1, ABC33685.1, ACX97137.1, EHK61102.1, EGP19691.1, EAQ31531.1, AAV83453.1, EHS93248.1, AEK00623.1, EGL54277.1, ADP99760.1, EDM48519.1, ABM20945.1, EGE27602.1, EAW32658.1, EHJ04715.1, ADZ93414.1, AEF56544.1, EBA00697.1, EAQ64801.1, ABR73359.1, EDM65164.1, EEF79996.1, EAS66680.1, EEB44391.1, ABG42796.1, EEX50537.1, EGI73341.1, ABM05406.1, GAA05763.1, AET16617.1, EEI49869.1, EAS45491.1, EEG87182.1, EFE51392.1, EFB70640.1, EFM18673.1, ADU71268.1, EIB97664.1, EAR55051.1, EDU61485.1, GAA64110.1, EAR27048.1, AEX54272.1, GAB59628.1, EAR11223.1, ABM01849.1, CCC32467.1, AEG13513.1, ABE57027.1, CAR35257.1, ABI73872.1, BAE75687.1, ABZ78836.1, ABO25710.1, EFA14838.1, ABV89552.1, ACJ31773.1, ADV56630.1, EIC83933.1, ABV39090.1, EGM67869.1, BAJ04308.1, ACA89149.1, EGV28007.1, EGV18064.1, EGZ46719.1, EAS75526.1, EAS62862.1, AAW87061.1, EEX40605.1, EGF42098.1, EDL54805.1, EGD19228.1, ZP_09853641.1, EEP94770.1, EEQ08006.1, EEQ18999.1, YP_654074.1, EEQ03775.1, EEQ00089.1, EHM50189.1].

The 171 OMPLA sequences used in the HGT analysis

OMPLA: [NCBI EAZ99640.1, ADW17991.1, EHQ52957.1, EGL54504.1, EGK10785.1, EGV19191.1, CAQ79680.1, EEY87557.1, EEB64935.1, EHO08344.1, EGC65261.1, EIA07918.1, EAR22975.1, EGD17737.1, EHK60019.1, AAZ46833.1, AAZ96049.1, EGP20312.1, EHB92999.1, EDM47887.1, ZP_09857083.1, EHJ06187.1, EAS71795.1, EDM84432.1, ABM17560.1, GAB54415.1, AEP29176.1, EGK01773.1, CAL17552.1, EEF79803.1, ACN14146.1, ADR35309.1, EDX88885.1, EHQ44562.1, EET80219.1, ABB43297.1, AEF53991.1, ADP95974.1, AEE23125.1, ADZ90582.1, EAR10180.1, EAQ32639.1, CBV41928.1, EDL54875.1, ABR72196.1, EAQ63108.1, ACV26008.1, EAS65010.1, EGZ42951.1, EGV31023.1, ZP_01234806.1, GAA04467.1, EEG09398.1, EDZ63591.1, EAR56640.1, EGF41493.1, AAV83321.1, AEF05108.1, AEA97203.1, EAU01382.1, ACQ67963.1, CAD32066.1, EAS76085.1, ADG93813.1, ABM05176.1, EAZ96211.1, ABE58799.1, ABS52347.1, AAW86051.1, ABG40599.1, EDM67950.1, EEV17429.1, ADN76662.1, EHD19745.1, ABC27991.1, ADN00421.1, EFB72463.1, BAK72959.1, ABV35292.1, BAJ03481.1, GAB60703.1, ACA85081.1, EAR28662.1, EGI74195.1, EEB46686.1, GAA62323.1, EAT16431.1, EAS40470.1, ACJ30728.1, ACD97136.1, AEN66963.1, EAW30307.1, ABZ78078.1, EFE52140.1, EDU58126.1, EFC53577.1, ABO22543.1, ABV11329.1, ACX96270.1, EAW29496.1, EIC83527.1, ABV85988.1, ABM01096.1, BAE75613.1, CAR35328.1, EEP97888.1, EGM70992.1, CAA54224.1, EFA15011.1, ABU78936.1, AET16551.1, EFU69622.1, ABI73025.1, EGW55053.1, ACZ13275.1, EEQ18686.1, EEP94174.1, ABE54243.1, AEG10235.1, CAQ91143.1, EHL84474.1, CAX57751.1, 1FW2, ABP62630.1, EHM51878.1, GAB53576.1, EHS92439.1, CBG90636.1, EFV38511.1, EAT97941.1, CCC32538.1, CAA54223.1, EIB97812.1, EEG87253.1, CAE01133.1, ADV55550.1, EDS90253.1, EEX50977.1, EEQ03301.1, AAD03498.1, AEX54094.1, ABK82197.1, ACR67376.1, EEQ04956.1, EFM18818.1, EEI47649.1, ADU67494.1, ACV41773.1, CAA71915.1, EFE21458.1, AEC17546.1, CAE09192.1, CAJ99604.1, EEO25025.1, CCF79664.1, EES88872.1, EFR45804.1, CBY82368.1, AAP77450.1, EEQ63232.1, AAD07564.1, EFX41646.1, EEO25572.1] [SwissProt C9PFN8_VIBFU, D4ICJ7_ERWAE, D6DP51_ENTCL, E6LA24_CAMUP, Q0P8Q8_CAMJE, Q83E43_COXBU] [PRF 3020410HLP, 3117429CWR].

## Abbreviations

AtpA: ATPase F1 subunit alpha; Bp: Base pairs; CagPAI: Cag pathogenicity island; CAI: Codon adaptation index; CFB: Cytophaga-flavibacterium-bacteroides; eCAI: Estimated codon adaptation index; FUR: Ferric uptake regulator; GTR: Generalized time-reversible algorithm; HGT: Horizontal gene transfer; HK: Housekeeping genes; M2-M8: PAML models 2-8; LG: Le and gascuel substation matrix (2008); LRT: Likelihood ratio test; NCBI: National Center for Biotechnology Information; OMPLA: Outer membrane phospholipase A; PCR: Polymerase chain reaction; PAML: Phylogenetic analysis of maximum likelihood; ML: Maximum likelihood; MLST: Multi locus sequence typing; K80: Kimura-2-parameter algorithm; Ka: Synonymous-value (Dn); Ks: Nonsynonymous-value (Ds); PCR: Polymerase chain reaction; PIR: Protein information resource; T4SS: Type IV secretion system; Ts/Tv: Transition /transversion (κ).

## Competing interests

The authors declare no competing interests.

## Authors’ contributions

HSV planned the study design and performed all the bioinformatic analyses. YY made the Korean isolates available for this study and provided insightful comments with regard to outer membrane proteins of *H. pylori*. TT sequenced *pldA,* genotyped CagA from the Norwegian and Korean isolates and contributed throughout the process. GB supervised the study. All authors read and approved the final manuscript.

## Supplementary Material

Additional file 1 Table S2 *pldA* labeling. Lists the NCBI accession number with the corresponding labelling used in Figure 2a and b. (XLSX 14 kb) Click here for file

Additional file 2 Table S3 Proteobacteria labelling. This table contains the abbreviated Proteobacteria names found in Figures 3 and 4 with the corresponding full bacteria name. (XLSX 12 kb) Click here for file

Additional file 3 Table S1 Housekeeping labelling. This table lists the MLST ID or NCBI accession number of the 7 concatenated housekeeping genes used in the analysis depicted in Figure 1. Click here for file

Additional file 4 **Extended majority rule consensus tree (outfiles).** The outfiles that are the CONSENSE software results file from the phylogenetic trees from the phylogenetic analysis of housekeeping (Figure 1), *pldA* (Figure 2a and b), OMPLA (Figure 3) and AtpA (Figure 4). (RTF 405 kb) Click here for file

Additional file 5 Figure S2 Phylogenetic tree of Proteobacteria OMPLA sequences. Additional file 5 is a strict analysis of the OMPLA sequences found Figure 3. In this analysis, a higher threshold is used where only groups occurring more than 75% is included (M75). (PNG 1253 kb) Click here for file

Additional file 6 Figure S3 Phylogenetic tree of Proteobacteria AtpA sequences. Additional file 5 is a strict analysis (M75) of the OMPLA sequences found Figure 4. (PNG 903 kb) Click here for file

Additional file 7 Figure S1 Phylogenetic tree of H. pylori housekeeping sequences. Additional file 7 supplements Figure 1 with complete labelling. Click here for file

## References

[B1] Yoshiyama H Nakazawa T Unique mechanism of Helicobacter pylori for colonizing the gastric mucus Microbes Infect 2000 2 1 55 60 10.1016/S1286-4579(00)00285-9 10717541

[B2] Bergman M del Prete G van Kooyk Y Appelmelk B Helicobacter pylori phase variation, immune modulation and gastric autoimmunity Nat Rev Microbiol 2006 4 2 151 159 10.1038/nrmicro1344 16415930

[B3] Sipponen P Hyvärinen H Seppälä K Blaser M Review article: pathogenesis of the transformation from gastritis to malignancy Aliment Pharmacol Ther 1998 12 Suppl 1 61 71 9701004 10.1111/j.1365-2036.1998.00005.x

[B4] Israel D Peek RJ The role of persistence in Helicobacter pylori pathogenesis Curr Opin Gastroenterol 2006 22 1 3 7 10.1097/01.mog.0000194790.51714.f0 16319669

[B5] Kusters J van Vliet A Kuipers E Pathogenesis of Helicobacter pylori infection Clin Microbiol Rev 2006 19 3 449 449 10.1128/CMR.00054-05 16847081 PMC1539101

[B6] Covacci A Rappuoli R Helicobacter pylori: molecular evolution of a bacterial quasi-species Curr Opin Microbiol 1998 1 1 96 102 10.1016/S1369-5274(98)80148-3 10066468

[B7] Kuipers E Israel D Kusters J Gerrits M Weel J van Der Ende A van Der Hulst R Wirth H Höök-Nikanne J Thompson S Quasispecies development of Helicobacter pylori observed in paired isolates obtained years apart from the same host J Infect Dis 2000 181 1 273 282 10.1086/315173 10608776 PMC2766531

[B8] Nedenskov-Sørensen P Bukholm G Bøvre K Natural competence for genetic transformation in Campylobacter pylori J Infect Dis 1990 161 2 365 366 10.1093/infdis/161.2.365 2299221

[B9] Smeets L Kusters J Natural transformation in Helicobacter pylori: DNA transport in an unexpected way Trends Microbiol 2002 10 4 159 162 10.1016/S0966-842X(02)02314-4 11912014

[B10] McClain MS Shaffer CL Israel DA Peek RMJ Cover TL Genome sequence analysis of Helicobacter pylori strains associated with gastric ulceration and gastric cancer BMC Genomics 2009 10 3 10.1186/1471-2164-10-3 19123947 PMC2627912

[B11] Falush D Wirth T Linz B Pritchard J Stephens M Kidd M Blaser M Graham D Vacher S Perez-Perez G Traces of human migrations in Helicobacter pylori populations Science 2003 299 5612 1582 1585 10.1126/science.1080857 12624269

[B12] Linz B Balloux F Moodley Y Manica A Liu H Roumagnac P Falush D Stamer C Prugnolle F van der Merwe S An African origin for the intimate association between humans and Helicobacter pylori Nature 2007 445 7130 915 918 10.1038/nature05562 17287725 PMC1847463

[B13] Yamaoka Y Kato M Asaka M Geographic differences in gastric cancer incidence can be explained by differences between Helicobacter pylori strains Intern Med 2008 47 12 1077 1083 10.2169/internalmedicine.47.0975 18552463 PMC3732488

[B14] Zhong Q Shao S Cui L Mu R Ju X Dong S Type IV secretion system in Helicobacter pylori: a new insight into pathogenicity Chin Med J (Engl) 2007 120 23 2138 2142 18167190

[B15] Olbermann P Josenhans C Moodley Y Uhr M Stamer C Vauterin M Suerbaum S Achtman M Linz B A global overview of the genetic and functional diversity in the Helicobacter pylori cag pathogenicity island PLoS Genet 2010 6 8 e1001069 10.1371/journal.pgen.1001069 20808891 PMC2924317

[B16] Dorrell N Martino M Stabler R Ward S Zhang Z McColm A Farthing M Wren B Characterization of Helicobacter pylori PldA, a phospholipase with a role in colonization of the gastric mucosa Gastroenterology 1999 117 5 1098 1104 10.1016/S0016-5085(99)70394-X 10535872

[B17] Ziprin R Young C Byrd J Stanker L Hume M Gray S Kim B Konkel M Role of Campylobacter jejuni potential virulence genes in cecal colonization Avian Dis 2001 45 3 549 557 10.2307/1592894 11569726

[B18] Tannaes T Bukholm I Bukholm G High relative content of lysophospholipids of Helicobacter pylori mediates increased risk for ulcer disease FEMS Immunol Med Microbiol 2005 44 1 17 23 10.1016/j.femsim.2004.10.003 15780574

[B19] Kawai M Furuta Y Yahara K Tsuru T Oshima K Handa N Takahashi N Yoshida M Azuma T Hattori M Evolution in an oncogenic bacterial species with extreme genome plasticity: Helicobacter pylori East Asian genomes BMC Microbiol 2011 16 11 104 21575176 10.1186/1471-2180-11-104 PMC3120642

[B20] de Sablet T Piazuelo M Shaffer C Schneider B Asim M Chaturvedi R LE B Sicinschi L Delgado A Mera R Phylogeographic origin of Helicobacter pylori is a determinant of gastric cancer risk Gut 2011 60 9 1189 1195 10.1136/gut.2010.234468 21357593 PMC3133872

[B21] Nagiyev T Yula E Abayli B Koksal F Prevalence and genotypes of Helicobacter pylori in gastric biopsy specimens from patients with gastroduodenal pathologies in the Cukurova region of Turkey J Clin Microbiol 2009 47 12 4150 4153 10.1128/JCM.00605-09 19846654 PMC2786667

[B22] Puigbò P Bravo I Garcia-Vallve S CAIcal: a combined set of tools to assess codon usage adaptation Biol Direct 2008 3 38 10.1186/1745-6150-3-38 18796141 PMC2553769

[B23] Brok R Boots A Dekker N Verheij H Tommassen J Sequence comparison of outer membrane phospholipases A: implications for structure and for the catalytic mechanism Res Microbiol 1998 149 10 703 710 10.1016/S0923-2508(99)80017-5 9921577

[B24] Bernersen B Johnsen R Bostad L Straume B Sommer A Burhol P Is Helicobacter pylori the cause of dyspepsia? BMJ 1992 304 6837 1276 1279 10.1136/bmj.304.6837.1276 1606428 PMC1881886

[B25] Wernegreen J Kauppinen S Degnan P Slip into something more functional: selection maintains ancient frameshifts in homopolymeric sequences Mol Biol Evol 2010 27 4 833 839 10.1093/molbev/msp290 19955479 PMC2877537

[B26] Esteban D Hutchinson A Genes in the terminal regions of orthopoxvirus genomes experience adaptive molecular evolution BMC Genomics 2011 12 261 10.1186/1471-2164-12-261 21605412 PMC3123329

[B27] Petersen L Bollback J Dimmic M Hubisz M Nielsen R Genes under positive selection in Escherichia coli Genome Res 2007 17 9 1336 1343 10.1101/gr.6254707 17675366 PMC1950902

[B28] Farfán M Miñana-Galbis D Fusté M Lorén JG Divergent evolution and purifying selection of the flaA gene sequences in Aeromonas Biol Direct 2009 4 23 10.1186/1745-6150-4-23 19622168 PMC2724415

[B29] Jiggins F Hurst G Yang Z Host-symbiont conflicts: positive selection on an outer membrane protein of parasitic but not mutualistic Rickettsiaceae Mol Biol Evol 2002 19 8 1341 1349 10.1093/oxfordjournals.molbev.a004195 12140246

[B30] Snijder H Ubarretxena-Belandia I Blaauw M Kalk K Verheij H Egmond M Dekker N Dijkstra B Structural evidence for dimerization-regulated activation of an integral membrane phospholipase Nature 1999 401 6754 717 721 10.1038/44890 10537112

[B31] Gancz H Censini S Merrell D Iron and pH homeostasis intersect at the level of Fur regulation in the gastric pathogen Helicobacter pylori Infect Immun. Infect Immun 2006 74 1 602 614 10.1128/IAI.74.1.602-614.2006 PMC1346641 16369017

[B32] Reid A Pandey R Palyada K Whitworth L E D, Stintzi A: Identification of Campylobacter jejuni genes contributing to acid adaptation by transcriptional profiling and genome-wide mutagenesis Appl Environ Microbiol 2008 74 5 1598 1612 10.1128/AEM.01508-07 18192408 PMC2258640

[B33] Tannaes T Dekker N Bukholm G Bijlsma J Appelmelk B Phase variation in the Helicobacter pylori phospholipase A gene and its role in acid adaptation Infect Immun 2001 69 12 7334 7340 10.1128/IAI.69.12.7334-7340.2001 11705905 PMC98819

[B34] Padhi A Verghese B Otta S Detecting the form of selection in the outer membrane protein C of Enterobacter aerogenes strains and Salmonella species Microbiol Res 2009 164 3 282 289 10.1016/j.micres.2006.12.002 17418551

[B35] Oleastro M Cordeiro R Ménard A Yamaoka Y Queiroz D Mégraud F Monteiro L Allelic diversity and phylogeny of homB, a novel co-virulence marker of Helicobacter pylori BMC Microbiol 2009 9 248 10.1186/1471-2180-9-248 19954539 PMC2795765

[B36] Pride D Blaser M Concerted evolution between duplicated genetic elements in Helicobacter pylori J Mol Biol 2002 316 3 629 642 10.1006/jmbi.2001.5311 11866522

[B37] Cao P Lee K Blaser M Cover T Analysis of hopQ alleles in East Asian and Western strains of Helicobacter pylori FEMS Microbiol Lett 2005 251 1 37 43 10.1016/j.femsle.2005.07.023 16102915

[B38] Yamaoka Y Orito E Mizokami M Gutierrez O Saitou N Kodama T Osato M Kim J Ramirez F Mahachai V Helicobacter pylori in North and South America before Columbus FEBS Lett 2002 517 1–3 180 184 12062433 10.1016/s0014-5793(02)02617-0

[B39] Avasthi T Devi S Taylor T Kumar N Baddam R Kondo S Suzuki Y Lamouliatte H Mégraud F Ahmed N Genomes of two chronological isolates (Helicobacter pylori 2017 and 2018) of the West African Helicobacter pylori strain 908 obtained from a single patient J Bacteriol 2011 193 13 3385 3386 10.1128/JB.05006-11 21515762 PMC3133283

[B40] Devi S Ahmed I Khan A Rahman S Alvi A Sechi L Ahmed N Genomes of Helicobacter pylori from native Peruvians suggest admixture of ancestral and modern lineages and reveal a western type cag-pathogenicity island BMC Genomics 2006 27 7 191 16872520 10.1186/1471-2164-7-191 PMC1553449

[B41] Salaün L Saunders N Population-associated differences between the phase variable LPS biosynthetic genes of Helicobacter pylori BMC Microbiol 2006 6 79 10.1186/1471-2180-6-79 16981984 PMC1599737

[B42] Penn K Jenkins C Nett M Udwary D Gontang E McGlinchey R Foster B Lapidus A Podell S Allen E Genomic islands link secondary metabolism to functional adaptation in marine Actinobacteria ISME J 2009 3 10 1193 1203 10.1038/ismej.2009.58 19474814 PMC2749086

[B43] Raiford D Krane D Doom T Raymer M Automated isolation of translational efficiency bias that resists the confounding effect of GC(AT)-content IEEE/ACM Trans Comput Biol Bioinform 2010 7 2 238 20431144 10.1109/TCBB.2008.65

[B44] Lafay B Atherton J Sharp P Absence of translationally selected synonymous codon usage bias in Helicobacter pylori Microbiology 2000 146 Pt 4 851 860 10784043 10.1099/00221287-146-4-851

[B45] Anisimova M Gascuel O Approximate likelihood ratio test for branchs: a fast, accurate and powerful alternative Syst Biol 2006 55 4 539 552 10.1080/10635150600755453 16785212

[B46] Haggerty L Martin F Fitzpatrick D McInerney J Gene and genome trees conflict at many levels Phil Trans R Soc B 2009 364 1527 2209 2219 10.1098/rstb.2009.0042 19571241 PMC2873008

[B47] Wernersson R Pedersen A RevTrans: multiple alignment of coding DNA from aligned amino acid sequences Nucleic Acids Res 2003 31 13 3537 3539 10.1093/nar/gkg609 12824361 PMC169015

[B48] Yamaoka Y Kodama T Kashima K Graham D Sepulveda A Variants of the 3' Region of the cagA gene in Helicobacter pylori isolates from patients with different H. pylori-associated diseases J Clin Microbiol 1998 36 8 2258 2263 9666002 10.1128/jcm.36.8.2258-2263.1998 PMC105028

[B49] Atherton J Cao P Peek RJ Tummuru M Blaser M Cover T Mosaicism in vacuolating cytotoxin alleles of Helicobacter pylori. Association of specific vacA types with cytotoxin production and peptic ulceration J Biol Chem 1995 270 30 17771 17777 10.1074/jbc.270.30.17771 7629077

[B50] Larkin M Blackshields G Brown N Chenna RMP McWilliam H Valentin F Wallace IM Wilm A Lopez R Thompson JD Gibson TJ Higgins DG ClustalW and ClustalX version 2 Bioinformatics 2007 23 21 2947 2948 10.1093/bioinformatics/btm404 17846036

[B51] Hall T BioEdit: a user-friendly biological sequence alignment editor and analysis program for Windows 95/98/NT Nucl Acids Symp Ser 1999 41 95 98

[B52] Tamura K Peterson D Peterson N Stecher G Nei M Kumar S MEGA5: Molecular Evolutionary Genetics Analysis using Maximum Likelihood, Evolutionary Distance, and Maximum Parsimony Methods Mol Biol Evol 2011 28 2731 2739 10.1093/molbev/msr121 21546353 PMC3203626

[B53] Guindon S Dufayard J Lefort V Anisimova M Hordijk W Gascuel O New algorithms and methods to estimate maximum-likelihood phylogenies: assessing the performance of PhyML 3.0 Syst Biol 2010 59 3 307 321 10.1093/sysbio/syq010 20525638

[B54] Felsenstein J PHYLIP (Phylogeny Inference Package) version 3.6 Distributed by the author 2004 Department of Genome Sciences, University of Washington, Seattle

[B55] Puigbo P Garcia-Vallve S McInerney J TOPD/FMTS: a new software to compare phylogenetic trees Bioinformatics 2007 23 1556 1558 10.1093/bioinformatics/btm135 17459965

[B56] Kimura M A simple method for estimating evolutionary rates of base substitutions through comparative studies of nucleotide sequences J Mol Evol 1980 16 2 111 120 10.1007/BF01731581 7463489

[B57] Pride D SWAAP Version 1.0 -Sliding windows alignment analysis program: a tool for analyzing patterns of substitutions and similarity in multiple alignments Distributed by the author 2000

[B58] Ogata H Goto S Sato K Fujibuchi W Bono H Kanehisa M KEGG: Kyoto Encyclopedia of Genes and Genomes Nucleic Acids Res 1999 27 1 29 34 10.1093/nar/27.1.29 9847135 PMC148090

[B59] Wu CH Huang H Arminski L Castro-Alvear J Chen Y Hu ZZ Ledley RS Lewis KC Mewes HW Orcutt BC The Protein Information Resource: an integrated public resource of functional annotation of proteins Nucleic acids research 2002 30 1 35 37 10.1093/nar/30.1.35 11752247 PMC99125

[B60] Katoh K Toh H Recent developments in the MAFFT multiple sequence alignment program Brief Bioinform 2008 9 4 286 298 10.1093/bib/bbn013 18372315

[B61] Waterhouse A Procter J Martin D Clamp M Barton G Jalview Version 2 - a multiple sequence alignment editor and analysis workbench Bioinformatics 2009 25 9 1189 1191 10.1093/bioinformatics/btp033 19151095 PMC2672624

[B62] Castresana J Selection of conserved blocks from multiple alignments for their use in phylogenetic analysis Mol Biol Evol 2000 17 540 552 10.1093/oxfordjournals.molbev.a026334 10742046

[B63] Nei M Gojobori T Simple methods for estimating the numbers of synonymous and nonsynonymous nucleotide substitutions Mol Biol Evol 1986 3 5 418 426 3444411 10.1093/oxfordjournals.molbev.a040410

[B64] Ziheng Y PAML 4: Phylogenetic Analysis by Maximum Likelihood Mol Biol Evol 2007 24 8 1586 1591 10.1093/molbev/msm088 17483113

[B65] Yang Z Nielsen R Goldman N Pedersen A Codon-substitution models for heterogeneous selection pressure at amino acid sites Genetics 2000 155 1 431 449 10790415 10.1093/genetics/155.1.431 PMC1461088

[B66] Fares M Byrne K Wolfe K Rate asymmetry after genome duplication causes substantial long-branch attraction artifacts in the phylogeny of Saccharomyces species Mol Biol Evol 2006 23 2 245 253 16207937 10.1093/molbev/msj027

[B67] Yang Z Wong WNR Bayes empirical Bayes inference of amino acid sites under positive selection. Mol Biol Evol 2005 22 1107 1118 10.1093/molbev/msi097 15689528

