# Supplementary material for: In silico evolutionary analysis of Helicobacter pylori outer membrane phospholipase A (OMPLA)
Source: BMC Microbiol. 2012 Sep 13;12:206. doi: 10.1186/1471-2180-12-206 (PMC3490997; doi:10.1186/1471-2180-12-206)
Supplement: Additional file 4 — Extended majority rule consensus tree (outfiles). The outfiles that are the CONSENSE software results file from the phylogenetic trees from the phylogenetic analysis of housekeeping (Figure 1), pldA (Figure 2a and b), OMPLA (Figure 3) and AtpA (Figure 4). (RTF 405 kb) [file 1471-2180-12-206-S4.rtf]

//////////////////////////////////////////////////////////////////////////////////////////////////////////////////////////////////////////////////////////////////////////////////////////
Extended majority rule consensus tree CONSENSUS TREE:  
The numbers on the branches indicate the number of times the partition of the species into the two sets which are separated by that branch occurred among the trees, out of 1000.00 trees 
///////////////////////////////////////////////////////////////////////////////////////////////////////////////////////////////////////////////////////////////////////////////////////////

*************************************************
*Outfile pldA sequences where K80 model was used*
*************************************************

                                                                                                                          +-------afXo
          +---------------------------------------------------------------------------------------------------------341.0-|
          |                                                                                                               +-------afXn
          |
          |                                                                                                       +---------------euFY
          |                                                                                               +--25.0-|
          |                                                                                               |       |       +-------euIF
          |                                                                                               |       +-102.0-|
          |                               +----------------------------------------------------------3.00-|               +-------euHI
          |                               |                                                               |
          |                               |                                                               |               +-------euBV
          |                               |                                                               +---------333.0-|
          |                               |                                                                               +-------euAH
          |                               |
          |                               |                                                                               +-------euBD
          |                               |                                                               +---------151.0-|
          |                               |                                                               |               +-------euAF
          |                               |                                                               |
          |                               +----------------------------------------------------------1.00-|               +-------euAO
          |                               |                                                               |       +--1000-|
          |                               |                                                               |       |       +-------euAN
          |                               |                                                               +-146.0-|
          |                               |                                                                       |       +-------euAQ
          |                               |                                                                       +-999.0-|
          |                               |                                                                               +-------euAP
          |                               |
          |                               |                                                                       +---------------euXd
          |                               |                                                       +---------163.0-|
          |                               |                                                       |               |       +-------euIN
          |                               |                                                       |               +-275.0-|
          |                               |                                                       |                       +-------euDP
          |                               |                                               +--3.00-|
          |                               |                                               |       |                       +-------euDO
          |                               |                                               |       |       +---------327.0-|
          |                               |                                               |       |       |               +-------euIA
          |                               |                                               |       +--44.0-|
          |                               |                                               |               |               +-------euGT
          |                               +------------------------------------------1.00-|               |       +-437.0-|
          |                               |                                               |               +-673.0-|       +-------euHW
          |                               |                                               |                       |
          |                               |                                               |                       +---------------euIG
          |                               |                                               |
          |                               |                                               |                               +-------euBP
          |                               |                                               +-------------------------117.0-|
          |                               |                                                                               +-------euET
          |                               |
          |                               |                                                                               +-------euGM
          |                               |                                                                       +-435.0-|
          |                               |                                                               +-538.0-|       +-------euDB
          |                               |                                                               |       |
          |                               |                                               +---------386.0-|       +---------------euGK
          |                               |                                               |               |
          |                               |                                               |               |               +-------euFK
          |                               |                                               |               +---------930.0-|
          |                               |                                               |                               +-------euBL
          |                               |                                               |
          |                               +------------------------------------------12.0-|                               +-------euGE
          |                               |                                               |                       +-273.0-|
          |                               |                                               |               +-215.0-|       +-------euFO
          |                               |                                               |               |       |
          |                               |                                               |       +--18.0-|       +---------------euDC
          |                               |                                               |       |       |
          |                               |                                               |       |       |               +-------euEK
          |                               |                                               +--17.0-|       +---------140.0-|
          |                               |                                                       |                       +-------euDZ
          |                               |                                                       |
          |                               |                                                       |                       +-------euFZ
          |                               |                                                       +-----------------240.0-|
          |                               |                                                                               +-------euBA
          |                               |
          |                               |                                                                               +-------euHO
          |                               +-------------------------------------------------------------------------157.0-|
          |                               |                                                                               +-------euAB
          |                               |
          |                               |                                                                       +---------------eaXc
          |                               |                       +------------------------------------------59.0-|
          |                               |                       |                                               |       +-------eaCS
          |                               |                       |                                               +-305.0-|
          |                               |                       |                                                       +-------eaCK
          |                               |                       |
          |                               |                       |                                               +---------------eaXe
          |                               |                       |                                       +--65.0-|
          |                               |                       |                                       |       |       +-------aiXl
          |                               |                       |       +--------------------------18.0-|       +-292.0-|
          |                               |                       |       |                               |               +-------aiXb
          |                               |                       |       |                               |
          |                               |                       |       |                               +-----------------------aiXk
          |                               |                       |       |
          |                               |               +--75.0-|       |                                               +-------eaCH
          |                               |               |       |       |                                       +-628.0-|
          |                               |               |       |       |                               +-505.0-|       +-------eaCF
          |                               |               |       |       |                               |       |
          |                               |               |       |       |                       +-228.0-|       +---------------eaCG
          |                               |               |       |       |                       |       |
          |                               |               |       |       |               +--5.00-|       +-----------------------euXf
          |                               |               |       |       |               |       |
          |                               |               |       |       |               |       |                       +-------eaCR
          |                               |               |       |       |               |       +-----------------307.0-|
          |                               |               |       |       |       +--3.00-|                               +-------eaCI
          |                               |               |       |       |       |       |
          |                               |               |       |       |       |       |                               +-------Xs
          |                               |               |       +--3.00-|       |       |                       +-993.0-|
          |                               |               |               |       |       +------------------77.0-|       +-------eaCJ
          |                               |               |               |       |                               |
          |                               |               |               +--1.00-|                               +---------------eaCN
          |                               |               |               |       |
          |                               |               |               |       |                                       +-------eaCQ
          |                               |               |               |       |                               +-398.0-|
          |                               |       +-130.0-|               |       |                               |       +-------eaCL
          |                               |       |       |               |       |                       +-155.0-|
          |                               |       |       |               |       |                       |       |       +-------eaXh
          |                               |       |       |               |       +-----------------119.0-|       +-387.0-|
          |                               |       |       |               |                               |               +-------eaCT
          |                               |       |       |               |                               |
          |                               |       |       |               |                               +-----------------------euBQ
          |                               |       |       |               |
          |                               |       |       |               |                                       +---------------eaCE
          |                               |       |       |               |                               +--50.0-|
          |                               |       |       |               |                               |       |       +-------eaCC
          |                               |       |       |               |                               |       +-302.0-|
          |                               +--3.00-|       |               +--------------------------40.0-|               +-------eaCB
          |                               |       |       |                                               |
          |                               |       |       |                                               |               +-------eaCM
          |                               |       |       |                                               +---------997.0-|
          |                               |       |       |                                                               +-------eaCD
          |                               |       |       |
          |                               |       |       |                                                               +-------eaCO
          |                               |       |       |                                                       +-351.0-|
          |                               |       |       +-------------------------------------------------150.0-|       +-------eaCP
          |                               |       |                                                               |
          |                               |       |                                                               +---------------euAL
          |                               |       |
          |                               |       |                                                                       +-------euFL
          |                               |       +------------------------------------------------------------------66.0-|
          |                               |                                                                               +-------euXg
          |                               |
          |                               |                                                                               +-------euIH
          |                               |                                                                       +-340.0-|
          |                               |                                                                       |       +-------euHA
          |                               |                                       +--------------------------65.0-|
          |                               |                                       |                               |       +-------euBC
          |                               |                                       |                               +-219.0-|
          |                               |                                       |                                       +-------euFH
          |                               |                                       |
          |                               |                                       |                       +-----------------------euHV
          |                               |                               +--6.00-|               +-420.0-|
          |                               |                               |       |               |       |       +---------------euHK
          |                               |                               |       |               |       +-194.0-|
          |                               |                               |       |       +-973.0-|               |       +-------euHC
          |                               |                               |       |       |       |               +-173.0-|
          |                               |                               |       |       |       |                       +-------euEP
          |                               +--------------------------1.00-|       +--42.0-|       |
          |                               |                               |               |       +-------------------------------euIC
          |                               |                               |               |
          |                               |                               |               +---------------------------------------euHJ
          |                               |                               |
          |                               |                               |                                       +---------------euHR
          |                               |                               +----------------------------------77.0-|
          |                               |                                                                       |       +-------euED
          |                               |                                                                       +-834.0-|
          |                               |                                                                               +-------euHH
          |                               |
          |                               |                                                                       +---------------euEY
          |                               +-----------------------------------------------------------------116.0-|
          |                               |                                                                       |       +-------euBO
          |                               |                                                                       +-192.0-|
          |                               |                                                                               +-------euCW
          |                               |
          |                               |                                                                               +-------euII
          |                               |                                                       +-----------------195.0-|
          |                               |                                                       |                       +-------euHD
          |                               |                                                       |
          |                               +--------------------------------------------------1.00-|                       +-------euDM
          |                               |                                                       |               +-460.0-|
          |                               |                                                       |       +-108.0-|       +-------euHZ
          |                               |                                                       |       |       |
          |                               |                                                       +--20.0-|       +---------------euAV
          |                               |                                                               |
          |                               |                                                               +-----------------------euDS
          |                               |
          |                               |                                                                       +---------------euHX
          |                               |                                                               +-683.0-|
          |                               |                                                               |       |       +-------euDX
          |                               |                                                               |       +-296.0-|
          |                               |                                                       +-865.0-|               +-------euDR
          |                               |                                                       |       |
          |                               |                                                       |       |               +-------euFR
          |                               |                                               +-103.0-|       +---------263.0-|
          |                               |                                               |       |                       +-------euDN
          |                               |                                       +--75.0-|       |
          |                               |                                       |       |       +-------------------------------euFE
          |                               |                                       |       |
          |                               +----------------------------------28.0-|       +---------------------------------------afXa
          |                               |                                       |
          |                               |                                       |                                       +-------euAS
          |                               |                                       +----------------------------------1000-|
          |                               |                                                                               +-------euBF
          |                               |
          |                               |                                                                       +---------------euFM
          |                               |                                                               +-189.0-|
          |                               |                                                               |       |       +-------euHB
          |                               |                                                               |       +-900.0-|
          |                               |                                                       +--57.0-|               +-------euDU
          |                               |                                                       |       |
          |                               |                                                       |       |               +-------euGZ
          |                               |                                                       |       |       +-895.0-|
          |                               |                                                       |       +-906.0-|       +-------euFG
          |                               +--------------------------------------------------2.00-|               |
          |                               |                                                       |               +---------------euHM
          |                               |                                                       |
          |                               |                                                       |                       +-------euDQ
          |                               |                                                       |               +-992.0-|
          |                               |                                                       +---------241.0-|       +-------euGV
          |                               |                                                                       |
          |                               |                                                                       +---------------euDA
          |                               |
          |                               |                                                                               +-------euGY
          |                               |                                                                       +-657.0-|
          |                               |                                                                       |       +-------euFS
          |                               |                                                               +-223.0-|
          |                               |                                                               |       |       +-------euHN
          |                               |                                                       +-852.0-|       +-244.0-|
          |                               |                                                       |       |               +-------euFQ
          |                               |                                                       |       |
          |                               |                                               +-187.0-|       +-----------------------euIQ
          |                               |                                               |       |
  +-------|                               |                                               |       |                       +-------euDW
  |       |                               +------------------------------------------11.0-|       +-----------------997.0-|
  |       |                               |                                               |                               +-------euIE
  |       |                               |                                               |
  |       |                               |                                               |                               +-------euIL
  |       |                               |                                               +-------------------------988.0-|
  |       |                               |                                                                               +-------euIO
  |       |                               |
  |       |                       +--25.0-|                                                                               +-------euHS
  |       |                       |       |                                                       +-----------------152.0-|
  |       |                       |       |                                                       |                       +-------euAD
  |       |                       |       |                                                       |
  |       |                       |       +--------------------------------------------------2.00-|                       +-------euGR
  |       |                       |       |                                                       |               +-160.0-|
  |       |                       |       |                                                       |       +-107.0-|       +-------euDL
  |       |                       |       |                                                       |       |       |
  |       |                       |       |                                                       +--26.0-|       +---------------euEW
  |       |                       |       |                                                               |
  |       |                       |       |                                                               +-----------------------euAE
  |       |                       |       |
  |       |                       |       |                                                       +-------------------------------euBX
  |       |                       |       |                                                       |
  |       |                       |       |                                                       |                       +-------euGD
  |       |                       |       +--------------------------------------------------8.00-|               +-675.0-|
  |       |                       |       |                                                       |               |       +-------euGQ
  |       |                       |       |                                                       |       +-863.0-|
  |       |                       |       |                                                       |       |       |       +-------euDY
  |       |                       |       |                                                       +-125.0-|       +-282.0-|
  |       |                       |       |                                                               |               +-------euHT
  |       |                       |       |                                                               |
  |       |                       |       |                                                               +-----------------------euIP
  |       |                       |       |
  |       |                       |       |                                                                               +-------euXi
  |       |                       |       |                                                               +---------115.0-|
  |       |                       |       |                                                               |               +-------afXt
  |       |                       |       +----------------------------------------------------------1.00-|
  |       |                       |       |                                                               |       +---------------euAA
  |       |                       |       |                                                               +--77.0-|
  |       |                       |       |                                                                       |       +-------euCY
  |       |                       |       |                                                                       +-243.0-|
  |       |                       |       |                                                                               +-------euBM
  |       |                       |       |
  |       |                       |       |                               +-------------------------------------------------------euBZ
  |       |                       |       |                               |
  |       |                       |       |                               |                                               +-------euBN
  |       |                       |       |                               |                               +---------107.0-|
  |       |                       |       |                               |                               |               +-------euGB
  |       |                       |       |                               |                       +--28.0-|
  |       |                       |       |                               |                       |       |               +-------euAX
  |       |                       |       |                               |                       |       |       +-740.0-|
  |       |                       |       |                               |                       |       +-305.0-|       +-------euIS
  |       |                       |       |                               |                       |               |
  |       |                       |       |                               |               +--94.0-|               +---------------euFI
  |       |                       |       |                       +--57.0-|               |       |
  |       |                       |       |                       |       |               |       |               +---------------euDD
  |       |                       |       |                       |       |               |       |       +--96.0-|
  |       |                       |       |                       |       |               |       |       |       |       +-------euEG
  |       |                       |       |                       |       |               |       +--20.0-|       +-385.0-|
  |       |                       |       |                       |       |               |               |               +-------euBR
  |       |                       |       |                       |       |       +-219.0-|               |
  |       |                       |       |                       |       |       |       |               +-----------------------euBU
  |       |                       |       |                       |       |       |       |
  |       |                       |       |                       |       |       |       |                               +-------euGI
  |       |                       |       |                       |       |       |       |                       +-973.0-|
  |       |                       |       |                       |       |       |       |               +-419.0-|       +-------euDG
  |       |                       |       +------------------3.00-|       +--83.0-|       |               |       |
  |       |                       |       |                       |               |       +----------67.0-|       +---------------euBH
  |       |                       |       |                       |               |                       |
  |       |                       |       |                       |               |                       |               +-------euFT
  |       |                       |       |                       |               |                       +---------408.0-|
  |       |                       |       |                       |               |                                       +-------euIB
  |       |                       |       |                       |               |
  |       |                       |       |                       |               +-----------------------------------------------euES
  |       |                       |       |                       |
  |       |                       |       |                       |                                                       +-------euEU
  |       |                       |       |                       |                                               +-219.0-|
  |       |                       |       |                       |                                               |       +-------euCU
  |       |                       |       |                       +------------------------------------------49.0-|
  |       |                       |       |                                                                       |       +-------euDE
  |       |                       |       |                                                                       +-322.0-|
  |       |                       |       |                                                                               +-------euHU
  |       |                       |       |
  |       |                       |       |                                                                       +---------------euFW
  |       |                       |       |                                                               +--1000-|
  |       |                       |       |                                                               |       |       +-------euFC
  |       |                       |       |                                                       +-126.0-|       +-887.0-|
  |       |                       |       |                                                       |       |               +-------euFP
  |       |                       |       |                                                       |       |
  |       |                       |       +--------------------------------------------------3.00-|       +-----------------------Xm
  |       |                       |       |                                                       |
  |       |                       |       |                                                       |                       +-------euGP
  |       |                       |       |                                                       +-----------------194.0-|
  |       |                       |       |                                                                               +-------euDV
  |       |                       |       |
  |       |                       |       |                                                                               +-------euFD
  |       |                       |       |                                                                       +-991.0-|
  |       |                       |       |                                                                       |       +-------euIM
  |       |                       |       |                                                               +-210.0-|
  |       |                       |       |                                                               |       |       +-------euGS
  |       |                       |       +----------------------------------------------------------9.00-|       +-367.0-|
  |       |                       |       |                                                               |               +-------euHF
  |       |                       |       |                                                               |
  |       |                       |       |                                                               +-----------------------euAZ
  |       |                       |       |
  |       |                       |       |                                                                               +-------euEM
  |       |                       |       |                                                                       +-360.0-|
  |       |                       |       |                                                               +-494.0-|       +-------euIR
  |       |                       |       |                                                               |       |
  |       |                       |       |                                                       +-159.0-|       +---------------euEF
  |       |                       |       |                                                       |       |
  |       |                       |       |                                                       |       |               +-------euDK
  |       |                       |       |                                       +----------72.0-|       +---------504.0-|
  |       |                       |       |                                       |               |                       +-------euDH
  |       |                       |       |                                       |               |
  |       |                       |       |                                       |               +-------------------------------euBE
  |       |                       |       |                                       |
  |       |                       |       |                                       |                                       +-------euEN
  |       |                       |       +----------------------------------2.00-|                               +-246.0-|
  |       |                       |       |                                       |                       +-246.0-|       +-------euHL
  |       |                       |       |                                       |                       |       |
  |       |                       |       |                                       |               +-928.0-|       +---------------euEA
  |       |                       |       |                                       |               |       |
  |       |                       |       |                                       |       +-444.0-|       +-----------------------euGW
  |       |               +-111.0-|       |                                       |       |       |
  |       |               |       |       |                                       +--30.0-|       +-------------------------------euDF
  |       |               |       |       |                                               |
  |       |               |       |       |                                               |                               +-------euGH
  |       |               |       |       |                                               +-------------------------395.0-|
  |       |               |       |       |                                                                               +-------euAY
  |       |               |       |       |
  |       |               |       |       |                                                                               +-------euEV
  |       |               |       |       |                                                                       +-771.0-|
  |       |               |       |       |                                                                       |       +-------euEZ
  |       |               |       |       |                                               +------------------59.0-|
  |       |               |       |       |                                               |                       |       +-------euEJ
  |       |               |       |       |                                               |                       +-343.0-|
  |       |               |       |       |                                               |                               +-------euAT
  |       |               |       |       |                                               |
  |       |               |       |       |                                               |                               +-------euCZ
  |       |               |       |       |                                       +--7.00-|                       +-720.0-|
  |       |               |       |       |                                       |       |               +-822.0-|       +-------euFU
  |       |               |       |       |                                       |       |               |       |
  |       |               |       |       |                                       |       |       +-328.0-|       +---------------euCV
  |       |               |       |       |                                       |       |       |       |
  |       |               |       |       |                                       |       |       |       |               +-------euEL
  |       |               |       |       |                                       |       +--27.0-|       +---------470.0-|
  |       |               |       |       +----------------------------------1.00-|               |                       +-------euAC
  |       |               |       |       |                                       |               |
  |       |               |       |       |                                       |               |                       +-------euDT
  |       |               |       |       |                                       |               +-----------------153.0-|
  |       |               |       |       |                                       |                                       +-------euAR
  |       |               |       |       |                                       |
  |       |               |       |       |                                       |                                       +-------euEO
  |       |               |       |       |                                       +----------------------------------40.0-|
  |       |               |       |       |                                                                               +-------euAG
  |       |               |       |       |
  |       |               |       |       |                                                                               +-------euAM
  |       |               |       |       |                                                                       +-132.0-|
  |       |               |       |       +------------------------------------------------------------------6.00-|       +-------euFX
  |       |               |       |       |                                                                       |
  |       |               |       |       |                                                                       +---------------euBI
  |       |               |       |       |
  |       |               |       |       |                                                                               +-------euBS
  |       |               |       |       |                                                                       +-164.0-|
  |       |               |       |       |                                                                       |       +-------euBK
  |       |               |       |       |                                                               +--20.0-|
  |       |               |       |       |                                                               |       |       +-------euFV
  |       |               |       |       |                                                               |       +-417.0-|
  |       |               |       |       +----------------------------------------------------------1.00-|               +-------euBJ
  |       |               |       |       |                                                               |
  |       |               |       |       |                                                               |               +-------aiXj
  |       |               |       |       |                                                               |       +-219.0-|
  |       |               |       |       |                                                               +--31.0-|       +-------euGX
  |       |               |       |       |                                                                       |
  |       |               |       |       |                                                                       +---------------euEC
  |       |               |       |       |
  |       |               |       |       |                                                                               +-------euXq
  |       |               |       |       |                                                                       +--97.0-|
  |       |               |       |       |                                                                       |       +-------euEI
  |       |               |       |       |                                                               +--11.0-|
  |       |               |       |       |                                                               |       |       +-------euHP
  |       |               |       |       +----------------------------------------------------------2.00-|       +-179.0-|
  |       |               |       |       |                                                               |               +-------euFF
  |       |               |       |       |                                                               |
  |       |               |       |       |                                                               +-----------------------euAK
  |       |               |       |       |
  |       |               |       |       |                                                                       +---------------euBG
  |       |               |       |       |                                                       +---------198.0-|
  |       |               |       |       |                                                       |               |       +-------euGL
  |       |               |       |       |                                                       |               +-913.0-|
  |       |       +-407.0-|       |       |                                                       |                       +-------euIK
  |       |       |       |       |       +--------------------------------------------------20.0-|
  |       |       |       |       |       |                                                       |               +---------------euID
  |       |       |       |       |       |                                                       |       +-958.0-|
  |       |       |       |       |       |                                                       |       |       |       +-------euGN
  |       |       |       |       |       |                                                       +-144.0-|       +-555.0-|
  |       |       |       |       |       |                                                               |               +-------euFA
  |       |       |       |       |       |                                                               |
  |       |       |       |       |       |                                                               +-----------------------euBW
  |       |       |       |       |       |
  |       |       |       |       |       |                                                                               +-------euHE
  |       |       |       |       |       |                                                                       +-122.0-|
  |       |       |       |       |       |                                                                       |       +-------euEB
  |       |       |       |       |       +------------------------------------------------------------------3.00-|
  |       |       |       |       |       |                                                                       |       +-------euBB
  |       |       |       |       |       |                                                                       +-224.0-|
  |       |       |       |       |       |                                                                               +-------euFB
  |       |       |       |       |       |
  |       |       |       |       |       |                                                                       +---------------euDI
  |       |       |       |       |       +-----------------------------------------------------------------164.0-|
  |       |       |       |       |       |                                                                       |       +-------euEE
  |       |       |       |       |       |                                                                       +-977.0-|
  |       |       |       |       |       |                                                                               +-------euEH
  |       |       |       |       |       |
  |       |       |       |       |       |                                                                               +-------euGJ
  |       |       |       |       |       |                                                                       +-580.0-|
  |       |       |       |       |       |                                                               +-909.0-|       +-------euAJ
  |       |       |       |       |       |                                                               |       |
  |       |       |       |       |       |                                                       +--36.0-|       +---------------euAI
  |       |       |       |       |       |                                                       |       |
  |       |       |       |       |       +--------------------------------------------------3.00-|       +-----------------------euGO
  |       |       |       |       |       |                                                       |
  |       |       |       |       |       |                                                       |                       +-------euFJ
  |       |       |       |       |       |                                                       +------------------94.0-|
  |       |       |       |       |       |                                                                               +-------euCX
  |       |       |       |       |       |
  |       +--1000-|       |       |       |                                                                               +-------euAU
  |               |       |       |       |                                                                       +-148.0-|
  |               |       |       |       |                                                                       |       +-------euGG
  |               |       |       |       +------------------------------------------------------------------3.00-|
  |               |       |       |       |                                                                       |       +-------euHQ
  |               |       |       |       |                                                                       +-108.0-|
  |               |       |       |       |                                                                               +-------euGU
  |               |       |       |       |
  |               |       |       |       |                                                                               +-------euBY
  |               |       |       |       +--------------------------------------------------------------------------12.0-|
  |               |       |       |                                                                                       +-------eaCA
  |               |       |       |
  |               |       |       |                                                                       +-----------------------euAW
  |               |       |       +------------------------------------------------------------------93.0-|
  |               |       |                                                                               |       +---------------euIJ
  |               |       |                                                                               +-943.0-|
  |               |       |                                                                                       |       +-------euGC
  |               |       |                                                                                       +-555.0-|
  |               |       |                                                                                               +-------euDJ
  |               |       |
  |               |       |                                                                                               +-------euGA
  |               |       |                                                                                       +-139.0-|
  |               |       |                                                                                       |       +-------euER
  |               |       |                                                                               +-223.0-|
  |               |       |                                                                               |       |       +-------euHG
  |               |       |                                                                       +-942.0-|       +-151.0-|
  |               |       |                                                                       |       |               +-------euEQ
  |               |       |                                                               +-871.0-|       |
  |               |       |                                                               |       |       +-----------------------euEX
  |               |       |                                                       +-551.0-|       |
  |               |       |                                                       |       |       +-------------------------------euGF
  |               |       +-------------------------------------------------420.0-|       |
  |               |                                                               |       +---------------------------------------euFN
  |               |                                                               |
  |               |                                                               +-----------------------------------------------euHY
  |               |
  |               +---------------------------------------------------------------------------------------------------------------euBT
  |
  +-------------------------------------------------------------------------------------------------------------------------------afXp

*************************************************
*Outfile pldA sequences where GTR model was used*
*************************************************

                                                                                                                          +-------------------------------euDF
                                                                                                                          |
                                                                                                                  +-473.0-|       +-----------------------euEN
                                                                                                                  |       |       |
                                                                                                                  |       +--1000-|               +-------euGW
                                                                                                                  |               |       +-701.0-|
                                                                                                                  |               +-843.0-|       +-------euEA
                                                                                                          +--13.0-|                       |
                                                                                                          |       |                       +---------------euHL
                                                                                                          |       |
                                                                                                          |       |                               +-------euEH
                                                                                                          |       |                       +--1000-|
                                                                                                          |       +-----------------252.0-|       +-------euEE
                                                                                                          |                               |
                                  +------------------------------------------------------------------1.00-|                               +---------------euDI
                                  |                                                                       |
                                  |                                                                       |                                       +-------euDL
                                  |                                                                       |                               +-136.0-|
                                  |                                                                       |                               |       +-------euAE
                                  |                                                                       |                       +--11.0-|
                                  |                                                                       |                       |       |       +-------euEW
                                  |                                                                       +------------------9.00-|       +-117.0-|
                                  |                                                                                               |               +-------euGR
                                  |                                                                                               |
                                  |                                                                                               +-----------------------euBY
                                  |
                                  |                                                                                                               +-------euGZ
                                  |                                                                                                       +-988.0-|
                                  |                                                                                               +-962.0-|       +-------euFG
                                  |                                                                                               |       |
                                  +------------------------------------------------------------------------------------------80.0-|       +---------------euHM
                                  |                                                                                               |
                                  |                                                                                               |       +---------------euFM
                                  |                                                                                               +-349.0-|
                                  |                                                                                                       |       +-------euDU
                                  |                                                                                                       +-996.0-|
                                  |                                                                                                               +-------euHB
                                  |
                                  |                                               +-----------------------------------------------------------------------eaCA
                                  |                                               |
                                  |                                               |                                       +-------------------------------euEX
                                  |                                               |                                       |
                                  |                                               |                                       |                       +-------euER
                                  |                                               |                               +-982.0-|               +-159.0-|
                                  |                                               |                               |       |       +-277.0-|       +-------euHG
                                  +------------------------------------------51.0-|                               |       |       |       |
                                  |                                               |                       +-926.0-|       +-964.0-|       +---------------euEQ
                                  |                                               |                       |       |               |
                                  |                                               |                       |       |               +-----------------------euGA
                                  |                                               |               +-689.0-|       |
                                  |                                               |               |       |       +---------------------------------------euGF
                                  |                                               |       +-564.0-|       |
                                  |                                               |       |       |       +-----------------------------------------------euFN
                                  |                                               +-111.0-|       |
                                  |                                                       |       +-------------------------------------------------------euHY
                                  |                                                       |
                                  |                                                       +---------------------------------------------------------------euXg
                                  |
                                  |                                                                                                       +---------------euFY
                                  |                                                                                               +--61.0-|
                                  |                                                                                               |       |       +-------euHH
                                  |                                                                                               |       +-953.0-|
                                  +------------------------------------------------------------------------------------------4.00-|               +-------euED
                                  |                                                                                               |
                                  |                                                                                               |               +-------euAB
                                  |                                                                                               |       +-201.0-|
                                  |                                                                                               +--63.0-|       +-------euHO
                                  |                                                                                                       |
                                  |                                                                                                       +---------------euEO
                                  |
                                  |                                                                                                               +-------euDP
                                  |                                                                                                       +-370.0-|
                                  |                                                                                               +-245.0-|       +-------euIN
                                  |                                                                                               |       |
                                  |                                                                                       +--11.0-|       +---------------euXd
                                  |                                                                                       |       |
                                  |                                                                                       |       |               +-------euBP
                                  |                                                                                       |       +---------193.0-|
                                  |               +------------------------------------------------------------------4.00-|                       +-------euET
                                  |               |                                                                       |
                                  |               |                                                                       |                       +-------euDO
                                  |               |                                                                       |       +---------232.0-|
                                  |               |                                                                       |       |               +-------euIA
                                  |               |                                                                       +--26.0-|
                                  |               |                                                                               |               +-------euGT
                                  |               |                                                                               |       +-533.0-|
                                  |               |                                                                               +-646.0-|       +-------euHW
                                  |               |                                                                                       |
                                  |               |                                                                                       +---------------euIG
                                  |               |
                                  |               |                                                                                               +-------aiXj
                                  |               |                                                                                       +-215.0-|
                                  |               |               +------------------------------------------------------------------47.0-|       +-------euGX
                                  |               |               |                                                                       |
                                  |               |               |                                                                       +---------------euFL
                                  |               |               |
                                  |               |               |                                                                               +-------eaCD
                                  |               |               |                                                               +---------997.0-|
                                  |               |               |                                                               |               +-------eaCM
                                  |               |               |                                                               |
                                  |               |               |                                                       +--40.0-|               +-------eaCT
                                  |               |               |                                                       |       |       +-419.0-|
                                  |               |               |                                                       |       |       |       +-------eaXh
                                  |               |               |                                                       |       +-251.0-|
                                  |               |               |                                                       |               |       +-------Xs
                                  |               |               |                                                       |               +-997.0-|
                                  |               |               |                                               +--32.0-|                       +-------eaCJ
                                  |       +--2.00-|               |                                               |       |
                                  |       |       |               |                                               |       |                       +-------eaCF
                                  |       |       |               |                                               |       |               +-719.0-|
                                  |       |       |               |                                               |       |       +-536.0-|       +-------eaCH
                                  |       |       |               |                                               |       |       |       |
                                  |       |       |               |                                       +--4.00-|       +-204.0-|       +---------------eaCG
                                  |       |       |               |                                       |       |               |
                                  |       |       |               |                                       |       |               +-----------------------euXf
                                  |       |       |               |                                       |       |
                                  |       |       |               |                                       |       |                               +-------eaCI
                                  |       |       |               |                                       |       |                       +-359.0-|
                                  |       |       |               |                                       |       +------------------84.0-|       +-------eaCR
                                  |       |       |       +--45.0-|                                       |                               |
                                  |       |       |       |       |                               +--3.00-|                               +---------------euBQ
                                  |       |       |       |       |                               |       |
                                  |       |       |       |       |                               |       |                                       +-------eaCB
                                  |       |       |       |       |                               |       |                       +---------424.0-|
                                  |       |       |       |       |                               |       |                       |               +-------eaCC
                                  |       |       |       |       |                               |       |               +--73.0-|
                                  |       |       |       |       |                               |       |               |       |               +-------eaCQ
                                  |       |       |       |       |                               |       |               |       |       +-418.0-|
                                  |       |       |       |       |                       +--4.00-|       +----------11.0-|       +-120.0-|       +-------eaCL
                                  |       |       |       |       |                       |       |                       |               |
                                  |       |       |       |       |                       |       |                       |               +---------------eaXe
                                  |       |       |       |       |                       |       |                       |
                                  |       |       |       |       |                       |       |                       +-------------------------------aiXk
                                  |       |       |       |       |                       |       |
                                  |       |       |       |       |               +--48.0-|       |                                               +-------aiXb
                                  |       |       |       |       |               |       |       |                                       +-294.0-|
                                  |       |       |       |       |               |       |       +----------------------------------78.0-|       +-------aiXl
                                  |       |       +--13.0-|       |               |       |                                               |
                                  |       |               |       |               |       |                                               +---------------eaCE
                                  |       |               |       |               |       |
                                  |       |               |       |       +--92.0-|       |                                               +---------------eaXc
                                  |       |               |       |       |       |       +------------------------------------------62.0-|
                                  |       |               |       |       |       |                                                       |       +-------eaCK
                                  |       |               |       |       |       |                                                       +-347.0-|
                                  |       |               |       |       |       |                                                               +-------eaCS
                                  |       |               |       |       |       |
                                  +--1.00-|               |       +-161.0-|       |                                                       +---------------euAL
                                  |       |               |               |       +-------------------------------------------------195.0-|
                                  |       |               |               |                                                               |       +-------eaCP
                                  |       |               |               |                                                               +-340.0-|
                                  |       |               |               |                                                                       +-------eaCO
                                  |       |               |               |
                                  |       |               |               +-------------------------------------------------------------------------------eaCN
                                  |       |               |
                                  |       |               |                                                                                       +-------euIL
                                  |       |               +----------------------------------------------------------------------------------1000-|
                                  |       |                                                                                                       +-------euIO
                                  |       |
                                  |       |                                                                                                       +-------euEK
                                  |       |                                                                                       +---------174.0-|
                                  |       |                                                                                       |               +-------euDZ
                                  |       |                                                                               +--30.0-|
                                  |       |                                                                               |       |               +-------euGE
                                  |       |                                                                               |       |       +-270.0-|
                                  |       |                                                                               |       +-222.0-|       +-------euFO
                                  |       |                                                                       +--31.0-|               |
                                  |       |                                                                       |       |               +---------------euDC
                                  |       |                                                                       |       |
                                  |       |                                                                       |       |                       +-------euBA
                                  |       |                                                                       |       +-----------------297.0-|
                                  |       +------------------------------------------------------------------16.0-|                               +-------euFZ
                                  |       |                                                                       |
                                  |       |                                                                       |                               +-------euGM
                                  |       |                                                                       |                       +-419.0-|
                                  |       |                                                                       |               +-693.0-|       +-------euDB
                                  |       |                                                                       |               |       |
                                  |       |                                                                       +---------428.0-|       +---------------euGK
                                  |       |                                                                                       |
                                  |       |                                                                                       |               +-------euBL
                                  |       |                                                                                       +---------972.0-|
                                  |       |                                                                                                       +-------euFK
                                  |       |
                                  |       |                                                                                                       +-------euAY
                                  |       |                                                                               +-----------------497.0-|
                                  |       |                                                                               |                       +-------euGH
                                  |       |                                                                               |
                                  |       +--------------------------------------------------------------------------50.0-|                       +-------euEM
                                  |                                                                                       |               +-366.0-|
                                  |                                                                                       |       +-540.0-|       +-------euIR
                                  |                                                                                       |       |       |
                                  |                                                                                       +-195.0-|       +---------------euEF
                                  |                                                                                               |
                                  |                                                                                               |               +-------euDK
                                  |                                                                                               +---------631.0-|
                                  |                                                                                                               +-------euDH
                                  |
                                  |                                                                                                               +-------euHE
                                  |                                                                                                       +-180.0-|
                                  |                                                                                                       |       +-------euEB
                                  |                                                                       +--------------------------8.00-|
                                  |                                                                       |                               |       +-------euFB
                                  |                                                                       |                               +-325.0-|
                                  |                                                                       |                                       +-------euBB
                                  |                                                                       |
                                  |                                                               +--10.0-|                                       +-------euAF
                                  |                                                               |       |       +-------------------------243.0-|
                                  |                                                               |       |       |                               +-------euBD
                                  |                                                               |       |       |
                                  |                                                               |       |       |               +-----------------------euHV
                                  |                                                               |       +--30.0-|               |
                                  |                                                               |               |       +-849.0-|               +-------euEP
                                  |                                                               |               |       |       |       +-273.0-|
                                  |                                                               |               |       |       +-646.0-|       +-------euHK
                                  +----------------------------------------------------------5.00-|               +-989.0-|               |
                                  |                                                               |                       |               +---------------euHC
                                  |                                                               |                       |
                                  |                                                               |                       +-------------------------------euIC
                                  |                                                               |
                                  |                                                               |                                               +-------euFC
                                  |                                                               |                                       +-927.0-|
                                  |                                                               |                               +--1000-|       +-------euFP
                                  |                                                               |                               |       |
                                  |                                                               +-------------------------270.0-|       +---------------euFW
                                  |                                                                                               |
                                  |                                                                                               +-----------------------Xm
                                  |
                                  |                                                                                               +-----------------------euAW
                                  |                                                                                               |
                                  |                                                                                       +-115.0-|               +-------euDJ
                                  |                                                                                       |       |       +-674.0-|
                                  |                                                                                       |       +-988.0-|       +-------euGC
                                  |                                                                                       |               |
                                  +----------------------------------------------------------------------------------3.00-|               +---------------euIJ
                                  |                                                                                       |
                                  |                                                                                       |                       +-------euDE
                                  |                                                                                       |               +-355.0-|
                                  |                                                                                       |               |       +-------euAD
                                  |                                                                                       +----------35.0-|
                                  |                                                                                                       |       +-------euAH
                                  |                                                                                                       +-344.0-|
                                  |                                                                                                               +-------euBV
                                  |
                                  |                                                                                               +-----------------------euDY
                          +--7.00-|                                                                                               |
                          |       |                                                                                       +-970.0-|               +-------euGD
                          |       |                                                                                       |       |       +-726.0-|
                          |       |                                                                                       |       +-785.0-|       +-------euGQ
                          |       |                                                                               +-150.0-|               |
                          |       |                                                                               |       |               +---------------euHT
                          |       +--------------------------------------------------------------------------4.00-|       |
                          |       |                                                                               |       +-------------------------------euIP
                          |       |                                                                               |
                          |       |                                                                               +---------------------------------------euBX
                          |       |
                          |       |                                                                                                               +-------euAM
                          |       |                                                                                                       +-144.0-|
                          |       |                                                                                                       |       +-------euFX
                          |       +--------------------------------------------------------------------------------------------------90.0-|
                          |       |                                                                                                       |       +-------euEJ
                          |       |                                                                                                       +-479.0-|
                          |       |                                                                                                               +-------euAT
                          |       |
                          |       |                                                                                       +-------------------------------euDN
                          |       |                                                                                       |
                          |       |                                                                               +-972.0-|       +-----------------------euFR
                          |       |                                                                               |       |       |
                          |       |                                                                               |       +-652.0-|               +-------euDX
                          |       |                                                                               |               |       +-308.0-|
                          |       |                                                                       +-101.0-|               +-699.0-|       +-------euDR
                          |       |                                                                       |       |                       |
                          |       |                                                                       |       |                       +---------------euHX
                          |       |                                                                       |       |
                          |       +------------------------------------------------------------------6.00-|       +---------------------------------------euFE
                          |       |                                                                       |
                          |       |                                                                       |       +---------------------------------------afXa
                          |       |                                                                       |       |
                          |       |                                                                       +-100.0-|       +-------------------------------afXt
                          |       |                                                                               |       |
                          |       |                                                                               +-230.0-|       +-----------------------euBT
                          |       |                                                                                       |       |
                          |       |                                                                                       +-464.0-|               +-------afXp
                          |       |                                                                                               |       +-365.0-|
                          |       |                                                                                               +--1000-|       +-------afXo
                          |       |                                                                                                       |
                          |       |                                                                                                       +---------------afXn
                          |       |
                          |       |                                                                                                               +-------euFS
                          |       |                                                                                               +---------652.0-|
                          |       |                                                                                               |               +-------euGY
                          |       |                                                                                       +-985.0-|
                          |       |                                                                                       |       |               +-------euFQ
                          |       |                                                                                       |       |       +-572.0-|
                          |       |                                                                                       |       +-502.0-|       +-------euHN
                          |       |                                                                               +-237.0-|               |
                          |       |                                                                               |       |               +---------------euIQ
                          |       |                                                                               |       |
                          |       +--------------------------------------------------------------------------51.0-|       |                       +-------euIE
                          |       |                                                                               |       +------------------1000-|
                          |       |                                                                               |                               +-------euDW
                          |       |                                                                               |
                          |       |                                                                               +---------------------------------------euHS
                          |       |
                          |       |                                                                                                               +-------euFD
                          |       |                                                                                                       +--1000-|
                          |       |                                                                                                       |       +-------euIM
                          |       |                                                                                               +-332.0-|
                          |       |                                                                                               |       |       +-------euHF
                          |       |                                                                                               |       +-424.0-|
                          |       +------------------------------------------------------------------------------------------11.0-|               +-------euGS
                          |       |                                                                                               |
                          |       |                                                                                               |               +-------euGV
                          |       |                                                                                               |       +--1000-|
                          |       |                                                                                               +-289.0-|       +-------euDQ
                          |       |                                                                                                       |
                          |       |                                                                                                       +---------------euDA
                          |       |
                          |       |                                                                                                               +-------euHZ
                          |       |                                                                                                       +-480.0-|
                          |       |                                                                                               +-143.0-|       +-------euDM
                          |       |                                                                                               |       |
                          |       |                                                                                       +--14.0-|       +---------------euAV
                          |       |                                                                                       |       |
                          |       |                                                                                       |       |               +-------euDS
                          |       |                                                                                       |       +---------191.0-|
                          |       +----------------------------------------------------------------------------------2.00-|                       +-------euDT
                          |       |                                                                                       |
                          |       |                                                                                       |                       +-------euAQ
                          |       |                                                                                       |               +--1000-|
                          |       |                                                                                       |               |       +-------euAP
                          |       |                                                                                       +---------208.0-|
                          |       |                                                                                                       |       +-------euAO
                          |       |                                                                                                       +--1000-|
                          |       |                                                                                                               +-------euAN
                          |       |
                          |       |                                                                                                               +-------euHR
                          |       |                                                                                                       +-145.0-|
                          |       |                                                                                               +--81.0-|       +-------euGU
                          |       |                                                                                               |       |
                          |       +------------------------------------------------------------------------------------------6.00-|       +---------------euHQ
                          |       |                                                                                               |
                          |       |                                                                                               |               +-------euHI
                          |       |                                                                                               +---------131.0-|
                          |       |                                                                                                               +-------euIF
                          |       |
                          |       |                                                                                                               +-------euGB
                          |       |                                                                                                       +-258.0-|
                          |       |                                                               +---------------------------------186.0-|       +-------euBR
                          |       |                                                               |                                       |
                          |       |                                                               |                                       +---------------euFI
                          |       |                                                               |
                          |       |                                                               |                                               +-------euDG
                          |       |                                                               |                                       +--1000-|
                          |       |                                                               |               +-----------------566.0-|       +-------euGI
                          |       |                                                               |               |                       |
                          |       |                                                       +-340.0-|               |                       +---------------euBH
                          |       |                                                       |       |               |
                          |       |                                                       |       |               |                               +-------euAX
                          |       |                                                       |       |       +-222.0-|               +---------984.0-|
                          |       |                                                       |       |       |       |               |               +-------euIS
                          |       |                                                       |       |       |       |       +-220.0-|
                          |       |                                                       |       |       |       |       |       |               +-------euEG
                  +--32.0-|       |                                                       |       |       |       |       |       |       +-263.0-|
                  |       |       |                                                       |       |       |       |       |       +-132.0-|       +-------euDD
                  |       |       +--------------------------------------------------93.0-|       +-203.0-|       +-151.0-|               |
                  |       |       |                                                       |               |               |               +---------------euBU
                  |       |       |                                                       |               |               |
                  |       |       |                                                       |               |               |                       +-------euIB
                  |       |       |                                                       |               |               +-----------------905.0-|
                  |       |       |                                                       |               |                                       +-------euFT
                  |       |       |                                                       |               |
                  |       |       |                                                       |               +-----------------------------------------------euBN
                  |       |       |                                                       |
                  |       |       |                                                       +---------------------------------------------------------------euBE
                  |       |       |
                  |       |       |                                                                                                               +-------euDV
                  |       |       |                                                                                                       +-253.0-|
                  |       |       |                                                                       +--------------------------26.0-|       +-------euGP
                  |       |       |                                                                       |                               |
                  |       |       |                                                                       |                               +---------------euEI
                  |       |       |                                                                       |
                  |       |       |                                                                       |                                       +-------euEL
                  |       |       |                                                                       |                       +---------549.0-|
                  |       |       |                                                                       |                       |               +-------euAC
                  |       |       +------------------------------------------------------------------1.00-|               +-400.0-|
                  |       |       |                                                                       |               |       |               +-------euFU
                  |       |       |                                                                       |               |       |       +-762.0-|
                  |       |       |                                                                       |               |       +-987.0-|       +-------euCZ
                  |       |       |                                                                       |       +-116.0-|               |
                  |       |       |                                                                       |       |       |               +---------------euCV
                  |       |       |                                                                       |       |       |
                  |       |       |                                                                       |       |       |                       +-------euEV
                  |       |       |                                                                       +--4.00-|       +-----------------998.0-|
                  |       |       |                                                                               |                               +-------euEZ
                  |       |       |                                                                               |
                  |       |       |                                                                               |                               +-------euAG
                  |       |       |                                                                               +--------------------------86.0-|
                  |       |       |                                                                                                               +-------euAK
                  |       |       |
                  |       |       |                                                                                                               +-------euBC
                  |       |       |                                                                                                       +-289.0-|
                  |       |       |                                                                                                       |       +-------euFH
                  |       |       |                                                                                               +-141.0-|
                  |       |       |                                                                                               |       |       +-------euIH
                  |       |       |                                                                                               |       +-374.0-|
                  |       |       +------------------------------------------------------------------------------------------8.00-|               +-------euHA
                  |       |       |                                                                                               |
                  |       |       |                                                                                               |               +-------euHJ
                  |       |       |                                                                                               +----------46.0-|
                  |       |       |                                                                                                               +-------euBI
                  |       |       |
                  |       |       |                                                                                               +-----------------------euAZ
                  |       |       |                                                                                       +--30.0-|
                  |       |       |                                                                                       |       |       +---------------euBG
                  |       |       |                                                                                       |       +-298.0-|
                  |       |       |                                                                                       |               |       +-------euGL
                  |       |       |                                                                                       |               +-910.0-|
                  |       |       +----------------------------------------------------------------------------------1.00-|                       +-------euIK
                  |       |       |                                                                                       |
                  |       |       |                                                                                       |               +---------------euID
                  |       |       |                                                                                       |       +-991.0-|
                  |       |       |                                                                                       |       |       |       +-------euGN
                  |       |       |                                                                                       +-155.0-|       +-667.0-|
                  |       |       |                                                                                               |               +-------euFA
          +-185.0-|       |       |                                                                                               |
          |       |       |       |                                                                                               +-----------------------euBW
          |       |       |       |
          |       |       |       |                                                                                                               +-------euGG
          |       |       |       |                                                                                       +-----------------243.0-|
          |       |       |       |                                                                                       |                       +-------euAU
          |       |       |       |                                                                                       |
          |       |       |       |                                                                                       |                       +-------euCX
          |       |       |       +----------------------------------------------------------------------------------1.00-|               +-164.0-|
          |       |       |       |                                                                                       |       +--64.0-|       +-------euFJ
          |       |       |       |                                                                                       |       |       |
          |       |       |       |                                                                                       |       |       +---------------euGO
          |       |       |       |                                                                                       +--12.0-|
          |       |       |       |                                                                                               |               +-------euGJ
          |       |       |       |                                                                                               |       +-762.0-|
          |       |       |       |                                                                                               +-964.0-|       +-------euAJ
          |       |       |       |                                                                                                       |
          |       |       |       |                                                                                                       +---------------euAI
          |       |       |       |
          |       |       |       |                                                                                                               +-------euBM
          |       |       |       |                                                                                                       +-199.0-|
          |       |       |       |                                                                                                       |       +-------euXq
          |       |       |       |                                                                                               +--29.0-|
          |       |       |       |                                                                                               |       |       +-------euCY
          |       |       |       |                                                                                               |       +-313.0-|
          |       |       |       +------------------------------------------------------------------------------------------2.00-|               +-------euAA
          |       |       |       |                                                                                               |
          |       |       |       |                                                                                               |               +-------euAS
          |       |       |       |                                                                                               |       +--1000-|
          |       |       |       |                                                                                               +--88.0-|       +-------euBF
          |       |       |       |                                                                                                       |
          |       |       |       |                                                                                                       +---------------euXi
  +-------|       |       |       |
  |       |       |       |       |                                                                                                               +-------euHP
  |       |       |       |       |                                                                                                       +-220.0-|
  |       |       |       |       |                                                                                                       |       +-------euFF
  |       |       |       |       +--------------------------------------------------------------------------------------------------7.00-|
  |       |       |       |       |                                                                                                       |       +-------euHD
  |       |       |       |       |                                                                                                       +-229.0-|
  |       |       |       |       |                                                                                                               +-------euII
  |       |       |       |       |
  |       |       |       |       |                                                                                                               +-------euBZ
  |       |       |       |       |                                                                                                       +-137.0-|
  |       |       |       |       |                                                                                                       |       +-------euHU
  |       |       |       |       +--------------------------------------------------------------------------------------------------17.0-|
  |       |       |       |       |                                                                                                       |       +-------euCU
  |       |       |       |       |                                                                                                       +-242.0-|
  |       |       |       |       |                                                                                                               +-------euEU
  |       |       |       |       |
  |       |       |       |       |                                                                                                               +-------euAR
  |       |       |       |       +----------------------------------------------------------------------------------------------------------55.0-|
  |       |       |       |                                                                                                                       +-------euES
  |       |       |       |
  |       |       |       |                                                                                                                       +-------euFV
  |       |       |       |                                                                                                               +-579.0-|
  |       |       |       |                                                                                                               |       +-------euBJ
  |       |       |       +----------------------------------------------------------------------------------------------------------35.0-|
  |       |       |                                                                                                                       |       +-------euEC
  |       |       |                                                                                                                       +-165.0-|
  |       |       |                                                                                                                               +-------euBK
  |       |       |
  |       |       |                                                                                                                               +-------euCW
  |       |       +-------------------------------------------------------------------------------------------------------------------------247.0-|
  |       |                                                                                                                                       +-------euBS
  |       |
  |       +-----------------------------------------------------------------------------------------------------------------------------------------------euEY
  |
  +-------------------------------------------------------------------------------------------------------------------------------------------------------euBO

*************************************************
*Outfile HK sequences where GTR model was used*
*************************************************
                                                                                                                                                                                  +-------euCS
                                          +---------------------------------------------------------------------------------------------------------------------------------101.0-|
                                          |                                                                                                                                       +-------euCO
                                          |
                                          |                                                                                                                                       +-------euHC
                                          |                                                                                                                               +-617.0-|
                                          |                                                                                                                               |       +-------euHB
                                          |                                                                                                                       +-762.0-|
                                          |                                                                                                                       |       |       +-------euHG
                                          |                                                                                                               +-890.0-|       +-519.0-|
                                          |                                                                                                               |       |               +-------euHH
                                          |                                                                                                       +-997.0-|       |
                                          |                                                                                                       |       |       +-----------------------euHF
                                          |                                                                                                       |       |
                                          +--------------------------------------------------------------------------------------------------11.0-|       +-------------------------------euHJ
                                          |                                                                                                       |
                                          |                                                                                                       |                               +-------euGU
                                          |                                                                                                       +-------------------------134.0-|
                                          |                                                                                                                                       +-------euFN
                                          |
                                          |                                                                                                                                       +-------euFE
                                          |                                                                                                                               +--1000-|
                                          |                                                                                                                       +-225.0-|       +-------euFP
                                          |                                                                                                                       |       |
                                          +------------------------------------------------------------------------------------------------------------------4.00-|       +---------------euGF
                                          |                                                                                                                       |
                                          |                                                                                                                       |       +---------------euEM
                                          |                                                                                                                       +-197.0-|
                                          |                                                                                                                               |       +-------euGB
                                          |                                                                                                                               +--1000-|
                                          |                                                                                                                                       +-------euGE
                                          |
                                          |                                                                                                                                       +-------euGI
                                          |               +-----------------------------------------------------------------------------------------------------------------101.0-|
                                          |               |                                                                                                                       +-------euGK
                                          |               |
                                          |               |                                                                                                                       +-------aiJD
                                          |               |                                                                       +-----------------------------------------482.0-|
                                          |               |                                                                       |                                               +-------aiJG
                                          |               |                                                                       |
                                          |               |                                                                       |                                               +-------aiIZ
                                          |               |                                                                       |                                       +--1000-|
                                          |               |                                                                       |                               +-360.0-|       +-------aiIY
                                          |               |                                                                       |                               |       |
                                          |               |                                                                       |                       +-147.0-|       +---------------aiIX
                                          |               |                                                               +-756.0-|                       |       |
                                          |               |                                                               |       |                       |       |       +---------------aiJH
                                          |               |                                                               |       |                       |       +-358.0-|
                                          |               |                                                               |       |               +-173.0-|               |       +-------aiJL
                                          |               |                                                               |       |               |       |               +-465.0-|
                                          |               |                                                               |       |               |       |                       +-------aiJF
                                          |               |                                                               |       |       +-298.0-|       |
                                          |               |                                                               |       |       |       |       +-------------------------------aiIW
                                          |               |                                                               |       |       |       |
                                          |               |                                                               |       +-183.0-|       +---------------------------------------aiJI
                                          |               |                                                       +-170.0-|               |
                                          |               |                                                       |       |               |                                       +-------aiJE
                                          |               |                                                       |       |               +---------------------------------276.0-|
                                          |               |                                                       |       |                                                       +-------aiJJ
                                          |               |                                                       |       |
                                          |               |                                                       |       |                                                       +-------aiKA
                                          |               |                                                       |       |                                               +-457.0-|
                                          |               |                                                       |       |                                       +-494.0-|       +-------aiJZ
                                          |               |                       +-------------------------335.0-|       |                                       |       |
                                          |               |                       |                               |       |                               +-284.0-|       +---------------aiKB
                                          |               |                       |                               |       |                               |       |
                                          |               |                       |                               |       +-------------------------170.0-|       +-----------------------aiJY
                                          |               |                       |                               |                                       |
                                          |               |                       |                               |                                       |                       +-------aiKE
                                          |               |                       |                               |                                       +-----------------501.0-|
                                          |               |                       |                               |                                                               +-------aiXk
                                          |               |                       |                               |
                                          |               |                       |                               +-----------------------------------------------------------------------aiXb
                                          |               |                       |
                                          |               |                       |                                                                                               +-------eaBF
                                          |               |                       |                                                                                       +-438.0-|
                                          |               |                       |                                                                                       |       +-------eaBP
                                          |               |                       |                               +--------------------------------------------------23.0-|
                                          |               |                       |                               |                                                       |       +-------euYc
                                          |               |                       |                               |                                                       +-383.0-|
                                          |               |                       |                               |                                                               +-------eaBM
                                          |               |                       |                               |
                                          |               |                       |                               |                                                               +-------eaBZ
                                          |               |                       |                               |                                               +---------250.0-|
                                          |               |                       |                               |                                               |               +-------eaBW
                                          |               |                       |                               |                                       +--33.0-|
                                          |               |                       |                               |                                       |       |               +-------eaCC
                                          |               |                       |                               |                                       |       |       +-995.0-|
                                          |               |                       |                               |                                       |       +-545.0-|       +-------eaBV
                                          |               |                       |                               |               +------------------2.00-|               |
                                          |               |                       |                               |               |                       |               +---------------eaBX
                                          |               |                       |                               |               |                       |
                                          |               |                       |                               |               |                       |                       +-------eaBU
                                          |               |                       |                               |               |                       |               +-309.0-|
                                          |               |                       |                       +--73.0-|               |                       +----------50.0-|       +-------euYd
                                          |               |                       |                       |       |               |                                       |
                                          |               |                       |                       |       |               |                                       +---------------eaBI
                                          |               |                       |                       |       |               |
                                          |               |                       |                       |       |               |                                               +-------eaBO
                                          |               |                       |                       |       |               |                                       +-263.0-|
                                          |               |                       |                       |       |               +----------------------------------91.0-|       +-------eaBN
                                          |               |                       |                       |       |       +--1.00-|                                       |
                                          |               |                       |                       |       |       |       |                                       +---------------eaCW
                                          |       +--28.0-|                       |                       |       |       |       |
                                          |       |       |                       |                       |       |       |       |                               +-----------------------eaBJ
                                          |       |       |                       |                       |       |       |       |                               |
                                          |       |       |                       |                       |       |       |       |                       +-471.0-|               +-------eaBQ
                                          |       |       |                       |                       |       |       |       |                       |       |       +--1000-|
                                          |       |       |                       |                       |       |       |       |                       |       +-612.0-|       +-------eaBL
                                          |       |       |                       |                       |       |       |       |               +-296.0-|               |
                                          |       |       |                       |                       |       |       |       |               |       |               +---------------eaCD
                                          |       |       |                       |                       |       |       |       |       +-157.0-|       |
                                          |       |       |                       |                       |       +--2.00-|       |       |       |       +-------------------------------eaBK
                                          |       |       |               +-534.0-|                       |               |       +--8.00-|       |
                                          |       |       |               |       |               +-146.0-|               |               |       +---------------------------------------eaCB
                                          |       |       |               |       |               |       |               |               |
                                          |       |       |               |       |               |       |               |               +-----------------------------------------------euXf
                                          |       |       |               |       |               |       |               |
                                          |       |       |               |       |               |       |               |                                               +---------------eaXe
                                          |       |       |               |       |               |       |               |                                       +--69.0-|
                                          |       |       |               |       |               |       |               |                                       |       |       +-------Xs
                                          |       |       |               |       |               |       |               |                                       |       +-468.0-|
                                          |       |       |               |       |               |       |               |                                       |               +-------eaXc
                                          |       |       |               |       |               |       |               +----------------------------------2.00-|
                                          |       |       |               |       |               |       |                                                       |               +-------eaBT
                                          |       |       |               |       |               |       |                                                       |       +-135.0-|
                                          |       |       |               |       |               |       |                                                       |       |       +-------eaXh
                                          |       |       |               |       |               |       |                                                       +--17.0-|
                                          |       |       |               |       |               |       |                                                               |       +-------eaCA
                                          |       |       |               |       |               |       |                                                               +-224.0-|
                                          |       |       |               |       |               |       |                                                                       +-------eaBY
                                          |       |       |               |       |               |       |
                                          |       |       |               |       |               |       |                                                                       +-------eaIV
                                          |       |       |               |       |               |       |                                                               +-399.0-|
                                          |       |       |               |       |               |       +---------------------------------------------------------185.0-|       +-------eaBS
                                          |       |       |               |       |               |                                                                       |
                                          |       |       |               |       |               |                                                                       +---------------eaGZ
                                          |       |       |               |       |               |
                                          |       |       |               |       |               |                                                                               +-------maAI
                                          |       |       |               |       |               |                                                                       +-942.0-|
                                          |       |       |               |       |               |                                                                       |       +-------maAT
                                          |       |       |               |       |               |                                                               +-861.0-|
                                          |       |       |               |       |               |                                                               |       |       +-------maAZ
                                          |       |       |               |       |               |                                                               |       +-932.0-|
                                          |       |       |               |       |       +-148.0-|                                                               |               +-------maAM
                                          |       |       |               |       |       |       |                                       +------------------83.0-|
                                          |       |       |               |       |       |       |                                       |                       |               +-------maAR
                                          |       |       |               |       |       |       |                                       |                       |       +-996.0-|
                                          |       |       |               |       |       |       |                                       |                       |       |       +-------maAE
                                          |       |       |               |       |       |       |                                       |                       +-442.0-|
                                          |       |       |       +-565.0-|       |       |       |                                       |                               |       +-------maAV
                                          |       |       |       |       |       |       |       |                                       |                               +-996.0-|
                                          |       |       |       |       |       |       |       |                                       |                                       +-------maAD
                                          |       |       |       |       |       |       |       |                                       |
                                          |       |       |       |       |       |       |       |                               +--20.0-|                               +---------------maAF
                                          |       |       |       |       |       |       |       |                               |       |                       +-358.0-|
                                          |       |       |       |       |       |       |       |                               |       |                       |       |       +-------maAG
                                          |       |       |       |       |       |       |       |                               |       |               +-141.0-|       +-660.0-|
                                          |       |       |       |       |       |       |       |                               |       |               |       |               +-------maAL
                                          |       |       |       |       |       |       |       |                               |       |               |       |
                                          |       |       |       |       |       |       |       |                               |       |       +--68.0-|       +-----------------------maAQ
                                          |       |       |       |       |       |       |       |                               |       |       |       |
                                          +--28.0-|       |       |       |       |       |       |                               |       |       |       |                       +-------maAP
                                          |       |       |       |       |       |       |       |                               |       |       |       +-----------------971.0-|
                                          |       |       |       |       |       +-589.0-|       |                               |       +--24.0-|                               +-------maAN
                                          |       |       |       |       |               |       |                               |               |
                                          |       |       |       |       |               |       |                       +-217.0-|               |       +-------------------------------maAO
                                          |       |       |       |       |               |       |                       |       |               |       |
                                          |       |       |       |       |               |       |                       |       |               +-293.0-|                       +-------maAS
                                          |       |       |       |       |               |       |                       |       |                       |       +---------642.0-|
                                          |       |       |       |       |               |       |                       |       |                       |       |               +-------maAW
                                          |       |       |       |       |               |       |                       |       |                       +-438.0-|
                                          |       |       |       |       |               |       |                       |       |                               |               +-------maAJ
                                          |       |       +--78.0-|       |               |       |                       |       |                               |       +-602.0-|
                                          |       |               |       |               |       +-----------------974.0-|       |                               +--1000-|       +-------maAK
                                          |       |               |       |               |                               |       |                                       |
                                          |       |               |       |               |                               |       |                                       +---------------maAU
                                          |       |               |       |               |                               |       |
                                          |       |               |       |               |                               |       |                                               +-------maAY
                                          |       |               |       |               |                               |       +-----------------------------------------470.0-|
                                          |       |               |       |               |                               |                                                       +-------maAH
                                          |       |               |       |               |                               |
                                          |       |               |       |               |                               +---------------------------------------------------------------maAX
                                          |       |               |       |               |
                                          |       |               |       |               +-----------------------------------------------------------------------------------------------eaBR
                                          |       |               |       |
                                          |       |               |       |                                                                                                       +-------euJP
                                          |       |               |       +-------------------------------------------------------------------------------------------------320.0-|
                                          |       |               |                                                                                                               +-------aiXj
                                          |       |               |
                                          |       |               |                                                                                               +-----------------------Xm
                                          |       |               |                                                                                               |
                                          |       |               |                                                                                       +-565.0-|               +-------euHI
                                          |       |               |                                                                                       |       |       +-936.0-|
                                          |       |               |                                                                                       |       +-321.0-|       +-------euHD
                                          |       |               |                                                                               +-621.0-|               |
                                          |       |               |                                                                               |       |               +---------------euBG
                                          |       |               +-------------------------------------------------------------------------302.0-|       |
                                          |       |                                                                                               |       +-------------------------------euHE
                                          |       |                                                                                               |
                                          |       |                                                                                               +---------------------------------------euKG
                                          |       |
                                          |       |                                                                                                                               +-------euCQ
                                          |       |                                                                                                                       +-920.0-|
                                          |       |                                                                                                               +-986.0-|       +-------euCP
                                          |       |                                                                                                               |       |
                                          |       |                                                                                               +---------855.0-|       +---------------euCH
                                          |       |                                                                                               |               |
                                          |       |                                                                                               |               +-----------------------euCK
                                          |       |                                                                                               |
                                          |       |                                                                                               |                               +-------euCG
                                          |       |                                                                                               |                       +--1000-|
                                          |       |                                                                                       +-256.0-|                       |       +-------euCL
                                          |       |                                                                                       |       |               +-353.0-|
                                          |       |                                                                                       |       |               |       |       +-------euCM
                                          |       |                                                                                       |       |       +-264.0-|       +-985.0-|
                                          |       |                                                                                       |       |       |       |               +-------euCT
                                          |       |                                                                                       |       |       |       |
                                          |       |                                                                               +-626.0-|       +-159.0-|       +-----------------------euCF
                                          |       |                                                                               |       |               |
                                          |       |                                                                               |       |               |                       +-------euCU
                                          |       |                                                                               |       |               +-----------------441.0-|
                                          |       +-------------------------------------------------------------------------177.0-|       |                                       +-------euCN
                                          |                                                                                       |       |
                                          |                                                                                       |       +-----------------------------------------------euCJ
                                          |                                                                                       |
                                          |                                                                                       |                                               +-------euCE
                                          |                                                                                       +-----------------------------------------338.0-|
                                          |                                                                                                                                       +-------euCR
                                          |
                                          |                                                                                                                                       +-------euFX
                                          |                                                                                                                               +-349.0-|
                                          |                                                                       +--------------------------------------------------89.0-|       +-------euFI
                                          |                                                                       |                                                       |
                                          |                                                                       |                                                       +---------------euHK
                                          |                                                                       |
                                          |                                                                       |               +-------------------------------------------------------euMF
                                          |                                                                       |               |
                                          |                                                                       |               |                                               +-------afbMB
                                          |                                                                       |               |                                       +-473.0-|
                                          |                                                                       |               |                               +-322.0-|       +-------afbLZ
                                          |                                                                       |               |                               |       |
                                          +------------------------------------------------------------------9.00-|               |                       +--53.0-|       +---------------afbLX
                                          |                                                                       |               |                       |       |
                                          |                                                                       |       +-676.0-|                       |       |               +-------afbLY
                                          |                                                                       |       |       |               +--72.0-|       +---------222.0-|
                                          |                                                                       |       |       |               |       |                       +-------afbKZ
                                          |                                                                       |       |       |               |       |
                                          |                                                                       |       |       |               |       |                       +-------afbLW
                                          |                                                                       |       |       |       +-431.0-|       +-----------------408.0-|
                                          |                                                                       |       |       |       |       |                               +-------afbLA
                                          |                                                                       |       |       |       |       |
                                          |                                                                       +--63.0-|       |       |       |                               +-------afbMC
                                          |                                                                               |       +--1000-|       |                       +-846.0-|
                                          |                                                                               |               |       +-----------------325.0-|       +-------afbLB
                                          |                                                                               |               |                               |
                                          |                                                                               |               |                               +---------------afbMA
                                          |                                                                               |               |
                                          |                                                                               |               +-----------------------------------------------afXt
                                          |                                                                               |
                                          |                                                                               |                                                       +-------euLD
                                          |                                                                               +-------------------------------------------------958.0-|
                                          |                                                                                                                                       +-------euMD
                                          |
                                          |                                                                                                                               +---------------euKD
                                          |                                                                                                                       +--93.0-|
                                          |                                                                                                                       |       |       +-------afaBA
                                          |                                                                                                                       |       +--1000-|
                                          |                               +----------------------------------------------------------------------------------9.00-|               +-------afaBB
                                          |                               |                                                                                       |
                                          |                               |                                                                                       |               +-------afaMZ
                                          |                               |                                                                                       +---------310.0-|
                                          |                               |                                                                                                       +-------afaHA
                                          |                               |
                                          |                               |                                                                                                       +-------afaLN
                                          |                               |                                                                               +------------------1000-|
                                          |                               |                                                                               |                       +-------afaLO
                                          |                               |                                                                               |
                                          |                               |                                                                       +-639.0-|                       +-------afaNH
                                          |                               |                                                                       |       |               +-422.0-|
                                          |                               |                                                                       |       |       +-234.0-|       +-------afaKV
                                          |                               |                                                                       |       |       |       |
                                          |                               |                                                                       |       +-455.0-|       +---------------afaMX
                                          |                               |                                                                       |               |
                                          |                               |                                                                       |               |               +-------afaND
                                          |                               |                                       +--------------------------25.0-|               +---------549.0-|
                                          |                               |                                       |                               |                               +-------afaLQ
                                          |                               |                                       |                               |
                                          |                               |                                       |                               |                               +-------afaKY
                                          |                               |                                       |                               |                       +-811.0-|
                                          |                               |                                       |                               |               +-288.0-|       +-------afaMS
                                          |                               |                                       |                               |               |       |
                                          |                               |                                       |                               |               |       +---------------afaNB
                                          |                               |                                       |                               +---------152.0-|
                                          |                               |                                       |                                               |               +-------afaKU
                                          |                               |                                       |                                               |       +--1000-|
                                          |                               |                                       |                                               |       |       +-------afaLF
                                          |                               |                                       |                                               +-926.0-|
                                          |                               |                                       |                                                       |       +-------afaKQ
                                          |                               |                                       |                                                       +--1000-|
                                          |                               |                                       |                                                               +-------afaLG
                                          |                               |                                       |
                                          |                               |                                       |                                                               +-------afaLM
                                          |                               |                                       |                                                       +-306.0-|
                                          |                               |                                       |                                                       |       +-------afaLI
                                          |                               |                               +--3.00-|                                               +-368.0-|
                                          |                               |                               |       |                                               |       |       +-------afaLR
                                          |                               |                               |       |                                               |       +-791.0-|
                                          |                               |                               |       |                                       +-477.0-|               +-------afaLT
                                          |                               |                               |       |                                       |       |
                                          |                               |                               |       |                                       |       |               +-------afaLH
                                          |                               |                               |       |                               +-192.0-|       +---------477.0-|
                                          |                               |                               |       |                               |       |                       +-------afaLV
                                          |                               |                               |       |                               |       |
                                          |                               |                               |       |                       +-139.0-|       +-------------------------------afaLK
                                          |                               |                               |       |                       |       |
                                          |                               |                               |       |                       |       |                               +-------afaKR
                                          |                               |                               |       |               +-321.0-|       +-------------------------353.0-|
                                          |                               |                               |       |               |       |                                       +-------afaKT
                                          |                               |                               |       |               |       |
                                          |                               |                               |       |       +-200.0-|       +-----------------------------------------------afaLL
                                          |                               |                       +--21.0-|       |       |       |
                                          |                               |                       |       |       |       |       |                                       +---------------afaLU
                                          |                               |                       |       |       |       |       +---------------------------------547.0-|
                                          |                               |                       |       |       |       |                                               |       +-------afaMO
                                          |                               |                       |       |       +--35.0-|                                               +-687.0-|
                                          |                               |                       |       |               |                                                       +-------afaLP
                                          |                               |                       |       |               |
                                          |                               |                       |       |               |                                                       +-------afaMW
                                          |                               |                       |       |               |                                               +-444.0-|
                                          |                               |                       |       |               +-----------------------------------------102.0-|       +-------afaNI
                                          |                               |                       |       |                                                               |
                                          +--------------------------9.00-|                       |       |                                                               +---------------afaNJ
                                          |                               |                       |       |
                                          |                               |                       |       |                                                                       +-------afaNC
                                          |                               |                       |       |                                                               +-281.0-|
                                          |                               |                       |       |                                                               |       +-------afaKW
                                          |                               |                       |       +----------------------------------------------------------46.0-|
                                          |                               |                       |                                                                       |       +-------afaMV
                                          |                               |                       |                                                                       +-427.0-|
                                          |                               |                       |                                                                               +-------afaKX
                                          |                               |               +--42.0-|
                                          |                               |               |       |                                                                               +-------afaNG
                                          |                               |               |       |                                               +-------------------------909.0-|
                                          |                               |               |       |                                               |                               +-------afaMT
                                          |                               |               |       |                                               |
                                          |                               |               |       |                                       +-557.0-|                               +-------afaLJ
                                          |                               |               |       |                                       |       |       +-----------------494.0-|
                                          |                               |               |       |                                       |       |       |                       +-------afaLS
                                          |                               |               |       |                                       |       |       |
                                          |                               |               |       |                                       |       +-559.0-|                       +-------afaMM
                                          |                               |               |       |                                       |               |               +-997.0-|
                                          |                               |               |       |                               +-200.0-|               |       +-712.0-|       +-------afaMI
                                          |                               |               |       |                               |       |               |       |       |
                                          |                               |               |       |                               |       |               +-575.0-|       +---------------afaMK
                                          |                               |               |       |                               |       |                       |
                                          |                               |               |       |                               |       |                       +-----------------------afaMU
                                          |                               |       +--11.0-|       |                       +--31.0-|       |
                                          |                               |       |       |       |                       |       |       +-----------------------------------------------afaNA
                                          |                               |       |       |       |                       |       |
                                          |                               |       |       |       |                       |       |                                               +-------afaNK
                                          |                               |       |       |       |                       |       |                                       +-978.0-|
                                          |                               |       |       |       +------------------29.0-|       +---------------------------------649.0-|       +-------afaMR
                                          |                               |       |       |                               |                                               |
                                          |                               |       |       |                               |                                               +---------------afaMN
                                          |                               |       |       |                               |
                                          |                               |       |       |                               |                                                       +-------afaNE
                                          |                               |       |       |                               |                                               +-660.0-|
                                          |                               |       |       |                               +-----------------------------------------353.0-|       +-------afaNL
                                          |                               |       |       |                                                                               |
                                          |                               |       |       |                                                                               +---------------afaKS
                                          |                               |       |       |
                                          |                               |       |       |                                                                                       +-------afaIB
                                          |                               |       |       +---------------------------------------------------------------------------------299.0-|
                                          |                               |       |                                                                                               +-------afaMQ
                                          |                               |       |
                                          |                               |       |                                                                               +-----------------------afaNR
                                          |                               |       |                                                                               |
                                          |                               |       |                                                       +-----------------531.0-|               +-------afXa
                                          |                               |       |                                                       |                       |       +--1000-|
                                          |                               |       |                                                       |                       +-718.0-|       +-------afaHQ
                                          |                               |       |                                                       |                               |
                                          |                               |       |                                                       |                               +---------------afaNS
                                          |                               |       |                                                       |
                                  +--1.00-|                               |       |                                                       |                       +-----------------------afaOB
                                  |       |                               |       |                                                       |                       |
                                  |       |                               |       |                                                       |               +--85.0-|               +-------afaIH
                                  |       |                               |       |                                                       |               |       |       +-997.0-|
                                  |       |                               |       |                                                       |               |       +-324.0-|       +-------afaHZ
                                  |       |                               |       |                                                       |               |               |
                                  |       |                               |       |                                                       |               |               +---------------afaNP
                                  |       |                               |       |                                                       |               |
                                  |       |                               |       |                                                       |               |                       +-------afaNQ
                                  |       |                               |       |                                                       |               |               +-188.0-|
                                  |       |                               |       |                                                       |               |               |       +-------afaIE
                                  |       |                               |       |                                                       |       +--1.00-|----------20.0-|
                                  |       |                               |       |                                                       |       |       |               |       +-------afaIG
                                  |       |                               +--32.0-|                                               +--63.0-|       |       |               +-370.0-|
                                  |       |                                       |                                               |       |       |       |                       +-------afaIF
                                  |       |                                       |                                               |       |       |       |
                                  |       |                                       |                                               |       |       |       |                       +-------afaOA
                                  |       |                                       |                                               |       |       |       |               +--1000-|
                                  |       |                                       |                                               |       |       |       |       +-199.0-|       +-------afaNZ
                                  |       |                                       |                                               |       |       |       |       |       |
                                  |       |                                       |                                               |       |       |       +--30.0-|       +---------------afaNY
                                  |       |                                       |                                               |       |       |               |
                                  |       |                                       |                                               |       |       |               +-----------------------afaHY
                                  |       |                                       |                                               |       |       |
                                  |       |                                       |                                               |       |       |                               +-------afXn
                                  |       |                                       |                                               |       |       |                       +-441.0-|
                                  |       |                                       |                                               |       |       |               +--1000-|       +-------afXo
                                  |       |                                       |                                               |       |       |               |       |
                                  |       |                                       |                                               |       |       |       +-535.0-|       +---------------afXp
                                  |       |                                       |                                               |       |       |       |       |
                                  |       |                                       |                                               |       |       |       |       +-----------------------afaNX
                                  |       |                                       |                                               |       |       +--9.00-|
                                  |       |                                       |                                               |       +--3.00-|       |                       +-------afaHU
                                  |       |                                       |                                               |               |       |               +-334.0-|
                                  |       |                                       |                                               |               |       +---------276.0-|       +-------afaIA
                                  |       |                                       |                                               |               |                       |
                                  |       |                                       |                                               |               |                       +---------------afaID
                                  |       |                                       |                                       +--16.0-|               |
                                  |       |                                       |                                       |       |               |                               +-------afaHT
                                  |       |                                       |                                       |       |               |                       +-219.0-|
                                  |       |                                       |                                       |       |               |                       |       +-------afaNN
                                  |       |                                       |                                       |       |               |               +--44.0-|
                                  |       |                                       |                                       |       |               |               |       |       +-------afaIC
                                  |       |                                       |                                       |       |               |               |       +-302.0-|
                                  |       |                                       |                                       |       |               +----------6.00-|               +-------afaNW
                                  |       |                                       |                                       |       |               |               |
                                  |       |                                       |                                       |       |               |               |               +-------afaMJ
                                  |       |                                       |                                       |       |               |               +----------88.0-|
                                  |       |                                       |                                       |       |               |                               +-------afaOD
                                  |       |                                       |                                       |       |               |
                                  |       |                                       |                                       |       |               |                               +-------afaNU
                                  |       |                                       |                                       |       |               |                       +-310.0-|
                                  |       |                                       |                                       |       |               |               +-154.0-|       +-------afaNM
                                  |       |                                       +----------------------------------7.00-|       |               |               |       |
                                  |       |                                                                               |       |               +----------57.0-|       +---------------afaNT
                                  |       |                                                                               |       |                               |
                                  |       |                                                                               |       |                               +-----------------------afaNV
                                  |       |                                                                               |       |
                                  |       |                                                                               |       |                                               +-------afaMH
                                  |       |                                                                               |       |                                       +-982.0-|
                                  |       |                                                                               |       |                                       |       +-------afaML
                                  |       |                                                                               |       +---------------------------------892.0-|
                                  |       |                                                                               |                                               |       +-------afaMP
                                  |       |                                                                               |                                               +-695.0-|
                                  |       |                                                                               |                                                       +-------afaNF
                                  |       |                                                                               |
                                  |       |                                                                               |                                                       +-------afaNO
                                  |       |                                                                               |                                               +-363.0-|
                                  |       |                                                                               |                                               |       +-------afaIU
                                  |       |                                                                               +------------------------------------------30.0-|
                                  |       |                                                                                                                               |       +-------afaOC
                                  |       |                                                                                                                               +-181.0-|
                                  |       |                                                                                                                                       +-------afaKF
                                  |       |
                                  |       |                                                                                                                                       +-------euFM
                                  |       |                                                                                                                               +-236.0-|
                                  |       +-------------------------------------------------------------------------------------------------------------------------108.0-|       +-------euFB
                                  |       |                                                                                                                               |
                                  |       |                                                                                                                               +---------------aiXl
                                  |       |
                                  |       |                                                                                                                                       +-------euXd
                                  |       |                                                                                                                               +--1000-|
                                  |       |                                                                                                                       +-167.0-|       +-------euGY
                                  |       |                                                                                                                       |       |
                                  |       |                                                                                                               +--18.0-|       +---------------euEX
                                  |       |                                                                                                               |       |
                                  |       |                                                                                                               |       +-----------------------euFJ
                                  |       +----------------------------------------------------------------------------------------------------------1.00-|
                                  |       |                                                                                                               |       +-----------------------euDQ
                                  |       |                                                                                                               |       |
                                  |       |                                                                                                               +--13.0-|               +-------euDX
                                  |       |                                                                                                                       |       +-139.0-|
                                  |       |                                                                                                                       +--79.0-|       +-------euXi
                                  |       |                                                                                                                               |
                                  |       |                                                                                                                               +---------------euXg
                                  |       |
                                  |       |                                                                                                                                       +-------euJU
                                  |       |                                                                                                                       +---------182.0-|
                                  |       |                                                                                                                       |               +-------euFO
                                  |       +------------------------------------------------------------------------------------------------------------------6.00-|
                                  |       |                                                                                                                       |               +-------euIQ
                                  |       |                                                                                                                       |       +-467.0-|
                                  |       |                                                                                                                       +--91.0-|       +-------euII
                                  |       |                                                                                                                               |
                                  |       |                                                                                                                               +---------------euFL
                                  |       |
                                  |       |                                                                                                                                       +-------euEE
                                  |       |                                                                                                                               +-569.0-|
                                  |       |                                                                                                                       +--95.0-|       +-------euFF
                                  |       |                                                                                                                       |       |
                                  |       +------------------------------------------------------------------------------------------------------------------27.0-|       +---------------euFG
                                  |       |                                                                                                                       |
                                  |       |                                                                                                                       |               +-------euKJ
                                  |       |                                                                                                                       +---------722.0-|
                                  |       |                                                                                                                                       +-------euDY
                                  |       |
                                  |       |                                                                                                                                       +-------euBD
                                  |       |                                                                                                                               +-137.0-|
                                  |       |                                                                                                                               |       +-------euFQ
                                  |       +--------------------------------------------------------------------------------------------------------------------------6.00-|
                                  |       |                                                                                                                               |       +-------euJW
                                  |       |                                                                                                                               +-335.0-|
                                  |       |                                                                                                                                       +-------euFD
                                  |       |
                                  |       |                                                                                                                               +---------------euFH
                                  |       |                                                                                                                       +--89.0-|
                                  |       |                                                                                                                       |       |       +-------euKC
                                  |       |                                                                                                                       |       +-261.0-|
                                  |       +------------------------------------------------------------------------------------------------------------------1.00-|               +-------euKH
                                  |       |                                                                                                                       |
                                  |       |                                                                                                                       |       +---------------euEP
                                  |       |                                                                                                                       +-104.0-|
                                  |       |                                                                                                                               |       +-------euEI
                                  |       |                                                                                                                               +-229.0-|
                                  |       |                                                                                                                                       +-------euDA
                                  |       |
                                  |       |                                                                                                                                       +-------euLC
                                  |       |                                                                                                                               +-501.0-|
                                  |       |                                                                                                                       +-645.0-|       +-------euME
                                  |       |                                                                                                                       |       |
                                  |       |                                                                                                               +-559.0-|       +---------------euKN
                                  |       |                                                                                                               |       |
                                  |       |                                                                                                       +-858.0-|       +-----------------------euKL
                                  |       |                                                                                                       |       |
                                  |       |                                                                                               +--35.0-|       +-------------------------------euKK
                                  |       |                                                                                               |       |
                                  |       |                                                                                               |       |                               +-------euBE
                                  |       +------------------------------------------------------------------------------------------2.00-|       +-------------------------245.0-|
                                  |       |                                                                                               |                                       +-------euES
                                  |       |                                                                                               |
                                  |       |                                                                                               |                               +---------------euEJ
                                  |       |                                                                                               +--------------------------91.0-|
                                  |       |                                                                                                                               |       +-------euGN
                                  |       |                                                                                                                               +-655.0-|
                                  |       |                                                                                                                                       +-------euGJ
                                  |       |
                                  |       |                                                                                                                                       +-------euDR
                                  |       |                                                                                                                               +-328.0-|
                                  |       |                                                                                                                       +--59.0-|       +-------euDC
                                  |       |                                                                                                                       |       |
                                  |       +------------------------------------------------------------------------------------------------------------------2.00-|       +---------------euXq
                                  |       |                                                                                                                       |
                                  |       |                                                                                                                       |       +---------------euGL
                                  |       |                                                                                                                       +--45.0-|
                                  |       |                                                                                                                               |       +-------euGQ
                                  |       |                                                                                                                               +-262.0-|
                                  |       |                                                                                                                                       +-------euAA
                                  |       |
                                  |       |                                                                                                                                       +-------euGX
                                  |       |                                                                                                                               +-144.0-|
                                  |       |                                                                                                                               |       +-------euKI
                                  |       +--------------------------------------------------------------------------------------------------------------------------3.00-|
                                  |       |                                                                                                                               |       +-------euCX
                                  |       |                                                                                                                               +--92.0-|
                                  |       |                                                                                                                                       +-------euER
                                  |       |
                                  |       |                                                                                                                               +---------------euDM
                                  |       |                                                                                                                       +-111.0-|
                                  |       |                                                                                                                       |       |       +-------euDD
                                  |       |                                                                                                                       |       +-501.0-|
                                  |       |                                                                                                                       |               +-------euDK
                                  |       |                                                                                                               +--6.00-|
                                  |       |                                                                                                               |       |               +-------euEC
                                  |       |                                                                                                               |       |       +-760.0-|
                                  |       |                                                                                                               |       |       |       +-------euEB
                                  |       |                                                                                                               |       +--79.0-|
                                  |       +----------------------------------------------------------------------------------------------------------1.00-|               |       +-------euDH
                                  |       |                                                                                                               |               +-784.0-|
                                  |       |                                                                                                               |                       +-------euDI
                                  |       |                                                                                                               |
                                  |       |                                                                                                               |                       +-------euED
                                  |       |                                                                                                               |               +-206.0-|
                                  |       |                                                                                                               +----------35.0-|       +-------euDZ
                                  |       |                                                                                                                               |
                          +--8.00-|       |                                                                                                                               +---------------euDV
                          |       |       |
                          |       |       |                                                                                                                                       +-------euFT
                          |       |       |                                                                                                                               +--1000-|
                          |       |       |                                                                                                                       +-102.0-|       +-------euFW
                          |       |       |                                                                                                                       |       |
                          |       |       +------------------------------------------------------------------------------------------------------------------5.00-|       +---------------euDB
                          |       |       |                                                                                                                       |
                          |       |       |                                                                                                                       |               +-------euGD
                          |       |       |                                                                                                                       +---------164.0-|
                          |       |       |                                                                                                                                       +-------euJN
                          |       |       |
                          |       |       |                                                                                                                                       +-------euIO
                          |       |       |                                                                                                                               +-135.0-|
                          |       |       +--------------------------------------------------------------------------------------------------------------------------19.0-|       +-------euFK
                          |       |       |                                                                                                                               |
                          |       |       |                                                                                                                               +---------------euDU
                          |       |       |
                          |       |       |                                                                                                                               +---------------euJA
                          |       |       |                                                                                                                       +-546.0-|
                          |       |       |                                                                                                                       |       |       +-------euJC
                          |       |       |                                                                                                               +-304.0-|       +-996.0-|
                          |       |       |                                                                                                               |       |               +-------euJK
                          |       |       |                                                                                                               |       |
                          |       |       +----------------------------------------------------------------------------------------------------------8.00-|       +-----------------------euJB
                          |       |       |                                                                                                               |
                          |       |       |                                                                                                               |                       +-------euKP
                          |       |       |                                                                                                               |               +-459.0-|
                          |       |       |                                                                                                               +----------54.0-|       +-------euDG
                          |       |       |                                                                                                                               |
                          |       |       |                                                                                                                               +---------------euDS
                          |       |       |
                          |       |       |                                                                                                                                       +-------euLE
                          |       |       |                                                                                                                               +--1000-|
                          |       |       +--------------------------------------------------------------------------------------------------------------------------57.0-|       +-------euKM
                          |       |       |                                                                                                                               |
                          |       |       |                                                                                                                               +---------------euCI
                          |       |       |
                          |       |       |                                                                                                                                       +-------euOE
                          |       |       |                                                                                                                               +-362.0-|
                          |       |       |                                                                                                                       +-270.0-|       +-------euGR
                          |       |       |                                                                                                                       |       |
                          |       |       |                                                                                                               +-669.0-|       +---------------euOF
                          |       |       |                                                                                                               |       |
                          |       |       |                                                                                                       +-386.0-|       +-----------------------euHL
                          |       |       |                                                                                                       |       |
                          |       |       +--------------------------------------------------------------------------------------------------15.0-|       +-------------------------------euBH
                          |       |       |                                                                                                       |
                          |       |       |                                                                                                       |                               +-------euFR
                          |       |       |                                                                                                       +-------------------------188.0-|
                          |       |       |                                                                                                                                       +-------euCY
                          |       |       |
                          |       |       |                                                                                                                                       +-------euJM
                          |       |       |                                                                                                                               +-872.0-|
                          |       |       |                                                                                                                       +-794.0-|       +-------euJQ
                          |       |       |                                                                                                                       |       |
                          |       |       |                                                                                                               +-522.0-|       +---------------euJV
                          |       |       |                                                                                                               |       |
                          |       |       +----------------------------------------------------------------------------------------------------------8.00-|       +-----------------------euFA
                          |       |       |                                                                                                               |
                          |       |       |                                                                                                               |                       +-------euEK
                          |       |       |                                                                                                               +------------------94.0-|
                          |       |       |                                                                                                                                       +-------euET
                          |       |       |
                          |       |       |                                                                                                                                       +-------euFV
                          |       |       |                                                                                                                               +-472.0-|
                          |       |       |                                                                                                                               |       +-------euMG
                          |       |       +--------------------------------------------------------------------------------------------------------------------------58.0-|
                          |       |       |                                                                                                                               |       +-------afaMY
                          |       |       |                                                                                                                               +-212.0-|
                          |       |       |                                                                                                                                       +-------euHX
                          |       |       |
                          |       |       |                                                                                                                                       +-------euDW
                          |       |       |                                                                                                                               +-281.0-|
                          |       |       |                                                                                                                       +-107.0-|       +-------euGH
                          |       |       |                                                                                                                       |       |
                          |       |       |                                                                                                                       |       +---------------euDL
                          |       |       |                                                                                                               +--43.0-|
                          |       |       |                                                                                                               |       |               +-------euYb
                          |       |       |                                                                                                               |       |       +-878.0-|
                          |       |       |                                                                                                               |       +-489.0-|       +-------euDO
                          |       |       +----------------------------------------------------------------------------------------------------------8.00-|               |
                          |       |       |                                                                                                               |               +---------------euGV
                          |       |       |                                                                                                               |
                          |       |       |                                                                                                               |                       +-------euGM
                          |       |       |                                                                                                               +-----------------110.0-|
                          |       |       |                                                                                                                                       +-------euDP
                          |       |       |
                          |       |       |                                                                                                                               +---------------euEN
                          |       |       |                                                                                                                       +-148.0-|
                          |       |       |                                                                                                                       |       |       +-------euEQ
                          |       |       +------------------------------------------------------------------------------------------------------------------38.0-|       +-363.0-|
                          |       |       |                                                                                                                       |               +-------euEL
                          |       |       |                                                                                                                       |
                          |       |       |                                                                                                                       +-----------------------euDN
                          |       |       |
                          |       |       |                                                                                                                       +-----------------------euIL
                          |       |       |                                                                                                               +-814.0-|
                          |       |       |                                                                                                               |       |       +---------------euIK
                  +--95.0-|       |       |                                                                                                               |       +-956.0-|
                  |       |       |       |                                                                                                       +-984.0-|               |       +-------afaIT
                  |       |       |       |                                                                                                       |       |               +-879.0-|
                  |       |       |       |                                                                                                       |       |                       +-------euIN
                  |       |       |       |                                                                                               +-334.0-|       |
                  |       |       |       |                                                                                               |       |       +-------------------------------euIR
                  |       |       |       +------------------------------------------------------------------------------------------55.0-|       |
                  |       |       |       |                                                                                               |       +---------------------------------------euEV
                  |       |       |       |                                                                                               |
                  |       |       |       |                                                                                               +-----------------------------------------------euHV
                  |       |       |       |
                  |       |       |       |                                                                                                                                       +-------euGO
                  |       |       |       |                                                                                                                       +---------223.0-|
                  |       |       |       |                                                                                                                       |               +-------euCV
                  |       |       |       +------------------------------------------------------------------------------------------------------------------13.0-|
                  |       |       |       |                                                                                                                       |               +-------euEZ
                  |       |       |       |                                                                                                                       |       +-198.0-|
                  |       |       |       |                                                                                                                       +--59.0-|       +-------euGG
                  |       |       |       |                                                                                                                               |
                  |       |       |       |                                                                                                                               +---------------euEW
                  |       |       |       |
                  |       |       |       |                                                                                                                                       +-------euJT
                  |       |       |       |                                                                                                                       +---------483.0-|
                  |       |       |       |                                                                                                                       |               +-------euGC
                  |       |       |       +------------------------------------------------------------------------------------------------------------------72.0-|
                  |       |       |       |                                                                                                                       |       +---------------euHS
                  |       |       |       |                                                                                                                       +-354.0-|
                  |       |       |       |                                                                                                                               |       +-------euHW
                  |       |       |       |                                                                                                                               +-440.0-|
                  |       |       |       |                                                                                                                                       +-------euHR
                  |       |       |       |
                  |       |       |       |                                                                                                                                       +-------euGS
                  |       |       |       |                                                                                                                               +--1000-|
                  |       |       |       |                                                                                                                               |       +-------euGW
                  |       |       |       +--------------------------------------------------------------------------------------------------------------------------45.0-|
                  |       |       |       |                                                                                                                               |       +-------euAB
                  |       |       |       |                                                                                                                               +-536.0-|
                  |       |       |       |                                                                                                                                       +-------euBC
                  |       |       |       |
                  |       |       |       |                                                                                                                                       +-------euHP
                  |       |       |       |                                                                                                                       +---------999.0-|
                  |       |       |       |                                                                                                                       |               +-------euHO
                  |       |       |       +------------------------------------------------------------------------------------------------------------------20.0-|
                  |       |       |       |                                                                                                                       |               +-------euEA
                  |       |       |       |                                                                                                                       |       +-509.0-|
                  |       |       |       |                                                                                                                       +-179.0-|       +-------euHN
                  |       |       |       |                                                                                                                               |
                  |       |       |       |                                                                                                                               +---------------euEO
                  |       |       |       |
                  |       |       |       |                                                                                                                                       +-------euJX
                  |       |       |       |                                                                                                                               +-102.0-|
          +-352.0-|       |       |       +--------------------------------------------------------------------------------------------------------------------------29.0-|       +-------euEY
          |       |       |       |       |                                                                                                                               |
          |       |       |       |       |                                                                                                                               +---------------euFY
          |       |       |       |       |
          |       |       |       |       |                                                                                                                               +---------------euIS
          |       |       |       |       |                                                                                                                       +-282.0-|
          |       |       |       |       |                                                                                                                       |       |       +-------euJS
          |       |       |       |       |                                                                                                                       |       +-737.0-|
          |       |       |       |       |                                                                                                               +-221.0-|               +-------euIP
          |       |       |       |       |                                                                                                               |       |
          |       |       |       |       |                                                                                                               |       |       +---------------euFS
          |       |       |       |       |                                                                                                               |       +-341.0-|
          |       |       |       |       +----------------------------------------------------------------------------------------------------------7.00-|               |       +-------euIM
          |       |       |       |       |                                                                                                               |               +-990.0-|
          |       |       |       |       |                                                                                                               |                       +-------euIJ
          |       |       |       |       |                                                                                                               |
          |       |       |       |       |                                                                                                               |                       +-------euFC
          |       |       |       |       |                                                                                                               +-----------------147.0-|
          |       |       |       |       |                                                                                                                                       +-------euJO
          |       |       |       |       |
          |       |       |       |       |                                                                                                                                       +-------euEG
          |       |       |       |       |                                                                                                                               +--17.0-|
          |       |       |       |       |                                                                                                                               |       +-------euEF
          |       |       |       |       +--------------------------------------------------------------------------------------------------------------------------1.00-|
          |       |       |       |       |                                                                                                                               |       +-------euEU
          |       |       |       |       |                                                                                                                               +--56.0-|
          |       |       |       |       |                                                                                                                                       +-------euJR
  +-------|       |       |       |       |
  |       |       |       |       |       |                                                                                                                                       +-------euEH
  |       |       |       |       |       |                                                                                                                               +-165.0-|
  |       |       |       |       |       |                                                                                                                               |       +-------euDF
  |       |       |       |       |       +--------------------------------------------------------------------------------------------------------------------------1.00-|
  |       |       |       |       |       |                                                                                                                               |       +-------euFU
  |       |       |       |       |       |                                                                                                                               +-207.0-|
  |       |       |       |       |       |                                                                                                                                       +-------euYa
  |       |       |       |       |       |
  |       |       |       |       |       |                                                                                                                                       +-------euDT
  |       |       |       |       |       |                                                                                                                               +-204.0-|
  |       |       |       |       |       |                                                                                                                               |       +-------euFZ
  |       |       |       |       |       +--------------------------------------------------------------------------------------------------------------------------3.00-|
  |       |       |       |       |                                                                                                                                       |       +-------euCZ
  |       |       |       |       |                                                                                                                                       +-239.0-|
  |       |       |       |       |                                                                                                                                               +-------euGA
  |       |       |       |       |
  |       |       |       |       |                                                                                                                                               +-------euKO
  |       |       |       |       +-----------------------------------------------------------------------------------------------------------------------------------------112.0-|
  |       |       |       |                                                                                                                                                       +-------euAC
  |       |       |       |
  |       |       |       |                                                                                                                                                       +-------euDJ
  |       |       |       +-------------------------------------------------------------------------------------------------------------------------------------------------414.0-|
  |       |       |                                                                                                                                                               +-------euHM
  |       |       |
  |       |       +-----------------------------------------------------------------------------------------------------------------------------------------------------------------------euGP
  |       |
  |       +-------------------------------------------------------------------------------------------------------------------------------------------------------------------------------euDE
  |
  +---------------------------------------------------------------------------------------------------------------------------------------------------------------------------------------euGT

************************
*Outfile AtpA sequences*
************************

                                                                                                                                                                                                                  +---------------GammaSBb
                                                                                                                                                                                  +-------------------------382.0-|
                                                                                                                                                                                  |                               |       +-------GammaSPc
                                                                                                                                                                                  |                               +-513.0-|
                                                                                                                                                                                  |                                       +-------GammaSSc
                                                                                                                                                                                  |
                                                                                                                                                                                  |                                       +-------GammaSPb
                                                                                                                                                                                  |                               +-852.0-|
                                                                                                                                                                                  |                       +-589.0-|       +-------GammaSPa
                                                                                                                                                                          +-245.0-|                       |       |
                                                                                                                                                                          |       |                       |       +---------------GammaSSb
                                                                                                                                                                          |       |               +-723.0-|
                                                                                                                                                                          |       |               |       |               +-------GammaSV
                                                                                                                                                                          |       |               |       |       +-342.0-|
                                                                                                                                                                          |       |       +-534.0-|       +-437.0-|       +-------GammaSH
                                                                                                                                                                  +-588.0-|       |       |       |               |
                                                                                                                                                                  |       |       |       |       |               +---------------GammaSW
                                                                                                                                                                  |       |       +-632.0-|       |
                                                                                                                                                                  |       |               |       +-------------------------------GammaSL
                                                                                                                                                          +-606.0-|       |               |
                                                                                                                                                          |       |       |               +---------------------------------------GammaSF
                                                                                                                                                          |       |       |
                                                                                                                  +---------------------------------880.0-|       |       +-------------------------------------------------------GammaSD
                                                                                                                  |                                       |       |
                                                                                                                  |                                       |       +---------------------------------------------------------------GammaSA
                                                                                                                  |                                       |
                                                                                                                  |                                       +-----------------------------------------------------------------------GammaFB
                                                                                                                  |
                                                                                                                  |                                                       +-------------------------------------------------------GammaEH
                                                                                                                  |                                                       |
                                                                                                                  |                                                       |                                               +-------GammaECb
                                                                                                                  |                                                       |                               +---------162.0-|
                                                                                                                  |                                                       |                               |               +-------GammaEAa
                                                                                                                  |                                                       |                       +-112.0-|
                                                                                                                  |                                                       |                       |       |       +---------------GammaCSb
                                                                                                                  |                                                       |                       |       +-283.0-|
                                                                                                                  |                                                       |               +-792.0-|               |       +-------GammaESb
                                                                                                                  |                                               +-503.0-|               |       |               +-818.0-|
                                                                                                                  |                                               |       |               |       |                       +-------GammaCF
                                                                                                                  |                                               |       |       +-701.0-|       |
                                                                                                                  |                                               |       |       |       |       +-------------------------------GammaECa
                                                                                                                  |                                               |       |       |       |
                                                                                                                  |                                               |       |       |       +---------------------------------------GammaYRc
                                                                                                                  |                                               |       |       |
                                                                                                                  |                                               |       |       |                                       +-------GammaSE
                                                                                                                  |                                               |       |       |                       +---------947.0-|
                                                                                                                  |                                               |       +-348.0-|                       |               +-------GammaSBa
                                                                                                                  |                                               |               |               +-665.0-|
                                                                                                                  |                                               |               |               |       |       +---------------GammaEF
                                                                                                                  |                                               |               |               |       +-849.0-|
                                                                                                                  |                                               |               |       +-471.0-|               |       +-------GammaEAb
                                                                                                                  |                                               |               |       |       |               +-313.0-|
                                                                                                                  |                                               |               |       |       |                       +-------GammaECd
                                                                                                                  |                                               |               |       |       |
                                                                                                                  |                                               |               +-519.0-|       +-------------------------------GammaCK
                                                                                                                  |                                               |                       |
                                                                                                                  |                                               |                       |                               +-------GammaKP
                                                                                                                  |                                       +-115.0-|                       |                       +-250.0-|
                                                                                                                  |                                       |       |                       +-----------------117.0-|       +-------GammaKO
                                                                                                                  |                                       |       |                                               |
                                                                                                                  |                                       |       |                                               +---------------GammaCR
                                                                                                                  |                                       |       |
                                                                                                                  |                                       |       |                                                       +-------GammaYA
                                                                                                                  |                                       |       |                               +-----------------396.0-|
                                                                                                                  |                                       |       |                               |                       +-------GammaYI
                                                                                                                  |                                       |       |                       +-931.0-|
                                                                                                                  |                                       |       |                       |       |       +-----------------------GammaYP
                                                                                                                  |                                       |       |                       |       |       |
                                                                                                                  |                                       |       |                       |       +-615.0-|               +-------GammaYRa
                                                                                                                  |                                       |       |                       |               |       +-337.0-|
                                                                                                                  |                                       |       |                       |               +-684.0-|       +-------GammaYRb
                                                                                                                  |                                       |       |               +--65.0-|                       |
                                                                                                                  |                                       |       |               |       |                       +---------------GammaYB
                                                                                                          +-479.0-|                                       |       |               |       |
                                                                                                          |       |                                       |       |               |       |                               +-------GammaEI
                                                                                                          |       |                               +-154.0-|       |               |       |                       +-430.0-|
                                                                                                          |       |                               |       |       |               |       |               +-773.0-|       +-------GammaEBa
                                                                                                          |       |                               |       |       |               |       |               |       |
                                                                                                          |       |                               |       |       +----------96.0-|       +---------223.0-|       +---------------GammaETa
                                                                                                          |       |                               |       |                       |                       |
                                                                                                          |       |                               |       |                       |                       +-----------------------GammaSG
                                                                                                          |       |                               |       |                       |
                                                                                                          |       |                               |       |                       |                                       +-------GammaECc
                                                                                                          |       |                               |       |                       |                       +---------159.0-|
                                                                                                          |       |                               |       |                       |                       |               +-------GammaBS
                                                                                                          |       |                               |       |                       +-----------------194.0-|
                                                                                                          |       |                               |       |                                               |       +---------------GammaDD
                                                                                                          |       |                               |       |                                               +-187.0-|
                                                                                                          |       |                       +-116.0-|       |                                                       |       +-------GammaRA
                                                                                                          |       |                       |       |       |                                                       +-889.0-|
                                                                                                          |       |                       |       |       |                                                               +-------GammaSSa
                                                                                                          |       |                       |       |       |
                                                                                                          |       |                       |       |       |                                                               +-------GammaPMb
                                                                                                          |       |                       |       |       +---------------------------------------------------------999.0-|
                                                                                                          |       |                       |       |                                                                       +-------GammaPPb
                                                                                                          |       |                       |       |
                                                                                                          |       |                       |       |                                                                       +-------GammaPSa
                                                                                                          |       |                       |       |                                                       +---------999.0-|
                                                                                                          |       |                       |       |                                                       |               +-------GammaPSc
                                                                                                          |       |                       |       |                                               +-871.0-|
                                                                                                          |       |               +-314.0-|       |                                               |       |       +---------------GammaPSb
                                                                                                          |       |               |       |       |                                               |       +-627.0-|
                                                                                                          |       |               |       |       +-----------------------------------------115.0-|               |       +-------GammaEBb
                                                                                                          |       |               |       |                                                       |               +-939.0-|
                                                                                                          |       |               |       |                                                       |                       +-------GammaEA
                                                                                                          |       |               |       |                                                       |
                                                                                                          |       |               |       |                                                       +-------------------------------GammaSO
                                                                                                          |       |               |       |
                                                                                                          |       |               |       |                                                                               +-------GammaPD
                                                                                                          |       |               |       |                                                                       +-966.0-|
                                                                                                          |       |       +-989.0-|       |                                                               +-999.0-|       +-------GammaPMa
                                                                                                  +-753.0-|       |       |       |       |                                                               |       |
                                                                                                  |       |       |       |       |       +---------------------------------------------------------335.0-|       +---------------GammaGA
                                                                                                  |       |       |       |       |                                                                       |
                                                                                                  |       |       |       |       |                                                                       +-----------------------GammaCH
                                                                                                  |       |       |       |       |
                                                                                                  |       |       |       |       |                                                                                       +-------GammaPRb
                                                                                                  |       |       |       |       |                                                                               +-795.0-|
                                                                                                  |       |       |       |       |                                                                       +-844.0-|       +-------GammaPAc
                                                                                                  |       |       |       |       |                                                                       |       |
                                                                                                  |       |       |       |       +-----------------------------------------------------------------837.0-|       +---------------GammaPRa
                                                                                                  |       |       |       |                                                                               |
                                                                                                  |       |       |       |                                                                               +-----------------------GammaPSe
                                                                                                  |       |       |       |
                                                                                                  |       |       |       |                                                                                               +-------GammaPAb
                                                                                                  |       |       +-406.0-|                                                                                       +--1000-|
                                                                                                  |       |               |                                                                               +-957.0-|       +-------GammaPL
                                                                                                  |       |               |                                                                               |       |
                                                                                                  |       |               |                                                                       +-807.0-|       +---------------GammaPPa
                                                                                                  |       |               |                                                                       |       |
                                                                                                  |       |               |                                                                       |       |               +-------GammaVFb
                                                                                                  |       |               |                                                                       |       +---------992.0-|
                                                                                                  |       |               |                                                                       |                       +-------GammaVS
                                                                                                  |       |               |                                                                       |
                                                                                                  |       |               |                                                               +-711.0-|                       +-------GammaVFa
                                                                                                  |       |               |                                                               |       |               +-978.0-|
                                                                                                  |       |               |                                                               |       |               |       +-------GammaASb
                                                                                                  |       |               |                                                               |       |       +-986.0-|
                                                                                          +-899.0-|       |               |                                                               |       |       |       |       +-------GammaVP
                                                                                          |       |       |               |                                                               |       |       |       +-997.0-|
                                                                                          |       |       |               +---------------------------------------------------------311.0-|       +-995.0-|               +-------GammaVAa
                                                                                          |       |       |                                                                               |               |
                                                                                          |       |       |                                                                               |               |               +-------GammaPSd
                                                                                          |       |       |                                                                               |               +---------992.0-|
                                                                                          |       |       |                                                                               |                               +-------GammaVAb
                                                                                          |       |       |                                                                               |
                                                                                          |       |       |                                                                               |                               +-------GammaPI
                                                                                          |       |       |                                                                               +-------------------------691.0-|
                                                                                          |       |       |                                                                                                               +-------GammaMSd
                                                                                          |       |       |
                                                                                          |       |       +-----------------------------------------------------------------------------------------------------------------------GammaCP
                                                                                          |       |
                                                                                          |       |                                                                                                                       +-------GammaAM
                                                                                          |       |                                                                                                       +---------996.0-|
                                                                                          |       |                                                                                                       |               +-------GammaASc
                                                                                          |       |                                                                                               +-695.0-|
                                                                                          |       |                                                                                               |       |               +-------GammaGS
                                                                                  +-661.0-|       |                                                                                               |       |       +-999.0-|
                                                                                  |       |       |                                                                                               |       +-427.0-|       +-------GammaPAd
                                                                                  |       |       |                                                                                       +-270.0-|               |
                                                                                  |       |       |                                                                                       |       |               +---------------GammaGP
                                                                                  |       |       |                                                                                       |       |
                                                                                  |       |       |                                                                                       |       |       +-----------------------GammaPT
                                                                                  |       |       |                                                                                       |       +-997.0-|
                                                                                  |       |       |                                                                                       |               |       +---------------GammaABb
                                                                                  |       |       +---------------------------------------------------------------------------------595.0-|               +-846.0-|
                                                                                  |       |                                                                                               |                       |       +-------GammaPH
                                                                                  |       |                                                                                               |                       +-400.0-|
                                                                                  |       |                                                                                               |                               +-------GammaPSf
                                                                                  |       |                                                                                               |
                                                                                  |       |                                                                                               |                               +-------GammaIL
                                                                                  |       |                                                                                               |                       +--1000-|
                                                                                  |       |                                                                                               +-----------------184.0-|       +-------GammaIB
                                                                                  |       |                                                                                                                       |
                                                                                  |       |                                                                                                                       +---------------GammaRN
                                                                                  |       |
                                                                          +-251.0-|       +---------------------------------------------------------------------------------------------------------------------------------------GammaKKa
                                                                          |       |
                                                                          |       |                                                                                                                       +-----------------------GammaMMb
                                                                          |       |                                                                                                                       |
                                                                          |       |                                                                                                               +-998.0-|               +-------GammaMP
                                                                          |       |                                                                                                               |       |       +-800.0-|
                                                                          |       |                                                                                                               |       +-955.0-|       +-------GammaMSc
                                                                          |       |                                                                                                       +-327.0-|               |
                                                                          |       |                                                                                                       |       |               +---------------GammaMSb
                                                                          |       |                                                                                                       |       |
                                                                          |       |                                                                                                       |       |               +---------------GammaRS
                                                                          |       |                                                                                                       |       +---------489.0-|
                                                                          |       |                                                                                               +--71.0-|                       |       +-------GammaMC
                                                                          |       |                                                                                               |       |                       +-885.0-|
                                                                          |       |                                                                                               |       |                               +-------GammaAR
                                                                          |       |                                                                                               |       |
                                                                          |       |                                                                                               |       |                               +-------GammaABa
                                                                          |       |                                                                                               |       |                       +--1000-|
                                                                          |       |                                                                                               |       +-----------------238.0-|       +-------GammaASa
                                                                  +-268.0-|       |                                                                                               |                               |
                                                                  |       |       +-----------------------------------------------------------------------------------------624.0-|                               +---------------GammaMG
                                                                  |       |                                                                                                       |
                                                                  |       |                                                                                                       |                                       +-------GammaHSb
                                                                  |       |                                                                                                       |                               +-924.0-|
                                                                  |       |                                                                                                       |                       +-592.0-|       +-------GammaHSa
                                                                  |       |                                                                                                       |                       |       |
                                                                  |       |                                                                                                       |               +--1000-|       +---------------GammaCSa
                                                                  |       |                                                                                                       |               |       |
                                                                  |       |                                                                                                       |               |       +-----------------------GammaHE
                                                                  |       |                                                                                                       +---------228.0-|
                                                          +-117.0-|       |                                                                                                                       |               +---------------GammaMAc
                                                          |       |       |                                                                                                                       |       +--1000-|
                                                          |       |       |                                                                                                                       |       |       |       +-------GammaMMa
                                                          |       |       |                                                                                                                       +-808.0-|       +-814.0-|
                                                          |       |       |                                                                                                                               |               +-------GammaMAb
                                                          |       |       |                                                                                                                               |
                                                          |       |       |                                                                                                                               +-----------------------GammaHJ
                                                          |       |       |
                                                          |       |       |                                                                                                                                               +-------GammaXG
                                                          |       |       +------------------------------------------------------------------------------------------------------------------------------------------1000-|
                                                          |       |                                                                                                                                                       +-------GammaXS
                                                  +-106.0-|       |
                                                  |       |       +---------------------------------------------------------------------------------------------------------------------------------------------------------------GammaHN
                                                  |       |
                                                  |       |                                                                                                                                                               +-------GammaMT
                                                  |       |                                                                                                                                       +------------------1000-|
                                                  |       |                                                                                                                                       |                       +-------GammaMA
                                                  |       |                                                                                                                                       |
                                                  |       |                                                                                                                               +-246.0-|                       +-------GammaTM
                                                  |       |                                                                                                                               |       |               +-665.0-|
                                                  |       |                                                                                                                               |       |       +-995.0-|       +-------GammaTV
                                                  |       |                                                                                                                               |       |       |       |
                                                  |       +--------------------------------------------------------------------------------------------------------------------------66.0-|       +-865.0-|       +---------------GammaTD
                                                  |                                                                                                                                       |               |
                                          +-416.0-|                                                                                                                                       |               +-----------------------GammaETb
                                          |       |                                                                                                                                       |
                                          |       |                                                                                                                                       |                               +-------GammaBN
                                          |       |                                                                                                                                       +-------------------------238.0-|
                                          |       |                                                                                                                                                                       +-------GammaESa
                                          |       |
                                          |       |                                                                                                                                                                       +-------BetaNM
                                          |       |                                                                                                                                                               +-949.0-|
                                          |       |                                                                                                                                                       +-998.0-|       +-------BetaKKb
                                  +-998.0-|       |                                                                                                                                                       |       |
                                  |       |       |                                                                                                                                               +-348.0-|       +---------------BetaLN
                                  |       |       |                                                                                                                                               |       |
                                  |       |       |                                                                                                                                       +-688.0-|       +-----------------------BetaLS
                                  |       |       |                                                                                                                                       |       |
                                  |       |       +---------------------------------------------------------------------------------------------------------------------------------486.0-|       +-------------------------------BetaDA
                                  |       |                                                                                                                                               |
                                  |       |                                                                                                                                               +---------------------------------------BetaTD
                                  |       |
                                  |       +---------------------------------------------------------------------------------------------------------------------------------------------------------------------------------------GammaCB
                                  |
                                  |                                                                                                                                                                               +---------------EpsilonAN
                                  |                                                                                                                                                                       +--1000-|
                                  |                                                                                                                                                                       |       |       +-------EpsilonAB
                                  |                                                                                                                                                                       |       +-823.0-|
                                  |                                                                                                                                       +-------------------------564.0-|               +-------EpsilonAS
                                  |                                                                                                                                       |                               |
                                  |                                                                                                                                       |                               |       +---------------EpsilonSK
                                  |                                                                                                                                       |                               +--1000-|
                                  |                                                                                                                                       |                                       |       +-------EpsilonSDa
                                  |                                                                                                                                       |                                       +-995.0-|
                                  |                                                                                                                                       |                                               +-------EpsilonCB
                                  |                                                                                                                                       |
                                  |                                                                                                                               +-429.0-|                               +-----------------------EpsilonWS
                                  |                                                                                                                               |       |                               |
                                  |                                                                                                                               |       |       +-----------------889.0-|               +-------EpsilonHCa
                                  |                                                                                                                               |       |       |                       |       +-925.0-|
                                  |                                                                                                                               |       |       |                       +-998.0-|       +-------EpsilonHPa
                          +-849.0-|                                                                                                                               |       |       |                               |
                          |       |                                                                                                                               |       |       |                               +---------------EpsilonHW
                          |       |                                                                                                                               |       |       |
                          |       |                                                                                                                               |       +-967.0-|               +-------------------------------EpsilonHBa
                          |       |                                                                                                                               |               |               |
                          |       |                                                                                                                               |               |       +-867.0-|                       +-------EpsilonHPb
                          |       |                                                                                                                               |               |       |       |       +----------1000-|
                          |       |                                                                                                                               |               |       |       |       |               +-------EpsilonHA
                          |       |                                                                                                                               |               |       |       +-994.0-|
                          |       |                                                                                                                               |               |       |               |               +-------EpsilonHS
                          |       |                                                                                                                               |               +-862.0-|               |       +-887.0-|
                          |       |                                                                                                                               |                       |               +-943.0-|       +-------EpsilonHBb
                          |       |                                                                                                                               |                       |                       |
                          |       |                                                                                                                               |                       |                       +---------------EpsilonHF
                          |       |                                                                                                                       +--1000-|                       |
                          |       |                                                                                                                       |       |                       |                               +-------EpsilonHCb
                          |       |                                                                                                                       |       |                       +-------------------------999.0-|
                          |       |                                                                                                                       |       |                                                       +-------EpsilonHH
                          |       |                                                                                                                       |       |
                          |       |                                                                                                                       |       |                                                       +-------EpsilonCH
                          |       |                                                                                                                       |       |                                               +-997.0-|
                          |       |                                                                                                                       |       |                               +---------769.0-|       +-------EpsilonCG
                          |       |                                                                                                                       |       |                               |               |
                          |       |                                                                                                                       |       |                               |               +---------------EpsilonCF
                          |       |                                                                                                                       |       |                               |
                          |       |                                                                                                                       |       |                       +-743.0-|                       +-------EpsilonCSb
                          |       |                                                                                                                       |       |                       |       |               +-999.0-|
                  +--1000-|       |                                                                                                                       |       |                       |       |       +-956.0-|       +-------EpsilonCCb
                  |       |       +-----------------------------------------------------------------------------------------------------------------472.0-|       |                       |       |       |       |
                  |       |                                                                                                                               |       |               +-537.0-|       +-483.0-|       +---------------EpsilonCCc
                  |       |                                                                                                                               |       |               |       |               |
                  |       |                                                                                                                               |       |               |       |               +-----------------------EpsilonCSa
                  |       |                                                                                                                               |       |               |       |
                  |       |                                                                                                                               |       +---------766.0-|       +---------------------------------------EpsilonSDb
                  |       |                                                                                                                               |                       |
                  |       |                                                                                                                               |                       |                                       +-------EpsilonCCa
                  |       |                                                                                                                               |                       |                               +-931.0-|
                  |       |                                                                                                                               |                       +--------------------------1000-|       +-------EpsilonCJ
                  |       |                                                                                                                               |                                                       |
                  |       |                                                                                                                               |                                                       +---------------EpsilonCU
                  |       |                                                                                                                               |
                  |       |                                                                                                                               |                                                               +-------DeltaGL
                  |       |                                                                                                                               +---------------------------------------------------------998.0-|
                  |       |                                                                                                                                                                                               +-------DeltaDAb
                  |       |
          +--1000-|       |                                                                                                                                                                                               +-------CFBFB
          |       |       |                                                                                                                                                                                       +--1000-|
          |       |       |                                                                                                                                                                                       |       +-------CFBFF
          |       |       |                                                                                                                                                                               +-954.0-|
          |       |       |                                                                                                                                                                               |       |       +-------CFBMOa
          |       |       |                                                                                                                                                                       +-783.0-|       +-998.0-|
          |       |       |                                                                                                                                                                       |       |               +-------CFBMOb
          |       |       |                                                                                                                                                               +--1000-|       |
          |       |       |                                                                                                                                                               |       |       +-----------------------CFBCS
          |       |       |                                                                                                                                                               |       |
          |       |       +---------------------------------------------------------------------------------------------------------------------------------------------------------998.0-|       +-------------------------------CFBPT
  +-------|       |                                                                                                                                                                       |
  |       |       |                                                                                                                                                                       |                               +-------CFBAI
  |       |       |                                                                                                                                                                       +-------------------------999.0-|
  |       |       |                                                                                                                                                                                                       +-------CFBDG
  |       |       |
  |       |       |                                                                                                                                                                                                       +-------GammaGN
  |       |       |                                                                                                                                                                                               +-674.0-|
  |       |       +-----------------------------------------------------------------------------------------------------------------------------------------------------------------------------------------862.0-|       +-------DeltaDP
  |       |                                                                                                                                                                                                       |
  |       |                                                                                                                                                                                                       +---------------DeltaDAa
  |       |
  |       +-----------------------------------------------------------------------------------------------------------------------------------------------------------------------------------------------------------------------GammaMSa
  |
  +-------------------------------------------------------------------------------------------------------------------------------------------------------------------------------------------------------------------------------GammaMAa

*************************
*Outfile OMPLA sequences*
*************************
                                                                                                                                                  +---------------------------------------------------------------DeltaDAa
                                                                                                                                                  |
                                                                                                                                                  |                                               +---------------GammaAR
                                                                                                                                                  |                                       +-371.0-|
                                                                                                                                                  |                                       |       |       +-------GammaXG
                                                                                                                                                  |                       +---------437.0-|       +-638.0-|
                                                                                                                                                  |                       |               |               +-------GammaXS
                                                                                                                                                  |                       |               |
                                                                                                                                                  |                       |               +-----------------------BetaLS
                                                                                                                                                  |               +-227.0-|
                                                                                                                                                  |               |       |       +-------------------------------CFBFF
                                                                                                                                                  |               |       |       |
                                                                                                                                          +--65.0-|               |       |       |                       +-------CFBFB
                                                                                                                                          |       |               |       +-425.0-|               +-264.0-|
                                                                                                                                          |       |               |               |       +-214.0-|       +-------CFBPT
                                                                                                                                          |       |               |               |       |       |
                                                                                                                                          |       |       +-136.0-|               +-289.0-|       +---------------CFBCS
                                                                                                                                          |       |       |       |                       |
                                                                                                                                          |       |       |       |                       |               +-------CFBMOb
                                                                                                                                          |       |       |       |                       +---------938.0-|
                                                                                                                                          |       |       |       |                                       +-------CFBMOa
                                                                                                                                          |       |       |       |
                                                                                                                                          |       |       |       |                                       +-------GammaTM
                                                                                                                                          |       |       |       |                               +-765.0-|
                                                                                                                                          |       +-139.0-|       +-------------------------962.0-|       +-------GammaTV
                                                                                                                                          |               |                                       |
                                                                                                                                          |               |                                       +---------------GammaESa
                                                                                                                                          |               |
                                                                                                                                          |               |                                               +-------BetaNM
                                                                                                                                  +--14.0-|               |                                       +-813.0-|
                                                                                                                                  |       |               |                               +-396.0-|       +-------BetaKKb
                                                                                                                                  |       |               |                               |       |
                                                                                                                                  |       |               +-------------------------138.0-|       +---------------BetaDA
                                                                                                                                  |       |                                               |
                                                                                                                                  |       |                                               |               +-------BetaLN
                                                                                                                                  |       |                                               +---------283.0-|
                                                                                                                                  |       |                                                               +-------GammaMC
                                                                                                                                  |       |
                                                                                                                                  |       |                                                               +-------EpsilonCCa
                                                                                                                                  |       |                                                       +-500.0-|
                                                                                                                                  |       |                                                       |       +-------EpsilonCU
                                                                                                                                  |       |                                               +-935.0-|
                                                                                                                                  |       |                                               |       |       +-------EpsilonCJ
                                                                                                                                  |       |                               +---------586.0-|       +-913.0-|
                                                                                                                                  |       |                               |               |               +-------EpsilonCSb
                                                                                                                                  |       |                               |               |
                                                                                                                                  |       |                               |               +-----------------------EpsilonCF
                                                                                                                                  |       +--------------------------78.0-|
                                                                                                                                  |                                       |                               +-------CFBAI
                                                                  +----------------------------------------------------------2.00-|                                       |       +-----------------930.0-|
                                                                  |                                                               |                                       |       |                       +-------CFBDG
                                                                  |                                                               |                                       |       |
                                                                  |                                                               |                                       +-233.0-|                       +-------EpsilonHPa
                                                                  |                                                               |                                               |               +-936.0-|
                                                                  |                                                               |                                               |       +-311.0-|       +-------EpsilonHCa
                                                                  |                                                               |                                               |       |       |
                                                                  |                                                               |                                               +-393.0-|       +---------------EpsilonHW
                                                                  |                                                               |                                                       |
                                                                  |                                                               |                                                       +-----------------------EpsilonWS
                                                                  |                                                               |
                                                                  |                                                               |                                                       +-----------------------GammaHN
                                                                  |                                                               |                                                       |
                                                                  |                                                               |                               +-----------------197.0-|               +-------EpsilonCB
                                                                  |                                                               |                               |                       |       +-971.0-|
                                                                  |                                                               |                               |                       +-459.0-|       +-------EpsilonSDa
                                                                  |                                                               |                               |                               |
                                                                  |                                                               |                               |                               +---------------GammaCB
                                                                  |                                                               |                               |
                                                                  |                                                               |                               |                                       +-------EpsilonCCc
                                                                  |                                                               |                               |                               +-580.0-|
                                                                  |                                                               +--------------------------9.00-|                       +-409.0-|       +-------EpsilonCSa
                                                                  |                                                                                               |                       |       |
                                                                  |                                                                                               |               +-212.0-|       +---------------EpsilonCCb
                                                                  |                                                                                               |               |       |
                                                                  |                                                                                               |               |       |               +-------EpsilonAB
                                                                  |                                                                                               |       +--84.0-|       +---------961.0-|
                                                                  |                                                                                               |       |       |                       +-------EpsilonAS
                                                                  |                                                                                               |       |       |
                                                                  |                                                                                               |       |       |                       +-------EpsilonCG
                                                                  |                                                                                               +--44.0-|       +-----------------374.0-|
                                                                  |                                                                                                       |                               +-------EpsilonCH
                                                                  |                                                                                                       |
                                                                  |                                                                                                       |                               +-------EpsilonSK
                                                                  |                                                                                                       |                       +-176.0-|
                                                                  |                                                                                                       +-----------------103.0-|       +-------EpsilonSDb
                                                                  |                                                                                                                               |
                                                                  |                                                                                                                               +---------------EpsilonAN
                                                                  |
                                                                  |                                                                                                                                       +-------GammaEF
                                                                  |                                                                                                                       +----------96.0-|
                                                                  |                                                                                                                       |               +-------GammaECd
                                                                  |                                                                                                               +--48.0-|
                                                                  |                                                                                                               |       |       +---------------GammaSE
                                                                  |                                                                                                               |       +-436.0-|
                                                                  |                                                                                                       +--24.0-|               |       +-------GammaCK
                                                                  |                                                                                                       |       |               +-506.0-|
                                                                  |                                                                                                       |       |                       +-------GammaEAa
                                                                  |                                                                                                       |       |
                                                                  |                                                                                                       |       +-------------------------------GammaEAb
                                                                  |                                                                                               +--49.0-|
                                                                  |                                                                                               |       |                               +-------GammaSBa
                                                                  |                                                                                               |       |       +-----------------203.0-|
                                                                  |                                                                                               |       |       |                       +-------GammaCR
                                                                  |                                                                                               |       |       |
                                                                  |                                                                                               |       +-368.0-|               +---------------GammaYRc
                                                                  |                                                                                               |               |       +-353.0-|
                                                                  |                                                                                               |               |       |       |       +-------GammaKP
                                                                  |                                                                                       +--91.0-|               +-309.0-|       +-464.0-|
                                                                  |                                                                                       |       |                       |               +-------GammaKO
                                                                  |                                                                                       |       |                       |
                                                                  |                                                                                       |       |                       +-----------------------GammaPV
                                                                  |                                                                                       |       |
                                                                  |                                                                                       |       |                                       +-------GammaECb
                                                                  |                                                                                       |       |                       +---------313.0-|
                                                                  |                                                       +-------------------------576.0-|       |                       |               +-------GammaECa
                                                                  |                                                       |                               |       +------------------80.0-|
                                                                  |                                                       |                               |                               |       +---------------GammaCF
                                                                  |                                                       |                               |                               +-226.0-|
                                                                  |                                                       |                               |                                       |       +-------GammaEH
                                                                  |                                                       |                               |                                       +-522.0-|
                                                                  |                                                       |                               |                                               +-------GammaCSb
                                                                  |                                                       |                               |
                                                                  |                                                       |                               +-------------------------------------------------------GammaESb
                                                                  |                                                       |
                                                                  |                                                       |                                                                       +---------------GammaPSa
                                                                  |                                                       |                                                               +-754.0-|
                                                                  |                                                       |                                                               |       |       +-------GammaPSb
                                                                  |                                                       |       +-------------------------------------------------392.0-|       +-823.0-|
                                                                  |                                                       |       |                                                       |               +-------GammaPSc
                                                                  |                                               +-252.0-|       |                                                       |
                                                                  |                                               |       |       |                                                       +-----------------------GammaRA
                                                                  |                                               |       |       |
                                                                  |                                               |       |       |                                                               +---------------GammaYA
                                                                  |                                               |       |       |                                                       +-195.0-|
                                                                  |                                               |       |       |                                                       |       |       +-------GammaYB
                                                                  |                                               |       |       |                                               +-584.0-|       +-152.0-|
                                                                  |                                               |       |       |                                               |       |               +-------GammaPAa
                                                                  |                                               |       |       |                                       +-369.0-|       |
                                                                  |                                               |       |       |                                       |       |       +-----------------------GammaYI
                                                                  |                                               |       |       |                                       |       |
                                                                  |                                               |       |       |                               +-486.0-|       +-------------------------------GammaYRa
                                                                  |                                               |       |       |                               |       |
                                                                  |                                               |       |       |                               |       |                               +-------GammaYP
                                                                  |                                               |       |       |                       +-107.0-|       +-------------------------182.0-|
                                                                  |                                               |       |       |                       |       |                                       +-------GammaYRb
                                                                  |                                               |       +--52.0-|                       |       |
                                                                  |                                               |               |                       |       |                               +---------------GammaEBa
                                                                  |                                               |               |                       |       +-------------------------522.0-|
                                                                  |                                               |               |               +--94.0-|                                       |       +-------GammaETa
                                                                  |                                               |               |               |       |                                       +-924.0-|
                                                          +--1.00-|                                               |               |               |       |                                               +-------GammaEI
                                                          |       |                                               |               |               |       |
                                                          |       |                                               |               |               |       |                                               +-------GammaCH
                                                          |       |                                               |               |               |       +-----------------------------------------241.0-|
                                                          |       |                                       +-511.0-|               |               |                                                       +-------GammaSSa
                                                          |       |                                       |       |               |       +--86.0-|
                                                          |       |                                       |       |               |       |       |                                                       +-------GammaPAc
                                                          |       |                                       |       |               |       |       |                                               +-822.0-|
                                                          |       |                                       |       |               |       |       |                                               |       +-------GammaPRb
                                                          |       |                                       |       |               |       |       |                                       +-338.0-|
                                                          |       |                                       |       |               |       |       |                                       |       |       +-------GammaPPb
                                                          |       |                                       |       |               |       |       |                                       |       +-686.0-|
                                                          |       |                                       |       |               |       |       +---------------------------------242.0-|               +-------GammaPMb
                                                          |       |                                       |       |               |       |                                               |
                                                          |       |                                       |       |               +--19.0-|                                               |               +-------GammaPSe
                                                          |       |                                       |       |                       |                                               +---------205.0-|
                                                          |       |                                       |       |                       |                                                               +-------GammaPRa
                                                          |       |                                       |       |                       |
                                                          |       |                                       |       |                       |                                                       +---------------GammaBS
                                                          |       |                               +-600.0-|       |                       |                                               +-709.0-|
                                                          |       |                               |       |       |                       |                                               |       |       +-------GammaDD
                                                          |       |                               |       |       |                       |                                       +-623.0-|       +-420.0-|
                                                          |       |                               |       |       |                       |                                       |       |               +-------GammaECc
                                                          |       |                               |       |       |                       +----------------------------------86.0-|       |
                                                          |       |                               |       |       |                                                               |       +-----------------------GammaSG
                                                          |       |                               |       |       |                                                               |
                                                          |       |                               |       |       |                                                               +-------------------------------GammaSO
                                                          |       |                               |       |       |
                                                          |       |                               |       |       |                                                                                       +-------GammaEBb
                                                          |       |                       +-349.0-|       |       +---------------------------------------------------------------------------------605.0-|
                                                          |       |                       |       |       |                                                                                               +-------GammaEA
                                                          |       |                       |       |       |
                                                          |       |                       |       |       |                                                                                               +-------GammaPD
                                                          |       |                       |       |       |                                                                                       +-615.0-|
                                                          |       |                       |       |       +---------------------------------------------------------------------------------771.0-|       +-------GammaPMa
                                                          |       |               +--65.0-|       |                                                                                               |
                                                          |       |               |       |       |                                                                                               +---------------GammaGA
                                                          |       |               |       |       |
                                                          |       |               |       |       |                                                                                                       +-------GammaASb
                                                          |       |               |       |       +--------------------------------------------------------------------------------------------------1000-|
                                                          |       |               |       |                                                                                                               +-------GammaVFa
                                                          |       |               |       |
                                                          |       |               |       +-----------------------------------------------------------------------------------------------------------------------GammaRS
                                                          |       |               |
                                                          |       |               |                                                                                                               +---------------GammaSPb
                                                          |       |               |                                                                                                       +-504.0-|
                                                          |       |               |                                                                                                       |       |       +-------GammaSH
                                                          |       |               |                                                                                               +-216.0-|       +-654.0-|
                                                          |       |               |                                                                                               |       |               +-------GammaSPa
                                                          |       |               |                                                                                       +-227.0-|       |
                                                          |       |               |                                                                                       |       |       +-----------------------GammaSD
                                                          |       |               |                                                                               +-154.0-|       |
                                                          |       |               |                                                                               |       |       +-------------------------------GammaSW
                                                          |       |               |                                                                               |       |
                                                          |       |               |                                                                       +-227.0-|       +---------------------------------------GammaSSb
                                                          |       |               |                                                                       |       |
                                                          |       |               |                                                                       |       |               +-------------------------------GammaSL
                                                          |       |               |                                                                       |       |               |
                                                          |       |               |                                                                       |       +----------64.0-|       +-----------------------GammaSF
                                                          |       |               |                                                                       |                       |       |
                                                          |       |               |                                                               +-251.0-|                       +-182.0-|               +-------GammaSBb
                                                          |       |               |                                                               |       |                               |       +-897.0-|
                                                          |       |               |                                                               |       |                               +-969.0-|       +-------GammaSSc
                                                          |       |               |                                                               |       |                                       |
                                                          |       |       +--19.0-|                                                       +-498.0-|       |                                       +---------------GammaSPc
                                                          |       |       |       |                                                       |       |       |
                                                          |       |       |       |                                                       |       |       +-------------------------------------------------------GammaSA
                                                          |       |       |       |                                                       |       |
                                                          |       |       |       |                                               +-131.0-|       +---------------------------------------------------------------GammaMSd
                                                          |       |       |       |                                               |       |
                                                          |       |       |       |                                               |       |                                                               +-------GammaMT
                                                          |       |       |       |                                               |       |                                                       +-963.0-|
                                                          |       |       |       |                                       +--72.0-|       +-------------------------------------------------150.0-|       +-------GammaMA
                                                          |       |       |       |                                       |       |                                                               |
                                                          |       |       |       |                                       |       |                                                               +---------------DeltaDP
                                                          |       |       |       |               +------------------61.0-|       |
                                                          |       |       |       |               |                       |       +-------------------------------------------------------------------------------GammaFB
                                                          |       |       |       |               |                       |
                                                          |       |       |       |               |                       +---------------------------------------------------------------------------------------GammaKKa
                                                          |       |       |       |               |
                                                          |       |       |       |               |                                                                                               +---------------GammaPT
                                                  +--14.0-|       |       |       |               |                                                                                       +-256.0-|
                                                  |       |       |       |       |               |                                                                                       |       |       +-------GammaPH
                                                  |       |       |       |       |               |               +-----------------------------------------------------------------939.0-|       +-373.0-|
                                                  |       |       |       |       |               |               |                                                                       |               +-------GammaABb
                                                  |       |       |       |       |               |               |                                                                       |
                                                  |       |       |       |       |               |               |                                                                       +-----------------------GammaPSf
                                                  |       |       |       |       |               |               |
                                                  |       |       |       |       |               |               |                                                                                       +-------GammaVAa
                                                  |       |       |       |       |               |               |                                                       +-------------------------965.0-|
                                                  |       |       |       |       |               |               |                                                       |                               +-------GammaVP
                                                  |       |       |       |       |               |               |                                                       |
                                                  |       |       |       |       |               |               |                                                       |                               +-------GammaPAb
                                                  |       |       |       |       |               |               |                                               +-560.0-|                       +-972.0-|
                                                  |       |       |       |       +----------5.00-|               |                                               |       |               +-924.0-|       +-------GammaVAb
                                                  |       |       |       |                       |               |                                               |       |               |       |
                                                  |       |       |       |                       |               |                                               |       |       +-997.0-|       +---------------GammaPSd
                                                  |       |       |       |                       |               |                                       +-640.0-|       |       |       |
                                                  |       |       |       |                       |               |                                       |       |       +-757.0-|       +-----------------------GammaPL
                                                  |       |       |       |                       |               |                                       |       |               |
                                                  |       |       |       |                       |               |                               +-549.0-|       |               +-------------------------------GammaVFb
                                                  |       |       |       |                       |       +-152.0-|                               |       |       |
                                                  |       |       +--2.00-|                       |       |       |                               |       |       +-----------------------------------------------GammaPPa
                                                  |       |               |                       |       |       |                       +-208.0-|       |
                                                  |       |               |                       |       |       |                       |       |       +-------------------------------------------------------GammaCP
                                                  |       |               |                       |       |       |                       |       |
                                                  |       |               |                       |       |       |                       |       |                                                       +-------GammaIB
                                                  |       |               |                       |       |       |                       |       +-------------------------------------------------985.0-|
                                                  |       |               |                       |       |       |               +-162.0-|                                                               +-------GammaIL
                                                  |       |               |                       |       |       |               |       |
                                                  |       |               |                       |       |       |               |       |                                               +-----------------------GammaSV
                                                  |       |               |                       |       |       |               |       |                                       +-365.0-|
                                                  |       |               |                       |       |       |               |       |                                       |       |       +---------------GammaGN
                                                  |       |               |                       |       |       |               |       |                                       |       +-961.0-|
                                                  |       |               |                       +--22.0-|       |       +--93.0-|       +---------------------------------258.0-|               |       +-------GammaAM
                                                  |       |               |                               |       |       |       |                                               |               +-422.0-|
                                                  |       |               |                               |       |       |       |                                               |                       +-------GammaGP
                                                  |       |               |                               |       |       |       |                                               |
                                                  |       |               |                               |       |       |       |                                               +-------------------------------GammaASc
                                                  |       |               |                               |       +-267.0-|       |
                                                  |       |               |                               |               |       |                                                                       +-------GammaVS
                                                  |       |               |                               |               |       +-----------------------------------------------------------------130.0-|
                                                  |       |               |                               |               |                                                                               +-------GammaMG
                                                  |       |               |                               |               |
                                                  |       |               |                               |               +---------------------------------------------------------------------------------------GammaHJ
                                                  |       |               |                               |
                                          +-115.0-|       |               |                               |                                                                                       +---------------GammaRN
                                          |       |       |               |                               +---------------------------------------------------------------------------------421.0-|
                                          |       |       |               |                                                                                                                       |       +-------GammaASa
                                          |       |       |               |                                                                                                                       +--1000-|
                                          |       |       |               |                                                                                                                               +-------GammaABa
                                          |       |       |               |
                                          |       |       |               |                                                                                                               +-----------------------GammaMAb
                                          |       |       |               |                                                                                                               |
                                          |       |       |               |                                                                                                       +-993.0-|               +-------GammaMAc
                                          |       |       |               |                                                                                                       |       |       +-378.0-|
                                          |       |       |               |                                                                                                       |       |       |       +-------GammaMSa
                                          |       |       |               |                                                                                                       |       +-564.0-|
                                          |       |       |               |                                                                                                       |               |       +-------GammaMMa
                                          |       |       |               |                                                                                                       |               +-695.0-|
                                          |       |       |               +--------------------------------------------------------------------------------------------------42.0-|                       +-------GammaMAa
                                          |       |       |                                                                                                                       |
                                          |       |       |                                                                                                                       |                       +-------GammaHSb
                                          |       |       |                                                                                                                       |               +-910.0-|
                                          |       |       |                                                                                                                       |       +-975.0-|       +-------GammaHSa
                                          |       |       |                                                                                                                       |       |       |
                                          |       |       |                                                                                                                       +-958.0-|       +---------------GammaHE
                                          |       |       |                                                                                                                               |
                                          |       |       |                                                                                                                               +-----------------------GammaCSa
                                          |       |       |
                                  +-855.0-|       |       |                                                                                                                                       +---------------GammaMMb
                                  |       |       |       |                                                                                                                               +-741.0-|
                                  |       |       |       |                                                                                                                               |       |       +-------GammaMSc
                                  |       |       |       |                                                                                                                       +-800.0-|       +-822.0-|
                                  |       |       |       |                                                                                                                       |       |               +-------GammaMP
                                  |       |       |       |                                                                                                                       |       |
                                  |       |       |       |                                                                                                               +-220.0-|       +-----------------------GammaMSb
                                  |       |       |       |                                                                                                               |       |
                                  |       |       |       |                                                                                                               |       |                       +-------GammaGS
                                  |       |       |       |                                                                                                       +--16.0-|       +------------------1000-|
                                  |       |       |       |                                                                                                       |       |                               +-------GammaPAd
                                  |       |       |       |                                                                                                       |       |
                                  |       |       |       |                                                                                                       |       |                               +-------GammaBN
                                  |       |       |       +--------------------------------------------------------------------------------------------------1.00-|       +-------------------------150.0-|
                          +-994.0-|       |       |                                                                                                               |                                       +-------BetaTD
                          |       |       |       |                                                                                                               |
                          |       |       |       |                                                                                                               |                                       +-------DeltaDAb
                          |       |       |       |                                                                                                               +---------------------------------213.0-|
                          |       |       |       |                                                                                                                                                       +-------DeltaGL
                          |       |       |       |
                          |       |       |       |                                                                                                                                                       +-------GammaETb
                          |       |       |       +-------------------------------------------------------------------------------------------------------------------------------------------------227.0-|
[truncated: 2,950 more chars]
