# Supplementary figures and images for: In silico evolutionary analysis of Helicobacter pylori outer membrane phospholipase A (OMPLA)
Source: BMC Microbiol. 2012 Sep 13;12:206. doi: 10.1186/1471-2180-12-206 (PMC3490997; doi:10.1186/1471-2180-12-206)

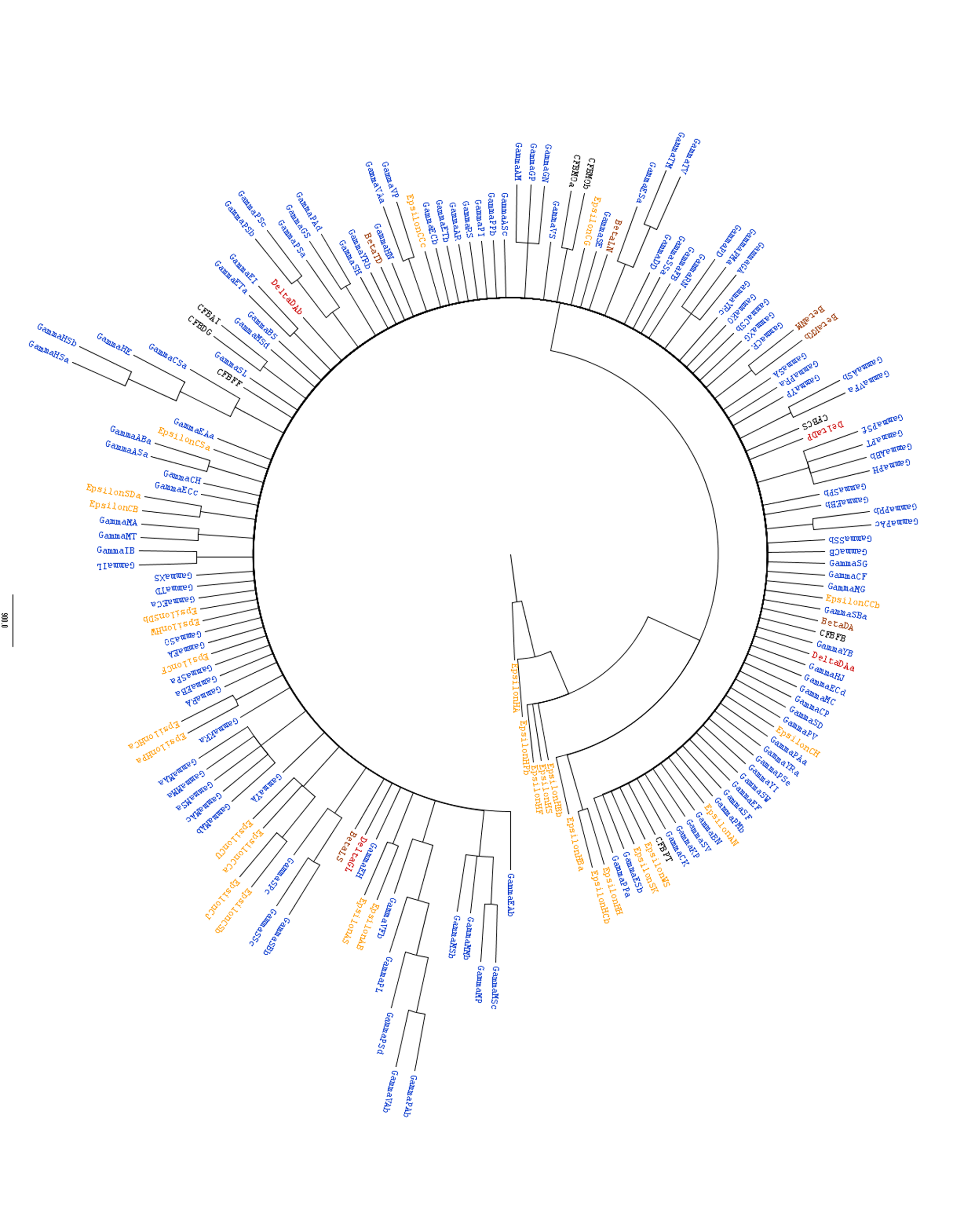

Supplement: Additional file 5 Figure S2 — Phylogenetic tree of Proteobacteria OMPLA sequences. Additional file 5 is a strict analysis of the OMPLA sequences found Figure 3. In this analysis, a higher threshold is used where only groups occurring more than 75% is included (M75). (PNG 1253 kb) [file 1471-2180-12-206-S5.png]

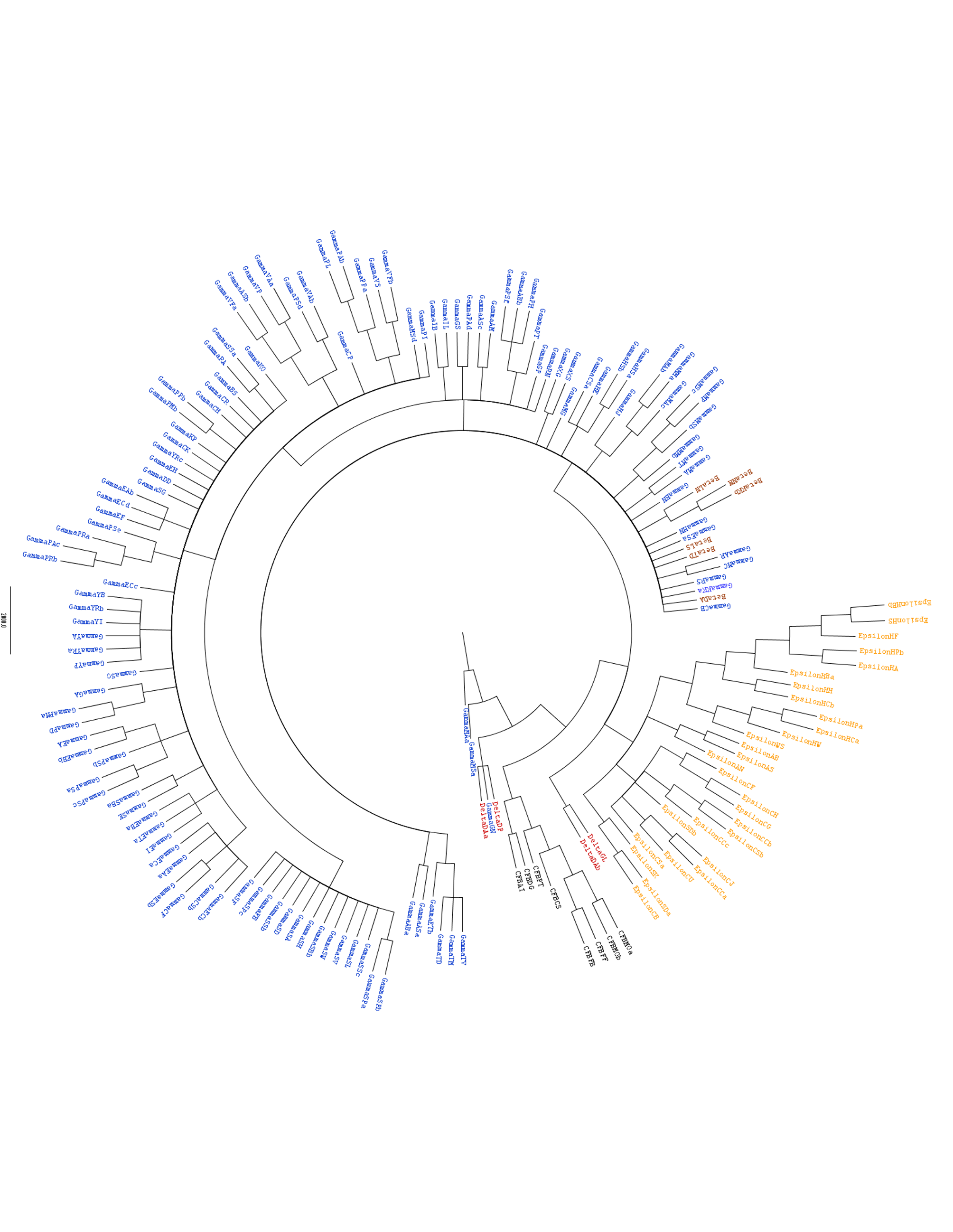

Supplement: Additional file 6 Figure S3 — Phylogenetic tree of Proteobacteria AtpA sequences. Additional file 5 is a strict analysis (M75) of the OMPLA sequences found Figure 4. (PNG 903 kb) [file 1471-2180-12-206-S6.png]

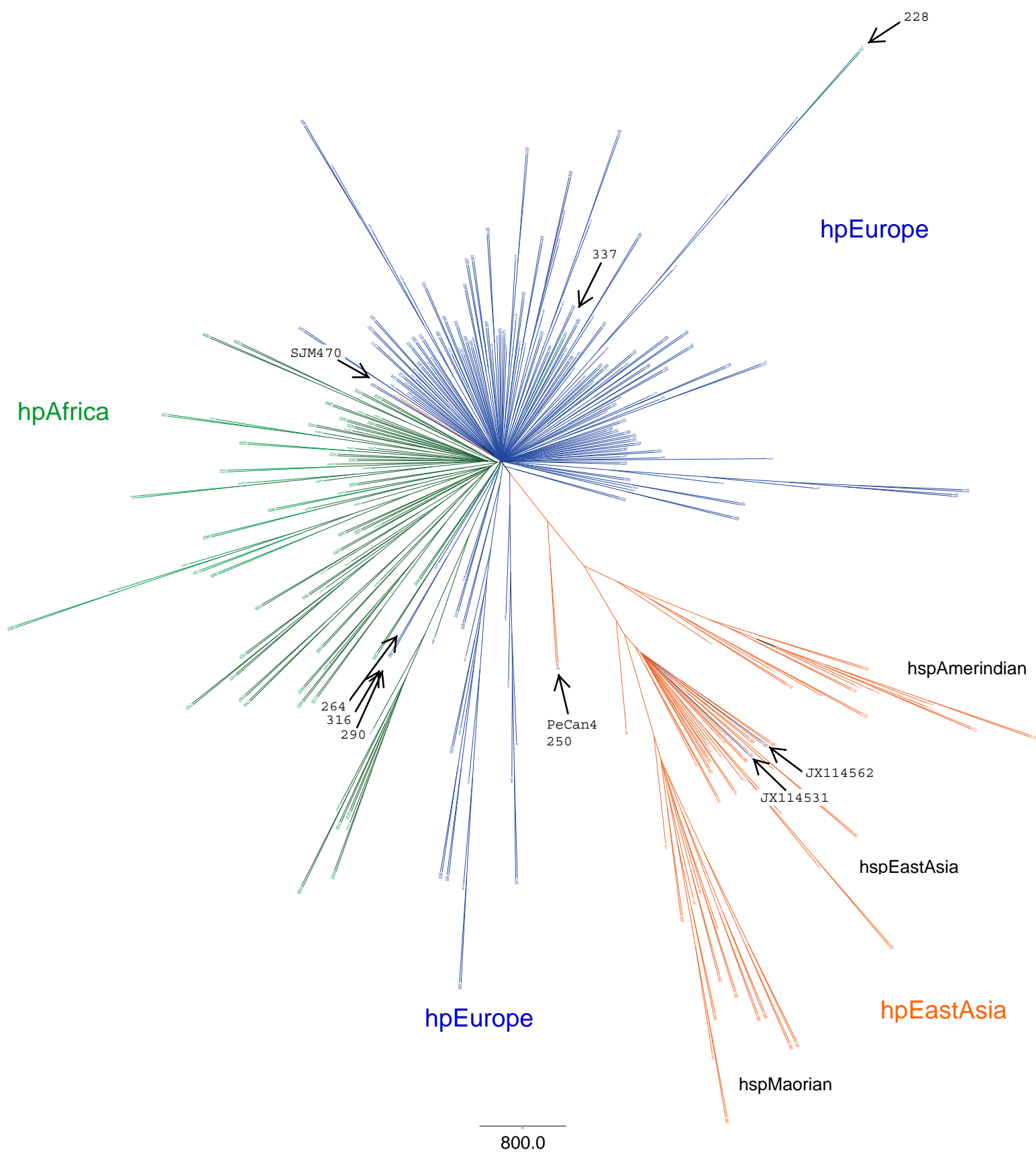

Supplement: Additional file 7 Figure S1 — Phylogenetic tree of H. pylori housekeeping sequences. Additional file 7 supplements Figure 1 with complete labelling. [file 1471-2180-12-206-S7.pdf]
